# Fibrinogen species as resolved by HPLC-SAXS data processing within the *UltraScan Solution Modeler* (*US-SOMO*) enhanced SAS module

**DOI:** 10.1107/S0021889813027751

**Published:** 2013-11-15

**Authors:** Emre Brookes, Javier Pérez, Barbara Cardinali, Aldo Profumo, Patrice Vachette, Mattia Rocco

**Affiliations:** a Department of Biochemistry, University of Texas Health Science Center at San Antonio, San Antonio, TX 78229-3900, USA; b Beamline SWING, Synchrotron SOLEIL, L’Orme des Merisiers, BP48, Saint-Aubin, Gif sur Yvette, France; c Biopolimeri e Proteomica, IRCCS AOU San Martino-IST, Istituto Nazionale per la Ricerca sul Cancro, Genova, Italy; d Department of Pathology and Laboratory Medicine, University of North Carolina at Chapel Hill, Chapel Hill, NC, USA; e Institut de Biochimie et de Biophysique Moléculaire et Cellulaire, CNRS UMR 8619, UPS 11, Orsay, France; f Université Paris-Sud 11, Bâtiment 430, Orsay, France

**Keywords:** small-angle scattering, chromatography, HPLC-SAXS, multi-resolution modeling, bovine serum albumin, fibrinogen

## Abstract

Fibrinogen is a large heterogeneous aggregation/degradation-prone protein playing a central role in blood coagulation and associated pathologies, whose structure is not completely resolved. When a high-molecular-weight fraction was analyzed by size-exclusion high-performance liquid chromatography/small-angle X-ray scattering (HPLC-SAXS), several composite peaks were apparent and because of the stickiness of fibrinogen the analysis was complicated by severe capillary fouling. Novel SAS analysis tools developed as a part of the *UltraScan Solution Modeler* (*US-SOMO*; http://somo.uthscsa.edu/), an open-source suite of utilities with advanced graphical user interfaces whose initial goal was the hydrodynamic modeling of biomacromolecules, were implemented and applied to this problem. They include the correction of baseline drift due to the accumulation of material on the SAXS capillary walls, and the Gaussian decomposition of non-baseline-resolved HPLC-SAXS elution peaks. It was thus possible to resolve at least two species co-eluting under the fibrinogen main monomer peak, probably resulting from in-column degradation, and two others under an oligomers peak. The overall and cross-sectional radii of gyration, molecular mass and mass/length ratio of all species were determined using the manual or semi-automated procedures available within the *US-SOMO* SAS module. Differences between monomeric species and linear and sideways oligomers were thus identified and rationalized. This new *US-SOMO* version additionally contains several computational and graphical tools, implementing functionalities such as the mapping of residues contributing to particular regions of *P*(*r*), and an advanced module for the comparison of primary *I*(*q*) *versus q* data with model curves computed from atomic level structures or bead models. It should be of great help in multi-resolution studies involving hydrodynamics, solution scattering and crystallographic/NMR data.

## Abstract 2

The usefulness of a new high-performance liquid chromatography/small-angle X-ray scattering (HPLC-SAXS) data analysis module within the multi-resolution modeling suite *US-SOMO* is illustrated with size-exclusion small-angle X-ray scattering (SE-SAXS) data of a crude bovine serum albumin sample. The module is then applied to the SE-SAXS study of a human plasma fibrinogen high-molecular-weight fraction presenting severe aggregation problems and a split non-symmetrical main elution peak probably resulting from in-column degradation.

## 1. Introduction

Advances in molecular medicine and personalized therapies depend on the identification of interactions between biomacromolecules and on the understanding of their structure–function relationships. Structural genomics projects (*e.g.* Burley *et al.*, 1999 ▶; Todd *et al.*, 2005 ▶; see also Smith *et al.*, 2007 ▶) are producing new and more refined three-dimensional structures, from isolated domains to entire proteins, nucleic acids and their complexes. However, an exhaustive list of every relevant structure and of its complexes with its many partners is unlikely to be attained with the current high-resolution methods (X-ray crystallography/NMR) alone. Intermediate-resolution techniques have evolved to complement the higher-resolution data, like cryo-electron microscopy (van Heel *et al.*, 2000 ▶), electron tomography (McEwen & Marko, 2001 ▶) and small-angle X-ray/neutron scattering (Svergun & Koch, 2003 ▶), all able to provide three-dimensional envelopes at ∼10–20 Å resolution. Starting from the atomic structures of the components, a typical task is to place them correctly within the envelope (Wriggers *et al.*, 1999 ▶; Suhre *et al.*, 2006 ▶; Topf *et al.*, 2008 ▶) or to optimize their arrangement to fit experimental scattering data (Petoukhov & Svergun, 2005 ▶). Lower-resolution conformational and hydrodynamic parameters, such as the radius of gyration (*R*_g_), the translational diffusion coefficient (*D*_t_), the sedimentation coefficient (*s*), the rotational correlation time (τ_c_) and the intrinsic viscosity ([η]), can be used to refine the models further by comparing experimental and calculated parameters and selecting the best matching models (Byron, 2000 ▶). Among the intermediate-resolution techniques, those utilizing the small-angle scattering from X-rays (SAXS) or neutrons (SANS) have the distinct advantage of allowing samples to be examined in near-physiological conditions, though they only provide ensemble-averaged data and thus often require extensive modeling for correct interpretation (Mertens & Svergun, 2010 ▶; Putnam *et al.*, 2007 ▶). A popular suite of computer programs covering from data reduction to modeling for both SAXS and SANS is the *ATSAS* suite developed by the Svergun group at EMBL in Hamburg, Germany (see Petoukhov *et al.*, 2012 ▶; http://www.embl-hamburg.de/biosaxs/software.html). However, the SAS field is undergoing rapid developments, such as the availability of high-performance liquid chromatography (HPLC)-SAXS setups (*e.g.* Mathew *et al.*, 2004 ▶; reviewed by Pérez & Nishino, 2012 ▶), calling for the implementation of additional analysis and modeling tools.

Fibrinogen (FG) is a rod-like very elongated (*L* ≃ 460 Å, *d* ≃ 50 Å) high-molecular-weight (∼338 000) protein, which plays a central role in the blood coagulation system (see Weisel, 2005 ▶) and is associated with several pathologies such as thrombosis and cancer (Blombäck, 1996 ▶; Boccaccio & Medico, 2006 ▶). It is composed of two pairs each of three different chains, Aα, Bβ and γ, whose N-terminal ends constitute a central globular domain. Two symmetrical pairs of triple-coiled coils depart from the central domain, connecting it to two outer globular domains, each containing the C-terminal ends of both the Bβ and the γ chains (see Weisel, 2005 ▶). The ∼400 C-terminal residues of the Aα chains (αC domains) are instead likely to be mostly disordered, and, being very sensitive to proteolytic cleavage, they are the major source of heterogeneity of circulating FG (Mosesson, 1983 ▶). The FG three-dimensional structure is only partially known (see Kollman *et al.*, 2009 ▶, and references therein), and in particular the conformation and spatial location of the αC domains is a subject of much debate (*e.g.* Yang *et al.*, 2001 ▶; Litvinov *et al.*, 2007 ▶; Tsurupa *et al.*, 2009 ▶). As a part of an ongoing study aimed at characterizing nearly intact FG and αC domain-less species (see Cardinali *et al.*, 2010 ▶; Raynal *et al.*, 2013 ▶), and their covalent and noncovalent adducts, we have performed size-exclusion (SE) HPLC-SAXS studies on a human plasma high-molecular-weight FG fraction (hpHMW-FG). This material presents severe aggregation problems, and the SE-HPLC-SAXS analysis showed non-baseline-resolved oligomers peaks and a split non-symmetrical main peak.

Spurred by the need for proper analysis of the SE-HPLC-SAXS hpHMW-FG data, we have implemented a set of novel utilities for SAS data analysis and modeling within the *UltraScan Solution Modeler* (*US-SOMO*; http://somo.uthscsa.edu/), a suite of open-source computer programs under a graphical user interface (GUI) that was originally developed for computing conformational and hydrodynamic parameters of biomacromolecules starting from their three-dimensional atomic structure (Brookes, Demeler, Rosano & Rocco, 2010 ▶). *US-SOMO*’s hydrodynamic modeling is based on accurate methods developed by the Rocco and Byron laboratories, preserving the correspondence between the atoms in the original biomacromolecule and the low-resolution beads used to represent it (Byron, 1997 ▶; Rai *et al.*, 2005 ▶). The new SAS tools have evolved from a small initial nucleus that we had previously reported (Brookes, Demeler & Rocco, 2010 ▶), providing an integrated framework with existing hydrodynamics tools. On the data analysis side, prominent is a module for the conversion of HPLC-SAXS scattering intensity as a function of the scattering vector [*I*(*q*) *versus q*] data frames into scattering intensity as a function of elution frame/time [*I*(*t*) *versus t*] at each scattering vector magnitude *q* (*q* = 4πsinθ/λ, with 2θ the scattering angle and λ the incident radiation wavelength). This allows the Gaussian decomposition of HPLC-SAXS data, resolving peaks that are not baseline separated (usually first shown at the concentration profile level), followed by back generation of *I*(*q*) *versus q* data frames for each decomposed peak. This is of particular importance, given the sensitivity of SAXS to polydispersity; to the best of our knowledge, commercial packages such as *PeakFit* (Systat Software, San José, CA, USA; http://www.sigmaplot.com), while providing very advanced functions, do not offer the global fitting of multiple data sets required to properly analyze the hundreds of *I*(*t*) *versus t* ‘chromatograms’ resulting from HPLC-SAXS experiments. Single-value decomposition (SVD) methods (*e.g.* Williamson *et al.*, 2008 ▶; Lawson *et al.*, 1995 ▶; Aster *et al.*, 2005 ▶) can be also applied on the *I*(*q*) *versus q* data sets, as a means to properly initialize the number of Gaussians used for *I*(*t*) *versus t* peak(s) decomposition. Furthermore, problematic data sets with drifting baselines, which might be caused by the accumulation of material on the SAXS capillary walls during the continuous flow required for chromatography, can be suitably corrected by defining and subtracting baselines in the frame/time domain. Routines for the semi-automated extrapolation of the overall, cross-sectional and transverse *z*-average radii of gyration 〈*R*_g_^2^〉*_z_*, 〈*R*_c_^2^〉*_z_* and 〈*R*_t_^2^〉*_z_*, and of the weight-average zero-scattering angle intensities 〈*I*(0)〉_w_, 〈*I*_c_(0)〉_w_ and 〈*I*_t_(0)〉_w_, and hence the molecular mass 〈*M*〉_w_, mass/length ratio 〈*M*/*L*〉_w/*z*_ and mass/area ratio 〈*M*/*A*〉_w/*z*_ from Guinier plots, are available. Not utilized in the present study, but implemented in *US-SOMO*, are many additional functionalities that will be briefly mentioned in §[Sec sec3.1]3.1 below.

The HPLC-SAXS *US-SOMO* module was first tested on SE-HPLC-SAXS data collected on a crude bovine serum albumin sample containing a large number of trimer and dimer species that was used to verify the SE columns’ performance, and then applied to the hpHMW-FG data. In both cases baseline correction and Gaussian decomposition were employed. In particular, the analysis allowed us to distinguish between side-by-side and linear aggregates in the hpHMW-FG oligomers peaks, and to characterize the two components of the main peak as having nearly identical conformation while probably differing by the presence/absence of a relevant portion of the αC domains.

## 2. Materials and methods

### 2.1. HPLC-SAXS

All chemicals were reagent grade from Merck (VWR International, Milan, Italy; http://www.merckmillipore.com/), unless otherwise stated, and double-distilled or MilliQ water was used in the preparation of all the solutions. For HPLC-SAXS, the buffer used was TBS [Tris-buffered saline; tris(hydroxymethyl)aminomethane 50 m*M*, NaCl 104 m*M*, aprotinin 10 kallikrein inhibitor units (KIU) per millilitre, pH 7.4]. Aprotinin and bovine serum albumin (BSA; a >10 year old Cohn fraction V sample) were from Sigma–Aldrich (St Louis, MO, USA; http://www.sigmaaldrich.com/sigma-aldrich/home.html). The human plasma FG fraction enriched in full-length material (hpHMW-FG) was purified and characterized as previously described (Cardinali *et al.*, 2010 ▶). SE-HPLC was performed on two 7.8 × 300 mm columns packed with hydroxylated polymethacrylate particles (TSK G4000PW_XL_, 10 µm size, 500 Å pore size, and G3000PW_XL_, 6 µm size, 200 Å pore size, Tosoh Bioscience, Tokyo, Japan; http://www.tosohbioscience.com/) connected in series, protected by a 6 × 40 mm guard column filled with G3000PW resin (Tosoh). The Agilent chromatographic system of the SWING beamline at the synchrotron SOLEIL (David & Pérez, 2009 ▶) was operated at 0.35 ml min^−1^ flow rate. The columns and the SAXS flow cell were maintained at 293.2 ± 0.1 K. BSA was dissolved at 9 mg ml^−1^ in TBS, centri-filtered at 12 000 r min^−1^ over 0.22 µm cellulose acetate filters (Costar Spin-X, Sigma–Aldrich), and 60 µl (two replicates) were then injected into the SE columns. The hpHMW-FG concentration was 17.3 mg ml^−1^ in TBS, and after centri-filtration, 20 or 50 µl were injected into the SE columns. SAXS data (λ = 1.03 Å) were collected at a ∼4 m sample–detector distance, accessing a *q* range of 0.0023–0.2750 Å^−1^, normalized to the intensity of the transmitted beam, background subtracted on the SWING beamline using the local dedicated program *Foxtrot*, and put on an absolute scale using the scattering by water within the *US-SOMO* SAS module. Extinction coefficients (*E* ^280^) and partial specific volumes (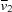) were calculated by *PROMOLP* (Spotorno *et al.*, 1997 ▶). For BSA, *E* ^280^ = 0.65 ml mg^−1^ cm^−1^ and 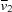 = 0.733 ml g^−1^. For the injected hpHMW-FG samples, the values were computed taking into account the inherent polydispersity (Raynal *et al.*, 2013 ▶), and were *E* ^280^ = 1.55 ml mg^−1^ cm^−1^ and 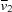 = 0.715 ml g^−1^. Sample analyses by polyacrylamide gel electrophoresis (PAGE) in the presence of sodium dodecyl sulfate (SDS) without or with urea, and western blotting, all followed by densitometry, were performed as previously reported (Cardinali *et al.*, 2010 ▶).

### 2.2. Software implementation

The *US-SOMO* technical specifications have already been described (Brookes, Demeler, Rosano & Rocco, 2010 ▶, 2012 ▶). The current software is a GUI application written in *C*++ utilizing *Qt* (http://qt-project.org/). The code is multi-platform, with binaries available for Linux, Mac OSX and Windows. The source code is available *via* a wiki integrated subversion repository, which can be found from the main *US-SOMO* web page. The current user base includes ∼700 registered individual researchers and 56 registered laboratories worldwide.

## 3. *US-SOMO* SAS module

### 3.1. Main panel

**Figure 1:**
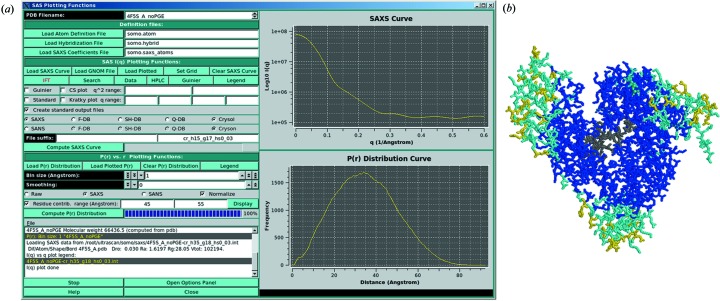
(*a*) The renewed GUI of the *US-SOMO* SAS module main panel. In the graphic windows, the *I*(*q*) *versus q* and the *P*(*r*) *versus r* curves computed from the BSA crystal structure 4f5s (Bujacz, 2012 ▶) using *Crysol* and the *US-SOMO* internal SAXS method, respectively, are shown. (*b*) A snapshot of the *RasMol*-produced BSA structure with the residues contributing to the chosen *P*(*r*) *versus r* range, 45–55 Å, color coded from yellow to blue in order of decreasing importance.

The new GUI of the *US-SOMO* SAS module is shown in Fig. 1 ▶(*a*). It is divided in two halves, the top one for reciprocal-space operations and the bottom one for real-space operations. Among the reciprocal-space operations, *I*(*q*) *versus q* SAXS and SANS curves can be computed from atomic level structures, either with explicit hydration (which should be externally provided; see *e.g.* Poitevin *et al.*, 2011 ▶) using the Debye equation (Glatter & Kratky, 1982 ▶) and its variant computed with spherical harmonics (Stuhrmann, 1970 ▶; Stuhrmann *et al.*, 1977 ▶; Svergun & Stuhrmann, 1991 ▶), or with implicit hydration, as in *Crysol* (Svergun *et al.*, 1995 ▶), *Cryson* (Svergun *et al.*, 1998 ▶) and a fast Debye method based on the *FoXS* concept (Schneidman-Duhovny *et al.*, 2010 ▶). Guinier analyses can be performed in manual or semi-automatic mode. A primary data reduction utility with the ability to perform buffer subtractions, normalization and curve joining is also present (to be described in a future publication). In addition, we have developed a novel module for the processing of HPLC-SAXS data, allowing a first-order correction for spurious background intensity arising from capillary fouling, and application of Gaussian decomposition to non-baseline-resolved SAXS peaks (see below, and the supplementary material^1^). In the real-space section, *P*(*r*) *versus r* curves can be computed directly from atomic level structures for both SAXS and SANS approaches, and compared with data derived by inverse Fourier transformation of reciprocal-space data. To help in understanding how the distribution of residues in a macromolecule affects the *P*(*r*) *versus r* distribution, a novel tool was developed, allowing visualization (using *RasMol*; Sayle & Milner-White, 1995 ▶) of the structure with its residues color coded according to their contribution to a particular distance range. In Fig. 1 ▶(*b*), a BSA structure is visualized, colour coded to show which residues contribute the most to the *P*(*r*) *versus r* curve in the 45–55 Å range (yellow to blue in decreasing order; gray, no contribution). All synthetic curves can be ranked against or combined to yield a best-fitting curve with experimentally derived data using a nonnegative least-squares fitting routine. Both reciprocal- and real-space curves can also be computed starting from lower-resolution bead models. Finally, conformational variability and local or segmental flexibility can be taken into account by using a discrete molecular dynamics (Ding & Dokholyan, 2006 ▶; Dokholyan *et al.*, 1998 ▶) utility running remotely on several supercomputer clusters. The supplementary material contains a full description of the *US-SOMO* SAS module’s many features.

### 3.2. The *US-SOMO* HPLC-SAXS and other modules

A relatively recent advance in biomacromolecular SAXS has been the direct coupling of the eluate from HPLC columns (mainly SE-HPLC) to a flow-through SAXS capillary, enabling data collection at regular intervals (slices/frames) during the chromatographic separation (Mathew *et al.*, 2004 ▶; David & Pérez, 2009 ▶). This usually allows the separation of pure, essentially monodispersed samples on which the SAXS data are collected. However, baseline resolution between species cannot always be achieved, and/or other problems might arise, such as capillary fouling, making it difficult both to analyze and to interpret the data. To tackle these issues, we have developed an ‘HPLC’ module that contains a number of features which are fully described in the supplementary material. All of the *US-SOMO* SAS module’s available options, accessible by pressing the ‘Open Options Panel’ button at the bottom of the main panel (Figs. 1*a* ▶ and S1), are also described in the supplementary material (Figs. S4–S11).

## 4. Results and discussion

### 4.1. Testing the *US-SOMO* HPLC-SAXS module with BSA data

A relatively crude, old BSA sample having a substantial number of dimers and trimers was run before the hpHMW-FG samples, mainly to test the columns’ efficiency. The SE-HPLC-SAXS data acquired on this BSA sample were then used to verify the performance of the *US-SOMO* HPLC-SAXS module, and many images taken from a typical processing run are used in the supplementary material to describe the module (Figs. S12–S15, S19, S21, S22 and S24–S30). Since a typical HPLC-SAXS experiment produces a series of *I*(*q*) *versus q* data collected at some time interval (‘frames’), they can be inserted into a two-dimensional matrix where each line corresponds to a frame number (or time value) and the columns contain the intensities *I*(*q*) and their associated standard deviations (SDs) at the various scattering angles *q*. Transposition generates another matrix where the lines correspond to the *q* values and each column contains the intensities *I*(*t*) (and their associated SDs) corresponding to each frame number (or time value). As shown in Fig. S13, the first information that can be revealed by the *I*(*t*) *versus t* chromatograms is that, after the protein peaks, the baseline might not return to the initial value [note that the buffer contribution, first evaluated by averaging a number of frames taken well before the column void volume, was already subtracted from the SAXS *I*(*q*) *versus q* data at each frame]. This is most likely due to biological material aggregated by the intense X-ray beam on the capillary cell walls. While this type of problem is preferentially dealt with at the experimental level (see note 2 below), this is not always possible. In such cases, as a first approximation we can assume a linear increase over time of the material deposited on the capillary. This allows the definition of baselines for each *q*-value chromatogram (see Fig. S14), which can then be subtracted (see Fig. S15). If necessary, Gaussian decomposition can be performed on this baseline-subtracted data set (see Figs. S19, S21, S22 and S24–S27). SVD can also be performed either on the original or, if baseline subtraction was performed, on a reconstructed *I*(*q*) *versus q* data set, for instance to decide how many Gaussians should be used to decompose each peak (see the hpHMW-FG section; not needed in this very simple situation). Importantly, the position and width of each Gaussian in a ‘family’ (*i.e.* the Gaussians in all *q*-value chromatograms fitting a particular chromatographic peak in the time or frame domain) must have the same values, and only the amplitudes are fitted. This is done by first optimizing these parameters on a subset of the *q*-value chromatograms (see Figs. S21, S22 and S24), with optional SD weighting (recommended), and then globally applying them to all *q*-value chromatograms (see Figs. S25–S26). In Fig. S25, the nonrandom distribution of residuals around frames 130–150 arises from the tail ends of the chromatograms that are not well fitted by pure Gaussian functions. In a future development, modified Gaussian functions able to cope with skewed profiles will be introduced.

The concentration monitor data (either absorbance or refractive index are supported) can then be decomposed after rescaling and time shifting (if necessary) for proper alignment with the SAXS data (see Figs. S28 and S29). The decomposition is done using the same number of Gaussians employed to fit each *q*-value chromatogram, keeping their widths fixed, if necessary allowing just a small change (2–4%) in their position to compensate for potential misalignments, and fitting the amplitudes (Fig. S30). Note that, if significant band broadening occurs between the concentration and SAXS detectors, it is not possible to fit the concentration signal keeping the Gaussian widths fixed. Band broadening correction routines will be implemented to cope with this issue.

Either right after baseline correction (if necessary) or after Gaussian decomposition, it is possible to back generate *I*(*q*) *versus q* data sets for each Gaussian in each frame, by back transposition of the data matrix. Generating data directly from the Gaussians produces smoothed data sets, which might hide potential problems. Therefore, the default option in the *US-SOMO* HPLC-SAXS module is to produce data as a percentage of the original curve based on the contribution of each Gaussian to that particular point in the *I*(*t*) *versus t* curves, with SDs also assigned proportionally. Finally, if a concentration curve and its Gaussian decomposition have been associated with the *I*(*t*) *versus t* data, it is possible to compute the fractional concentration for each resulting *I*(*q*) *versus q* decomposed frame. This is done by entering an extinction coefficient (or a d*n*/d*c*, if a refractive index monitor was used) for each Gaussian (see Fig. S31), and the module will associate it with each resulting *I*(*q*) *versus q* frame. To compute 〈*M*〉_w_, 〈*M*/*L*〉_w/*z*_ and 〈*M*/*A*〉_w/*z*_, partial specific volumes can also be associated with each Gaussian at this stage (Fig. S31), and are likewise carried over to the resulting *I*(*q*) *versus q* frames. Different values can be entered for each Gaussian in case the experimental data contain multiple species, but they can be set to equal values for the more general case of a single species having multiple conformations or different association states.

**Figure 2:**
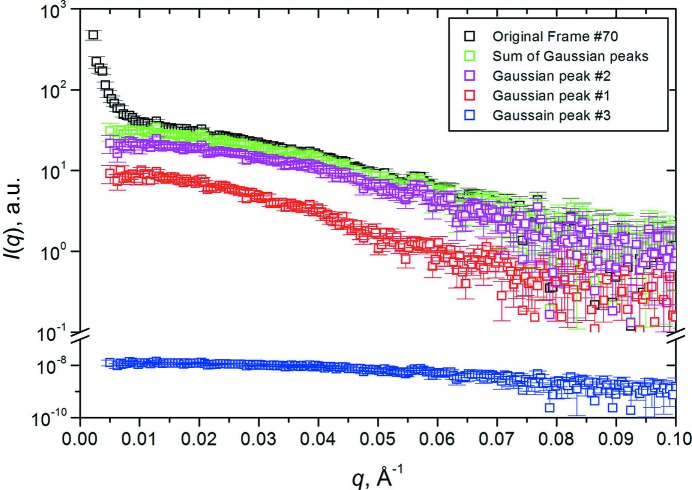
Original frame #70 (top, black squares) of the SE-HPLC-SAXS BSA analysis (see Fig. S26), sum of the resulting *I*(*q*) *versus q* back generated from the Gaussians (green squares), and the contributions *I*(*q*) *versus q* of individual Gaussians for peak #2 (dimer; magenta squares) and peak #1 (trimer; red squares). Gaussian peak #3 (monomer; bottom, blue squares), does not contribute significantly to this frame.

To demonstrate the performance of the baseline correction and Gaussian decomposition, we have chosen a region of the BSA chromatogram where trimers and dimers are not well separated. In Fig. 2 ▶, we show the results of the baseline-corrected Gaussian decomposition for chromatographic frame #70 (see Fig. S26), with the produced *I*(*q*) *versus q* frames computed as a percentage of the original curve. Note how the baseline subtraction has removed the upturn present on the original data at *q* < 0.01 Å^−1^, and the correct absence of any significant contribution from peak #3. If the baseline is added back, the ‘sum of Gaussians’ curve (green) will be completely superimposed on the original frame (not shown for clarity). The concentrations, *q* ranges, fit standard error and derived [〈*R*_g_^2^〉*_z_*]^1/2^ and 〈*M*〉_w_ values for the original and baseline-subtracted frame #70, and for the Gaussian peaks (G-pk) #1 and #2 resulting from its decomposition, are shown in Table 1 ▶. They can also be compared with the values obtained for the top chromatography peak frames of the two components (see Fig. S26), #50 and #81. In addition, the top peak frame of the BSA monomer, #125, has been analyzed. A first observation is that for the first two peaks the baseline subtraction either alone or followed by Gaussian decomposition yields 〈*M*〉_w_ values lower than those derived from the unprocessed frames. This is understandable since in the Guinier analysis the clear upturn at very low *q* values seen in the original frames (see Fig. S12) could still have nonnegligible contributions in the *q* range used for the linear regression. A second observation is that the BSA monomer 〈*M*〉_w_ values, ∼75–77 000 g mol^−1^, are about 15% higher than that deduced from the sequence, 66 283 g mol^−1^. Averaging the top ten frames of peak #3 produced better statistics, but did not significantly change these values (data not shown). This result is confirmed by the 〈*M*〉_w_ values for the BSA dimers (Table 1 ▶, frame #81), which are 6–15% higher than the expected value of ∼132 600 g mol^−1^, and partially also at the trimer level (Table 1 ▶, frame #50), where, however, the very low amount of material present makes it difficult to determine a correct 〈*M*〉_w_. In particular, for frame #50, the resulting 〈*M*〉_w_ from the baseline-subtracted and G-pk #1 *I*(*q*) curves differ significantly, and both differ from the unprocessed frame’s resulting 〈*M*〉_w_. This suggests an inadequate baseline subtraction in this very noisy low-intensity zone. More advanced, flexible baseline-subtraction routines will be implemented in the near future. As for the general 〈*M*〉_w_ issues, serum albumins are well know to bind a wide variety of ligands (*e.g*. Peters, 1985 ▶; Fasano *et al.*, 2005 ▶), and a relatively crude (for instance, not fatty acid depleted), old BSA stock was used because we were mainly interested in having enough trimers and dimers for column efficiency tests. Therefore, this large discrepancy at the 〈*M*〉_w_ level is not surprising and could also result from the combined effect of changes in the global extinction coefficient and partial specific volume of the putative BSA–ligand(s) complexes. Since the purpose of the BSA tests was not directed at checking the accuracy of the 〈*M*〉_w_ determination in our setup, this matter was not further investigated. As for the conformational parameters, for the main monomer top peak frame #125 the extrapolated [〈*R*_g_^2^〉*_z_*]^1/2^ values from the unprocessed, baseline-subtracted and G-pk #3 data are all in very good agreement with the value of 27.7 Å that can be computed using *Crysol* from the BSA three-dimensional structure (Bujacz, 2012 ▶). More importantly, for frame #70, where there is a significant contribution of both G-pks #1 and #2, the decomposition yields [〈*R*_g_^2^〉*_z_*]^1/2^ values that are very close to those derived from the top peak processed frames #50 and #81, while the unprocessed or baseline-subtracted frame yields just an average of the two values, as expected. Given the low intensity level of the data in this region, we find this result to be quite satisfactory.

To summarize this section, the usefulness of the *I*(*t*) *versus t* conversion was first demonstrated by the visualization of the capillary fouling evidence, and the basic principles of baseline correction and Gaussian decomposition followed by *I*(*q*) *versus q* restoration were implemented and successfully tested. However, further improvements, especially for the baseline subtraction and the treatment of the concentration monitor data, would probably be beneficial.

### 4.2. Fibrinogen HPLC-SAXS

**Figure 3:**
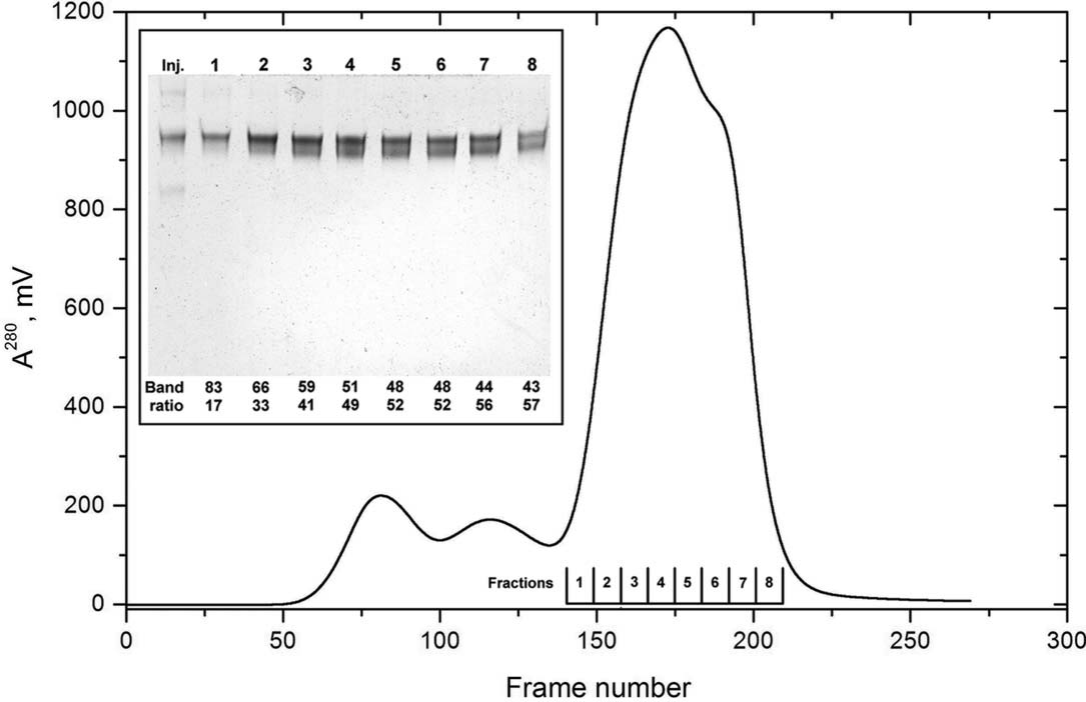
(Main panel) UV chromatographic profile of an SE-HPLC-SAXS analysis of hpHMW-FG (20 µl at 17.3 mg ml^−1^ in TBS were injected). (Inset) SDS/urea–PAGE analysis of the starting material (Inj.) and of the fractions collected on a duplicate run after disconnection from the SAXS setup (100 µl at ∼9 mg ml^−1^ were injected). Fractions are indicated at the bottom of the main panel. Their concentration was determined, and equal amounts (∼1.9 µg for fractions 2–7, but only ∼1.2 µg for fractions 1 and 8) of not-reduced samples were loaded in the wells of a 10 × 8 cm 1.5 mm-thick 3.2% T–5% C polyacrylamide SDS/urea gel, electrophoresed, stained with Coomassie blue and subjected to densitometric analyses (see Cardinali *et al.*, 2010 ▶). The fractional concentrations of the two main bands expressed in % are reported at the bottom of each lane.

The SE-HPLC-SAXS of the hpHMW-FG preparation presented several problems. To begin with, this FG fraction has a strong tendency to aggregate, especially during freeze–thaw operations. Even after high-speed centrifugation and centri-filtration, large aggregates were still present and contributed to a broad peak eluting near the void volume of our HPLC columns, as shown in the UV trace in Fig. 3 ▶. This is followed by a minor non-baseline-resolved species, and the main peak presents a prominent shoulder after the maximum. Furthermore, capillary fouling as the run progressed, notwithstanding all common precautions taken, was evident in a similar way to what is shown in the supplementary material for the BSA run used as an example (see Fig. S13). Without baseline correction and Gaussian decomposition, it would have been difficult to extract good quality data from this run.^2^

**Figure 4:**
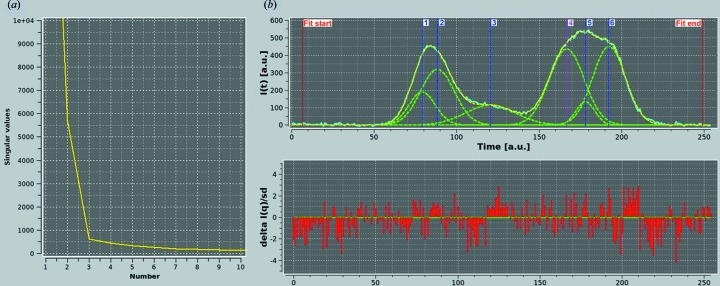
(*a*) Plot of the first ten singular values *versus* value number derived from SVD analysis of the baseline-subtracted reconstructed *I*(*q*) *versus q* for the hpHMW-FG SE-HPLC-SAXS data set (*q* = 0.0030–0.170 Å^−1^). (*b*) (Top graph) A single *I*(*t*) *versus t* chromatogram for *q* = 0.0058 Å^−1^ (cyan), with the six fitting Gaussians (green curves, numbered 1–6 from left to right). The yellow curve is the sum of the Gaussians. (Bottom graph) The fit-associated reduced residuals.

**Figure 5:**
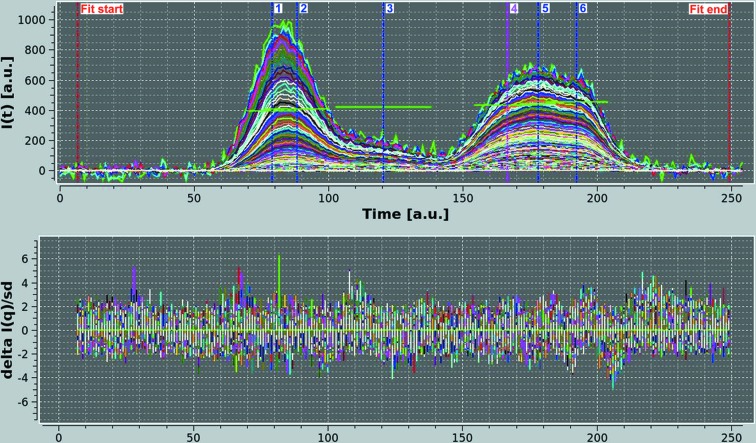
(Top graph) Global Gaussians of the hpHMW-FG SE-HPLC-SAXS data [664 *I*(*t*) *versus t* data sets from *q* = 0.0030 Å^−1^ to *q* = 0.170 Å^−1^]. Six Gaussians were employed to fit the data, whose centers and widths are indicated by the vertical blue and magenta lines and by the green horizontal bars, respectively. (Bottom graph) The fit-associated reduced residuals.

After baseline definition and subtraction (not shown), SVD followed by Gaussian analysis were performed. SVD was done on a reconstructed *I*(*q*) *versus q* data set, to avoid fitting the baseline drift. As shown in Fig. 4 ▶(*a*), at least four components, possibly five, are making relevant contributions to the data. In the end, however, six Gaussians were found to be necessary to produce a reasonably good fit of the *I*(*t*) *versus t* chromatograms. In Fig. 4 ▶(*b*), the results for a single *q* value are shown, and the contribution of the five ‘major’ G-pks (#1–4, #6) is evident. However, without the small G-pk (#5) positioned between the two principal G-pks (#4 and #6) under the main chromatographic peak, the fit is significantly worse (data not shown). The results of the global fit and global Gaussian operations have produced very nicely fitting Gaussians for all the peaks in all the *I*(*t*) *versus t* chromatograms examined (*q* range 0.00302–0.170 Å^−1^, above which noise dominates), as shown in Fig. 5 ▶. Note how the residuals are quite low (mostly within 2 SDs) when considering the noise present, especially at very low *q* values, and evenly distributed (except at the very beginning and end of the chromatograms). However, it must be pointed out that given the nature of the Gaussian analysis, and the number of Gaussians employed in this case, many alternative solutions could be found that would fit the data as well or perhaps even better. In this case, the operation was repeated several times, and the results presented were selected on the basis of the overall root mean square deviation and on the residuals’ distribution. The UV chromatogram, after rescaling and time shifting, was also nicely decomposed using the same six Gaussians, maintaining the same widths and allowing just a 2% variation in the centers found for the *I*(*t*) *versus t* data (not shown). It was thus possible to back generate a series of *I*(*q*) *versus q* frames with associated sample concentrations for all six G-pks.

**Figure 6:**
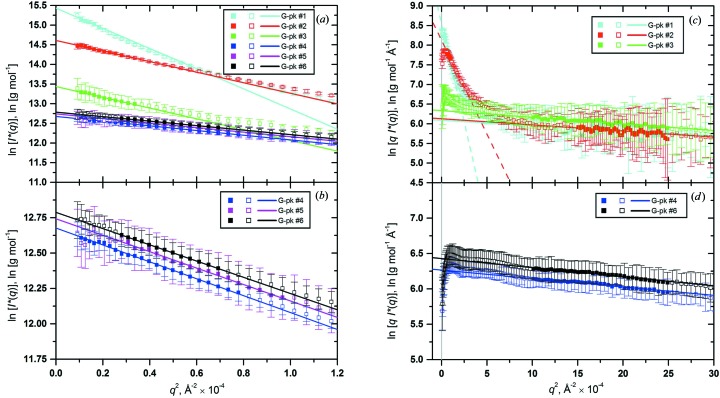
(*a*) ln[I*(*q*)] *versus q* ^2^ Guinier plots of the averaged and concentration/standard-normalized top peak frames for all the six Gaussian peaks derived from the decomposition of the hpHMW-FG SE-HPLC-SAXS data shown in Fig. 5 ▶. The data included in the linear regressions (straight lines) are indicated with filled symbols. All linear regressions were done with SD weighting with automatic rejection of outliers (set at ±2 SD) after definition of an appropriate *q* ^2^ range, limited by the *q*_max_ *R*_g_ < 1.3 rule. (*b*) The Guinier plots for G-pks #4 (blue), #5 (magenta) and #6 (black) are shown on an expanded scale. (*c*), (*d*) Cross-section ln[qI*(*q*)] *versus q* ^2^ Guinier plots for the same data as (*a*) and (*b*) (for the reason of clarity, G-pk #5 has been omitted, only one-half of the actual points are shown for all data sets, and the regression lines were prolonged at unphysical *q* ^2^ < 0 values while the *q* ^2^ = 0 axis is shown as a vertical gray line). Two linear regions were fitted, both limited by the *q*_max_ *R*_c_ < 1 rule, for G-pks #1 and #2 [(*c*), lower *q* ^2^ range, dashed lines; higher *q* ^2^-range, solid lines], and one for all others [solid lines; G-pk #3, (*c*); G-pks #4 and #6, (*d*)].

The top 10–20 frames for each G-pk were then identified and could be normalized by their associated fractional concentration and averaged. All data were subsequently exported into the main *US-SOMO* SAS module for both overall and cross-section Guinier analyses, whose results are shown graphically in Fig. 6 ▶ after conversion of the *I*(*q*) data to I*(*q*) (see §1.3.5 in the supplementary material) and reported numerically in Table 2 ▶. As can be seen in Fig. 6 ▶(*a*), the six Gaussian peaks produced clean data that could easily be analyzed by the overall Guinier method with SD weighting and automatic rejection of outliers (set at ±2 SD) (see Fig. S9) after definition of an appropriate *q* ^2^ range. For G-pks #1–3, the linear range was evident only at very low *q* ^2^ values (limited also by the *q*_max_ *R*_g_ < 1.3 rule). In Fig. 6 ▶(*b*), a blow-up of the intensity range between 11.5 and 14.0 [ln(g mol^−1^)] is presented to allow a better examination of the overall Guinier plots for the G-pks #4 (blue), #5 (magenta) and #6 (black) in which the main chromatographic peak was decomposed. The cross-section Guinier data are presented in Fig. 6 ▶(*c*) and 6 ▶(*d*) (for clarity, the minor G-pk #5 data were omitted from these panels). Considering first the main peak components (Fig. 6 ▶ *d*; G-pks #4, blue, and #6, black), the data show the extended linear range and downturn at very low *q* values expected for a rod-like mol­ecule; the small vertical shift between the two curves indicates a slight difference in the *M*/*L* ratio between the peak components (see Table 2 ▶). Interestingly, all the oligomers curves (Fig. 6 ▶ *c*, G-pks #1–3) show a common linear region with the same slope as and very similar intercepts to the main peak components, but G-pks #1 (cyan) and #2 (red) also display a prominent upturn at very low *q* values that could be independently fitted with a straight line, while G-pk #3 does not.

The numeric results presented in Table 2 ▶ can now be examined. The data and their statistics all appear to be very good to excellent, even at the quite low average concentrations of some of the peaks. Shown in Table 2 ▶ are the results of Guinier analyses on the pre-averaged *I*(*q*) *versus q* data sets, but similar results were obtained by analyzing each frame individually and then making an SD-weighted average of the derived parameters (data not shown). A first apparently odd result is that G-pk #2 and G-pk #3, while having nearly equal [〈*R*_g_^2^〉*_z_*]^1/2^ values, contain material with quite different 〈*M*〉_w_ values, G-pk #2 being close to that of an FG heptamer and G-pk #3, eluting later, being compatible with an FG dimer. A possible explanation for this finding is that G-pk #2 contains FG side-by-side aggregates, while G-pk #3 contains end-to-end covalent dimers, often present in FG preparations. This is nicely confirmed by the cross-section Guinier analyses, which show similar [〈*R*_c_^2^〉*_z_*]^1/2^ and 〈*M*/*L*〉_w__/*z*_ values derived from the intermediate-*q*-range data, probably resulting from the FG main body scattering, and a ∼5–6 times higher value derived from the low-*q*-range data for G-pk #2, indicating the arrangement of the FG aggregates in thicker but loosely bound structures in this sample alone. Thus, notwithstanding a similar overall [〈*R*_g_^2^〉*_z_*]^1/2^, the bulkier aggregates present in G-pk #2 are excluded from the pores in the columns’ packing material more than the slimmer (〈*d*〉 ≃ 50 Å), elongated but flexible end-to-end FG dimers. As for G-pk #1, it is likely to contain a mixture of several types of larger side-by-side FG aggregates. For the material eluting under the main chromatographic peak, our analysis suggests the presence of two distinct but quite similar species (G-pk #5, which was introduced to improve the fitting, is probably an artifact due to the non-pure Gaussian behavior of the eluting material). This is confirmed by the SDS/urea–PAGE analysis of non-reduced samples collected on a separate SE-HPLC run after disconnection from the SAXS setup, presented as an inset in Fig. 3 ▶. The data show how intact FG (top band) is present in all fractions (each one spanning ∼8 SAXS frames) but progressively contaminated by a faster migrating species (lower band) co-eluting with it. The results of the densitometric analyses reported at the bottom of each lane in the Fig. 3 ▶ inset reveal that only a small fraction of intact FG elutes unhindered by the presence of the partially degraded form(s), the two being present in roughly the same amounts in most fractions. Surprisingly, the lower band is practically absent in the injected material (leftmost lane in the Fig. 3 ▶ inset), containing mostly undegraded FG (main band) as well as some covalent aggregates (top band) and traces of heavily degraded species (faint bottom band). This suggests that the new, lower band represents a degradation product forming in-column, either by the action of a contaminating protease or by autolysis, perhaps favored by conformational changes resulting from the gel filtration procedure (the starting material had undergone extensive dialysis in the elution buffer without any noticeable change in composition). From western blots of reduced samples of the same fractions stained with an antibody recognizing the Aα-chain N-terminal end (data not shown), and by comparison with our previous hpHMW-FG analyses (see Figs 1–2 and Table 1 ▶ of Raynal *et al.*, 2013 ▶), this band originates from the degradation of the C-terminal part of the long, mostly unstructured Aα chains. Combining all the densitometric analyses, we could reasonably assign the top band to homo- and hetero-dimers of FG species having the Aα610 and Aα601 chains, and heterodimers of either Aα610 or Aα601 with Aα583 chains (total molecular weights ∼339 000–335 000; average *E* ^280^ = 1.53 ml mg^−1^ cm^−1^ and 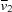 = 0.715 ml g^−1^), and the lower band to heterodimers containing Aα601–Aα461 and Aα583–Aα461 (plus traces of Aα583–Aα424 and Aα461–Aα424), and to Aα583 and Aα461 homodimers (total molecular weights ∼333 000–307 000; average *E* ^280^ = 1.58 ml mg^−1^ cm^−1^ and 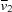 = 0.715 ml g^−1^). Thus, it appears that having one Aα chain cut below residue 461 or two Aα chains cut below residue 583 are the necessary conditions to be part of the lower band, but the reason for this ‘clustering’ of different species in just two bands under non-reducing but denaturing conditions remains to be investigated. At the same time, this analysis reveals that, while a substantial degree of polydispersity is presently unavoidable in FG monomer samples, this problem is much less severe for the material corresponding to the non-reduced gel’s top band. Therefore, the Gaussian decomposition of the main hpHMW-FG HPLC-SAXS peak provides data on nearly full-length FG, freed from oligomers and main degradation products. In any case, the two species are structurally quite similar, as shown by the very close [〈*R*_g_^2^〉*_z_*]^1/2^ and [〈*R*_c_^2^〉*_z_*]^1/2^ values reported in Table 2 ▶ for G-pks #4 and #6. Conformational variability/flexibility coupled to the degradation process could produce a diffusion-controlled statistical partition in and out of the columns’ pores, enhancing the prominent shoulder formation. The appreciable difference in the measured 〈*M*〉_w_ and 〈*M*/*L*〉_w__/*z*_ between G-pks #4 and #6, showing an unexpected higher value in the presence of more degraded material, could be due to preferential interactions between the degraded species, leading to an apparently higher 〈*M*〉_w_. In fact, when single frames of the descending half of G-pk #6 were analyzed individually, a slight concentration dependence of the 〈*M*〉_w_ values was apparent. Extrapolating to *c* = 0 led to the 〈*M*〉_w_ reported in parentheses in Table 2 ▶, significantly lower than the average value and quite close to the expected value based on the Aα chain degradation analysis, notwithstanding its large uncertainty. Importantly, our analysis of the decomposed main peak suggests that the loss of a sizeable amount of material from the C-terminal ends of FG Aα chains does not significantly alter its overall dimension and just slightly alters its cross section. The latter can be rationalized in terms of the molecular arrangements and approximate dimensions of the human FG domains as seen in the crystal structure (Kollman *et al.*, 2009 ▶): a central domain (*R*_c_^2^ ≃ 200 Å^2^, *M* ≃ 38 751 Da), two connecting coiled coils regions (for each, *R*_c_^2^ ≃ 80 Å^2^, *M* ≃ 33 622 Da), two Bβ-chain terminal sub-domains (for each, *R*_c_^2^ ≃ 380 Å^2^, *M* ≃ 44 449 Da), two γ-chain terminal sub-domains (for each, *R*_c_^2^ ≃ 150 Å^2^, *M* ≃ 30 105 Da), plus two Aα-chain C-terminal domains whose structure was not resolved (*M* ≃ 41 906 Da each). Assuming a reasonable *R*_c_^2^ ≃ 300 Å^2^ for the likely largely loosely structured Aα-chain C-terminal domains, a weight average (Hjelm, 1985 ▶) [〈*R*_c_^2^〉_w_]^1/2^ = 15.5 Å results, matching our Table 2 ▶ values. The slightly higher [〈*R*_c_^2^〉*_z_*]^1/2^ and 〈*M*/*L*〉_w__/*z*_ values observed for G-pk #6 could result from the collapse of the degraded remains of the αC regions onto the FG main body.

## 5. Conclusions

The structure of fibrinogen, a protein of relevant biomedical/biotechnological interest, is not fully known. Given the presence of a probably mostly disordered large portion of the Aα chains, multi-resolution studies will be necessary to have a complete three-dimensional picture of FG. An important contribution could come from SAXS data, but aggregation/degradation issues had previously limited their utility. In this article, we have described and applied to this problem the recent developments of an enhanced SAS module of the *US-SOMO* suite, producing much improved data that could be utilized in modeling studies. *US-SOMO* has undergone a (still ongoing) major expansion with the aim of providing a multi-resolution platform for easy combination of scattering data and hydrodynamics results with bead as well as all-atom molecular modeling tools. It has been designed as a hub that allows a variety of operations to be performed, from primary data reduction and analysis to complex modeling approaches. It makes use of several widely used, publicly available software packages from other groups, such as *Gnom*, *Crysol*(*n*) and *FoXS*. It also offers original tools that, to our knowledge, are not yet available elsewhere, such as the mapping over the molecule structure of the relative contributions to a particular distance range in *P*(*r*) *versus r* and, most relevant here, the baseline correction and Gaussian decomposition of SE-HPLC-SAXS data sets. We believe that the latter allows the experimentalist to make the best use of the recorded frames, as illustrated by the reported BSA example and hpHMW-FG application, and may in the future become part and parcel of the SE-HPLC-SAXS data handling and analysis package.

## 6. Related literature

The supplementary material contains a detailed description of the software and references the following additional literature. For details on the Rayleigh structure factors for spheres, see Rayleigh (1911 ▶). The inverse Fourier transform of the *I*(*q*) data to produce a pairwise distance distribution curve is achieved using the indirect transform method (Glatter, 1977 ▶) as implemented in the packages *ATSAS* (Svergun & Koch, 2003) and *Irena* (Ilavsky & Jemian, 2009 ▶), and the Bayesian method described by Hansen (2000 ▶). For the five exponential terms used in the atomic form factors, see Waasmaier & Kirfel (1991 ▶). 

## Supplementary Material

Supplementary material file. DOI: 10.1107/S0021889813027751/kk5149sup1.pdf

## Figures and Tables

**Table 1:** Parameters derived from the Guinier analysis of the BSA SE-HPLC-SAXS data without and with baseline subtraction (bas. sub.) and Gaussian decomposition (G-pk) Values in parentheses are the uncertainties on the least significant digits derived from the linear regression standard deviations.

Frame, G-Pk	*c* (mg ml^−1^)	[〈*R*_g_^2^〉*_z_*]^1/2^ (Å)	〈*M*〉_w_ (g mol^−1^)	*q*_min_ (Å^−1^)	*q*_max_ (Å^−1^)	Fit St. Er.†
Frame #70, original	0.090	46.7 (8)	183300 (2200)	0.0103	0.0282	0.0435
Frame #70, bas. sub.	0.090	41.2 (11)	148300 (2100)	0.0078	0.0282	0.0588
Frame #70, G-pk #1	0.024	50.7 (17)	159800 (3100)	0.0101	0.0282	0.0660
Frame #70, G-pk #2	0.069	38.2 (12)	139400 (1900)	0.0091	0.0282	0.0496
Frame #50, original	0.044	49.2 (45)	235500 (10600)	0.0124	0.0212	0.0768
Frame #50, bas. sub.	0.044	51.1 (23)	180000 (4800)	0.0078	0.0245	0.0975
Frame #50, G-pk #1	0.040	50.6 (25)	194800 (5900)	0.0101	0.0245	0.0898
Frame #81, original	0.173	41.7 (6)	152100 (1400)	0.0144	0.0310	0.0284
Frame #81, bas. sub.	0.173	40.5 (5)	139600 (900)	0.0103	0.0282	0.0231
Frame #81, G-pk #2	0.160	39.7 (5)	141500 (900)	0.0103	0.0282	0.0363
Frame #125, original	1.008	28.1 (3)	77200 (300)	0.0146	0.0315	0.0098
Frame #125, bas. sub.	1.008	27.2 (4)	75300 (300)	0.0124	0.0282	0.0116
Frame #125, G-pk #3	0.982	27.2 (4)	77300 (300)	0.0124	0.0282	0.0116

†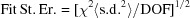, where s.d. are the standard deviations associated with each data point and DOF is the degrees of freedom of the linear fit. This provides a goodness-of-fit value invariant with the magnitude of the standard deviations.

**Table 2:** Parameters derived from the Guinier analyses of the hpHMW-FG SE-HPLC-SAXS data with baseline subtraction and Gaussian decomposition Values in parentheses are the uncertainties on the least significant digits derived from the linear regression standard deviations.

G-pk [frames averaged]	*c *(average) (mg ml^−1^)	[〈*R*_g_^2^〉*_z_*]^1/2^ (Å)	〈*M*〉_w_ (kg mol^−1^)	*q*_min_ (Å^−1)^	*q*_max_ (Å^−1^)	Points used	Fit St. Er. (average)	[〈*R*_c_^2^〉*_z_*]^1/2^ (Å)	〈*M*/*L*〉_w/*z*_ (g mol^−1^ Å^−1^)	*q*_min_ (Å^−1)^	*q*_max_ (Å^−1^)	Points used	Fit St. Er. (average)
1 [73–85]	0.043	278.2 (131)	5040 (18)	0.0033	0.0043	5	0.0138	141.6 (23)	5105 (56)	0.0045	0.0068	10	0.0078
								17.1 (20)	446 (27)	0.0350	0.0499	57	0.1056
2 [81–95]	0.091	200.2 (19)	2205 (18)	0.0040	0.0063	10	0.0065	97.9 (10)	3376 (26)	0.0078	0.0101	10	0.0036
								17.9 (15)	462 (23)	0.0388	0.0499	45	0.0516
3 [111–130]	0.101	202.6 (36)	685.6 (83)	0.0033	0.0058	11	0.0126	18.4 (04)	565 (7)	0.0318	0.0499	73	0.0321
4 [159–174]	0.587	133.8 (9)	320.3 (13)	0.0033	0.0088	23	0.0082	15.5 (2)	528 (3)	0.0318	0.0499	73	0.0120
5 [173–183]	0.166	132.1 (13)	342.0 (26)	0.0056	0.0098	18	0.0100	15.5 (4)	576 (6)	0.0318	0.0499	73	0.0229
6 [185–200]	0.537	131.2 (8)	358.0 (12)	0.0053	0.0083	13	0.0031	16.1 (2)	620 (4)	0.0318	0.0499	73	0.0116
			[311 (30)]†										

† From an 〈*M*〉_w_ *versus c* linear fit of frames 193–199.

## Footnotes

1 Supplementary material is available from the IUCr electronic archives (Reference: KK5149). Services for accessing this material are described at the back of the journal.

2 The data presented here, as well as the BSA patterns, were recorded during a measuring session in 2010. Since then, the accumulation of material leading to the capillary fouling problem has been experimentally addressed. A cleaning device has been designed and installed on the SWING beamline (P. Roblin & J. Pérez, Synchrotron Soleil), so that the measuring cell goes through one or several cleaning cycles involving water flushing, detergent and again water flushing before elution buffer flows again through the cell at the end of each SE-HPLC-SAXS run. This and careful tuning of X-ray exposure time has significantly improved the situation, without completely eliminating capillary fouling in all cases. To date, fouling is not as serious an issue as shown here, and the *I*(*t*) *versus t* plot and baseline evaluation provides an objective assessment of data quality.

## References

[bb101] Aster, R. C., Brochers, B. & Thurber, C. H. (2005). *Parameter Estimation and Inverse Problems.* Burlington, San Diego, London: Elsevier Academic Press.

[bb1] Blombäck, B. (1996). *Thromb. Res.* **83**, 1–75. 10.1016/0049-3848(96)00111-9 8837305

[bb2] Boccaccio, C. & Medico, E. (2006). *Cell. Mol. Life Sci.* **63**, 1024–1027. 10.1007/s00018-005-5570-9 PMC11135989 16612563

[bb3] Brookes, E., Demeler, B. & Rocco, M. (2010). *Macromol. Biosci.* **10**, 746–753. 10.1002/mabi.200900474 20480513

[bb4] Brookes, E., Demeler, B., Rosano, C. & Rocco, M. (2010). *Eur. Biophys. J.* **39**, 423–435. 10.1007/s00249-009-0418-0 PMC2872189 19234696

[bb5] Brookes, E., Singh, R., Pierce, M., Marru, S., Demeler, B. & Rocco, M. (2012). *XSEDE ’12. Proceedings of the 1st Conference of the Extreme Science and Engineering Discovery Environment: Bridging from the eXtreme to the Campus and Beyond*. New York ACM. 10.1145/2335755.2335839.

[bb6] Bujacz, A. (2012). *Acta Cryst.* D**68**, 1278–1289. 10.1107/S0907444912027047 22993082

[bb7] Burley, S. K., Almo, S. C., Bonanno, J. B., Capel, M., Chance, M. R., Gaasterland, T., Lin, D., Sali, A., Studier, F. W. & Swaminathan, S. (1999). *Nat. Genet.* **23**, 151–157. 10.1038/13783 10508510

[bb8] Byron, O. (1997). *Biophys. J.* **72**, 408–415. 10.1016/S0006-3495(97)78681-8 PMC1184331 8994627

[bb9] Byron, O. (2000). *Methods Enzymol.* **321**, 278–304. 10.1016/s0076-6879(00)21199-3 10909063

[bb10] Cardinali, B., Profumo, A., Aprile, A., Byron, O., Morris, G., Harding, S. E., Stafford, W. F. & Rocco, M. (2010). *Arch. Biochem. Biophys.* **493**, 157–168. 10.1016/j.abb.2009.10.008 19853574

[bb11] David, G. & Pérez, J. (2009). *J. Appl. Cryst.* **42**, 892–900.

[bb12] Ding, F. & Dokholyan, N. V. (2006). *PLoS Comput. Biol.* **2**, 0725–0733. 10.1371/journal.pcbi.0020085 PMC1487181 16839198

[bb13] Dokholyan, N. V., Buldyrev, S. V., Stanley, H. E. & Shakhnovich, E. I. (1998). *Fold. Des.* **3**, 577–587. 10.1016/S1359-0278(98)00072-8 9889167

[bb14] Fasano, M., Curry, S., Terreno, E., Galliano, M., Fanali, G., Narciso, P., Notari, S. & Ascenzi, P. (2005). *IUBMB Life*, **57**, 787–796. 10.1080/15216540500404093 16393781

[bb102] Glatter, O. (1977). *J. Appl. Cryst.* **10**, 415–421.

[bb15] Glatter, O. & Kratky, O. (1982). *Small-Angle X-ray Scattering.* New York: Academic Press.

[bb103] Hansen, S. (2000). *J. Appl. Cryst.* **33**, 1415–1421.

[bb16] Heel, M. van, Gowen, B., Matadeen, R., Orlova, E. V., Finn, R., Pape, T., Cohen, D., Stark, H., Schmidt, R., Schatz, M. & Patwardhan, A. (2000). *Q. Rev. Biophys.* **33**, 307–369. 10.1017/s0033583500003644 11233408

[bb17] Hjelm, R. P. (1985). *J. Appl. Cryst.* **18**, 452–460.

[bb104] Ilavsky, J. & Jemian, P. R. (2009). *J. Appl. Cryst.* **42**, 347–353.

[bb18] Kollman, J. M., Pandi, L., Sawaya, M. R., Riley, M. & Doolittle, R. F. (2009). *Biochemistry*, **48**, 3877–3886. 10.1021/bi802205g 19296670

[bb105] Lawson, C. L. & Hanson, R. J. (1995). *Solving Least Squares Problems* Philadelphia: SIAM.

[bb19] Litvinov, R. I., Yakovlev, S., Tsurupa, G., Gorkun, O. V., Medved, L. & Weisel, J. W. (2007). *Biochemistry*, **46**, 9133–9142. 10.1021/bi700944j PMC2678904 17630702

[bb20] Mathew, E., Mirza, A. & Menhart, N. (2004). *J. Synchrotron Rad.* **11**, 314–318. 10.1107/S0909049504014086 15211037

[bb21] McEwen, B. F. & Marko, M. (2001). *J. Histochem. Cytochem.* **49**, 553–564. 10.1177/002215540104900502 11304793

[bb22] Mertens, H. D. & Svergun, D. I. (2010). *J. Struct. Biol.* **172**, 128–141. 10.1016/j.jsb.2010.06.012 20558299

[bb23] Mosesson, M. W. (1983). *Molecular Biology of Fibrinogen and Fibrin: Fibrin Heterogeneity*, edited by M. W. Mosesson & R. F. Doolittle, pp. 97–113. New York: Annals of the New York Academy of Science.

[bb24] Pérez, J. & Nishino, Y. (2012). *Curr. Opin. Struct. Biol.* **22**, 670–678. 10.1016/j.sbi.2012.07.014 22954648

[bb25] Peters, T. (1985). *Adv. Protein Chem.* **37**, 161–245. 10.1016/s0065-3233(08)60065-0 3904348

[bb26] Petoukhov, M. V., Franke, D., Shkumatov, A. V., Tria, G., Kikhney, A. G., Gajda, M., Gorba, C., Mertens, H. D. T., Konarev, P. V. & Svergun, D. I. (2012). *J. Appl. Cryst.* **45**, 342–350. 10.1107/S0021889812007662 PMC4233345 25484842

[bb27] Petoukhov, M. V. & Svergun, D. I. (2005). *Biophys. J.* **89**, 1237–1250. 10.1529/biophysj.105.064154 PMC1366608 15923225

[bb28] Poitevin, F., Orland, H., Doniach, S., Koehl, P. & Delarue, M. (2011). *Nucleic Acids Res.* **39**, W184–W189. 10.1093/nar/gkr430 PMC3125794 21665925

[bb29] Putnam, C. D., Hammel, M., Hura, G. L. & Tainer, J. A. (2007). *Q. Rev. Biophys.* **40**, 191–285. 10.1017/S0033583507004635 18078545

[bb30] Rai, N., Nöllmann, M., Spotorno, B., Tassara, G., Byron, O. & Rocco, M. (2005). *Structure*, **13**, 723–734. 10.1016/j.str.2005.02.012 15893663

[bb106] Rayleigh (1911). *Proc. R. Soc. London Ser. A*, **84**, 25–46.

[bb31] Raynal, B., Cardinali, B., Grimbergen, J., Profumo, A., Lord, S. T., England, P. & Rocco, M. (2013). *Thromb. Res.* **132**, e48–e53. 10.1016/j.thromres.2013.04.005 PMC3742573 23642654

[bb32] Sayle, R. A. & Milner-White, E. J. (1995). *Trends Biochem. Sci.* **20**, 374–376. 10.1016/s0968-0004(00)89080-5 7482707

[bb33] Schneidman-Duhovny, D., Hammel, M. & Sali, A. (2010). *Nucleic Acids Res.* **38**, W540–W544. 10.1093/nar/gkq461 PMC2896111 20507903

[bb34] Smith, J. D., Clayton, D. A., Fields, S., Hellinga, H. W., Kuriyan, J., Levitt, M., Peishoff, C. E., Rosen, M. & Taylor, S. S. (2007). *Report of the Protein Structure Initiative Assessment Panel*, http://www.nigms.nih.gov/News/Reports/PSIAssessmentPanel2007.htm.

[bb35] Spotorno, B., Piccinini, L., Tassara, G., Ruggiero, C., Nardini, M., Molina, F. & Rocco, M. (1997). *Eur. Biophys. J.* **25**, 373–384.

[bb107] Stuhrmann, H. B. (1970). *Acta Cryst.* A**26**, 297–306.

[bb108] Stuhrmann, H. B., Koch, M. H., Parfait, R., Haas, J., Ibel, K. & Crichton, R. R. (1977). *Proc. Natl Acad. Sci. USA*, **74**, 2316–2320. 10.1073/pnas.74.6.2316 PMC432161 329279

[bb36] Suhre, K., Navaza, J. & Sanejouand, Y.-H. (2006). *Acta Cryst.* D**62**, 1098–1100. 10.1107/S090744490602244X 16929111

[bb37] Svergun, D., Barberato, C. & Koch, M. H. J. (1995). *J. Appl. Cryst.* **28**, 768–773.

[bb38] Svergun, D. I. & Koch, M. H. J. (2003). *Rep. Prog. Phys.* **66**, 1735–1782.

[bb39] Svergun, D. I., Richard, S., Koch, M. H., Sayers, Z., Kuprin, S. & Zaccai, G. (1998). *Proc. Natl Acad. Sci. USA*, **95**, 2267–2272. 10.1073/pnas.95.5.2267 PMC19315 9482874

[bb40] Svergun, D. I. & Stuhrmann, H. B. (1991). *Acta Cryst.* A**47**, 736–744.

[bb41] Todd, A. E., Marsden, R. L., Thornton, J. M. & Orengo, C. A. (2005). *J. Mol. Biol.* **348**, 1235–1260. 10.1016/j.jmb.2005.03.037 15854658

[bb42] Topf, M., Lasker, K., Webb, B., Wolfson, H., Chiu, W. & Sali, A. (2008). *Structure*, **16**, 295–307. 10.1016/j.str.2007.11.016 PMC2409374 18275820

[bb43] Tsurupa, G., Hantgan, R. R., Burton, R. A., Pechik, I., Tjandra, N. & Medved, L. (2009). *Biochemistry*, **48**, 12191–12201. 10.1021/bi901640e PMC2812052 19928926

[bb109] Waasmaier, D. & Kirfel, A. (1995). *Acta Cryst.* A**51**, 416–431.

[bb44] Weisel, J. W. (2005). *Adv. Protein Chem.* **70**, 247–299. 10.1016/S0065-3233(05)70008-5 15837518

[bb45] Williamson, T. E., Craig, B. A., Kondrashkina, E., Bailey-Kellogg, C. & Friedman, A. M. (2008). *Biophys. J.* **94**, 4906–4923. 10.1529/biophysj.107.113167 PMC2397332 18212017

[bb46] Wriggers, W., Milligan, R. A. & McCammon, J. A. (1999). *J. Struct. Biol.* **125**, 185–195. 10.1006/jsbi.1998.4080 10222274

[bb47] Yang, Z., Kollman, J. M., Pandi, L. & Doolittle, R. F. (2001). *Biochemistry*, **40**, 12515–12523. 10.1021/bi011394p 11601975

