# Supplementary material for: Fibrinogen species as resolved by HPLC-SAXS data processing within the UltraScan Solution Modeler (US-SOMO) enhanced SAS module
Source: J Appl Crystallogr. 2013 Nov 15;46(Pt 6):1823–33. doi: 10.1107/S0021889813027751 (PMC3831300; doi:10.1107/S0021889813027751)
Supplement: Supplementary file 1 [file j-46-01823-sup1.pdf]

Fibrinogen species as resolved by HPLC-SAXS data processing within the UltraScan Solution Modeler (US-SOMO) enhanced SAS module

Supplementary Material.

1. US-SOMO SAS module.

1.1. Main Panel.

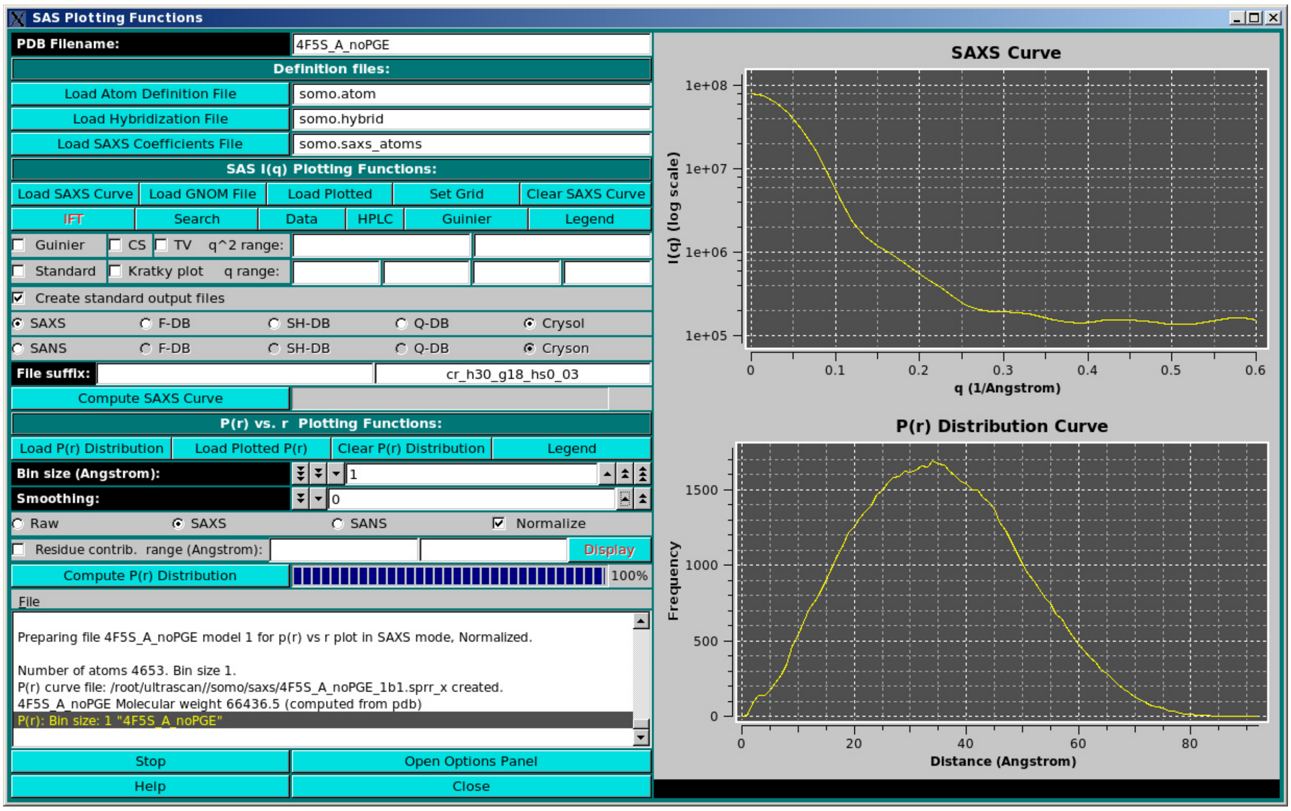

Figure S1 Panel A, the renewed GUI of the US-SOMO SAS module main panel. In the graphics windows, the *I(q) vs q* and the *P(r) vs. r* curves, both computed directly from the BSA crystal structure 4F5S (Bujacz, 2012), using Crysol and the US-SOMO internal P(r)/SAXS method, respectively, are shown.

The new GUI of the US-SOMO SAS module is shown in Fig. S1. It is divided in two halves, the top dealing with reciprocal-space operations, and the bottom for real-space operations. Each half can be hidden by clicking on its bar, expanding the other half as a consequence. If a PDB file was previously uploaded in the US-SOMO main panel, its name will appear in the first field at the top, and all subsequent calculations will be done on it. The three buttons/fields below the first field give the user the possibility to upload reference files different from the default ones (automatically uploaded and shown in the corresponding fields), containing the atoms properties, hybridizations, and SAXS coefficients, respectively, that will be used in the computations (see Brookes *et al.*, 2010a; Brookes *et al.*, 2010b). This part of the panel is common for both SAXS/SANS *I(q) vs. q* and *P(r) vs. r* plotting/computation functions. The first series of buttons in the “SAS

I(q) Plotting Functions” section deal with operations on previously computed or experimental data. Loading is done with the “Load SAXS (SANS)" button (the SAXS/SANS label will switch depending on whether SAXS or SANS operations have been chosen, see below). If one or more curves are already present in the graphics window, rescaling of the new entry is possible by selecting the target curve in a pop-up window. Besides our own data formats, including multiple datasets in a comma-separated variable (csv) format (see below), the Crysol/Cryson format (Svergun *et al.*, 1995; Svergun *et al.*, 1998) is also recognized. “Load GNOM File” will allow the uploading of files produced by the GNOM program of the ATSAS suite (Petoukhov et al., 2012). In that case, the corresponding *P(r) vs. r* curve will also be shown in the bottom graphics window. “Load Plotted” will instead open a new window allowing several operations to be performed on the data which are currently displayed in the graphics window. “Set Grid” will open a dialog box allowing to set the starting and ending *q* values, and the *q*-interval, taken from any of the plotted curves. This is quite useful when computing a SAXS/SANS *I(q) vs. q* curve from a structure to be compared with experimental data. “Clear SAXS (SANS) Curve” will instead sequentially remove the data from the graphics window. “Legend” will display the names of the files plotted and their associated line colours. “IFT” (not available in the current release) will perform an inverse Fourier transform of the *I(q)* data to produce a pair-wise distance distribution curve using the indirect transform method (Glatter, 1977) as implemented in the packages ATSAS (Svergun & Koch, 2003) and/or Irena (Ilavsky and Jemian, 2009), and/or the Bayesian method described by Hansen (Hansen, 2000). Final implementation choices will be determined by a comparative analysis of these methods. “Search” will open up a dialog box where buffer electron density, excluded volume scaling, and the excluded volume of explict waters can be automatically varied and and fit against experimental data. “Data” will open a module containing various utilities for primary and processed data treatment, with the ability to perform buffer subtractions, normalization and curve joining. Although functional, this module is still under development and will be fully described in a future publication. “HPLC” will call a novel utility dedicated to the advanced processing of HPLC-SAXS data, for instance allowing to plot the scattered intensity at each momentum transfer *q* as a function of the elution profile, tracing and subtracting baselines, singular value decomposition (SVD), and Gaussian decomposition of not resolved peaks (see **1.4** in this Supplement). Finally, the “Guinier” button will allow the manual or semi-automated computation of the global, cross-section (CS, for rod-like molecules), and transverse (TV for disk-like molecules) *z*-average radii of gyration, $<R_g^2>_z$, $<R_c^2>_z$, and $<R_t^2>_z$, respectively, and of the corresponding weight-average intensities at zero scattering angle $<I(0)>_w$ from Guinier plots (Glatter & Kratky, 1982). The “Guinier Options” panel (see **1.3.5** in this Supplement) will open up and allow modifying the settings, and then the alternative Guinier calculations can be launched from separate buttons. The Guinier plots $ln\ I(q)$ *vs.* q^2, $ln[q*I(q)]$ *vs.* q^2 (CS), and $ln[q^2*I(q)]$ *vs.* q^2 (TV) can be shown by clicking on their checkboxes, and their display q^2 ranges can be modified by entering values in the corresponding fields. Computations of the weight-average molecular weight $<M>_w$, mass-length ratio $<M/L>_{w/z}$, and mass/area ratio $<M/A>_{w/z}$ from corresponding $<I(0)>_w$ values are done using constants and parameters present in the “Guinier Options” panel. It is also possible to plot the data as a Kratky plot ($q^2*I(q)$ *vs. q*; Glatter & Kratky, 1982) by selecting the dedicated checkbox, and entering *q* ranges in the corresponding fields. When comparing experimental and calculated data, a fit can be done in either standard or Kratky mode, with or without standard deviation

(SD) weighting, by selecting the appropriate options in the Miscellaneous Options panel of the SAS Options menu (see **1.3.6** in this Supplement). Clicking on "Standard" will revert the plot to the *I(q) vs. q* mode. All graphs can be zoomed in by selecting an area with the mouse keeping pressed the left-side button.

The method used for the computations of *I(q) vs. q* from atomic coordinates can be selected in the bar below the "Create standard output files" checkbox. SAXS or SANS is the first option (default: SAXS; if SANS is selected, the name of the plot in the right-side upper panel will change accordingly). For SAXS, the choice is between full Debye ("F-DB"), Debye with spherical harmonics ("SH-DB"), a fast methods based on FoXS (Schneidman-Duhovny *et al.*, 2010), whose code is only available for Linux operating systems ("quick" Debye, "Q-DB"), and Crysol (Svergun *et al.*, 1995); the latter needs to be downloaded (http://www.embl-hamburg.de/biosaxs/software.html) and pre-installed in the existing ultrascan/bin program directory. For SANS, only Cryson (Svergun *et al.*, 1998), which again needs to be downloaded and pre-installed as above, is currently available; the corresponding SANS implementations of full Debye, Debye with spherical harmonics, and quick Debye will be made available in a future release. For SAXS, the full Debye method (Glatter & Kratky, 1982) first calculates the contrast atomic form factors f'_{ij} for each atom *i* at each scattering vector q_j:

$$f'_{j,i} = c^{(i)} + \sum_{k=1}^{L} a_k^{(i)} e^{-b_k^{(i)}\left(\frac{q_j}{4\pi}\right)^2} - v_{ex}\rho_0\, e^{-q_j^2 \frac{v_{ex}^{2/3}}{4\pi}} \qquad \text{(S1)}$$

where $a_k^{(i)}$, $b_k^{(i)}$, and $c^{(i)}$ are the pre-exponential terms, the exponential terms, and the constant term, respectively, of the atomic form factors taken from the International Tables for Crystallography and stored in the *somo.SAXS_atom* file, *L* is the number of exponential terms used (4 or 5, user-selectable; for the latter, see Waasmaier & Kirfel, 1991), v_{ex} is the excluded volume of each atom retrieved from the *somo.atom* file, and ρ_0 is the solvent electron density, stored in the US-SOMO SAXS calculation options (default: water, 0.334 e/Å^3). If implicit hydrogens are used, as is typical for most structures derived from X-ray crystallography studies, then structure factors computed from a Debye computation utilizing typical hydrogen positions replace the atomic form factors. The v_{ex} for non-H atoms stored in the *somo.atom* file already includes the H atoms bound to every particular group defined there (*e.g.*, C_4H_3 v_{ex} = 31.89 Å^3, C_4H_0 v_{ex} = 16.44 Å^3). The I_j are then computed for each q_j as:

$$I_j = 2\sum_{i=1}^{n-1}\sum_{k=i+1}^{n}\left[f'_{j,i}\, f'_{j,k}\,\frac{\sin(q_j\, r_{i,k})}{q_j\, r_{i,k}}\right] + \sum_{i=1}^{n}\left(f'_{j,i}\right)^2 \qquad \text{(S2)}$$

where the $r_{i,k}$ are is the distance between the ith and k^{th} scattering centres.

The spherical harmonics method is based on a spherical Bessel function expansion of the Debye equation and is described further in (Stuhrmann, 1970; Stuhrmann *et al.*, 1977; Svergun *et al.*, 1991).

When computing a SAXS *I(q) vs. q* curve the original PDB structure filename is appended with a code identifying the computation method used (*e.g.,* "db" for full Debye) as appears in the right-side field, and the extension is ".ssaxs" (".ssans" for SANS files). If SH-DB or Crysol/Cryson are used, the extension will also report the used values of user-selectable parameters (in the example in Fig. 1, *h30* is the maximal order of the spherical harmonics, *g18* is the Fibonacci grid used, and *hs0_03* is the contrast of the hydration shell) that can be set in the SAXS Computation Options and SANS Computation options panels of the SAS Options menu (see **1.3.1** and **1.3.2** in this Supplement). A user-definable suffix can also be entered in the left-side field. Computation of an *I(q) vs. q* curve is started by pressing the "Compute SAXS (SANS) Curve" button, with progress reported as a bar and a % number to its right.

In the "P(r) vs. r Plotting Functions" section, a previously-generated or experimentally-derived *P(r) vs. r* curve can be uploaded in the graphics window by pressing the "Load P(r) Distribution" button, and the graphics window can be cleared by pressing the "Clear P(r) Distribution" button. As for the *I(q) vs. q* window, "Load Plotted" will open up open a new window allowing several operations to be performed on the data which were currently showed in the graphics window (see **1.2.1** in this Supplement), and "Legend" will display the names of the files plotted and their associated line colours. For the *P(r) vs. r* computation from an atomic-level structure or a bead model, two fields control the bin size and a smoothing window (optional), respectively. The equation used is:

$$P(r) = \frac{\sum_i \sum_j \left\{ (b_i - b_{0i}) * (b_j - b_{0j}) * \delta(r - r_{ij}) \right\}}{< b - b_0 >^2} \tag{S3}$$

where b_i and b_j, set to 1 for the "raw" computations, are the number of electrons of the *i* and *j* atomic groups for the SAXS computations, and are the neutron scattering lengths of the *i* and *j* atomic groups at a set D_2O fraction *Y* (see below) for the SANS computations. All these values are stored in the US-SOMO reference tables, and the D_2O fraction *Y* can be set in the SANS computation options panel of the SAS Options menu. The Kronecker's delta $\delta(r - r_{ij})$ is applied to the distances r_{ij} between the atom's *i* and *j* centres for every bin *r*. The terms b_{0i} and b_{0j} account for the solvent scattering density. For SAXS, $b_{0i} = 10 * (R_i / R_{H2O})^3$ where 10 is the number of electrons in a water molecule, R_i is the van der Waals (vdW) radius of the ith atom (from the *somo.residue* table) and R_{H2O} is the vdW radius of a bulk water molecule (as set in the SAXS options). By pressing the "Compute P(r) distribution" button a 3-column file is created, containing the bins *r*, the non-normalized *P(r)*, and the normalized (defined below) *P(r)* values, respectively. Files will have the extension *.sprr_r*, *.sprr_x* , and *.sprr_n* for the "Raw", SAXS and SANS settings, respectively. In addition, a suffix containing the bin size used (*e.g., b1* for bin size = 1) will be added at the end of the filename. Normalization is automatically done by first calculating the area under the *P(r)* curve and then dividing it by the molecular weight of the structure (or bead model), which is computed from the sequence or can be entered in a pop-up panel and later stored in the file. Each *P(r)* value is then divided by this ratio. The "Normalize" checkbox when selected will display the normalized curve in the graphics window on the right side, with automatic rescaling upon adding a new graph. Before starting a new *P(r) vs. r* computation, the residues contributing to

a particular bin can be identified by selecting the “Residue contr. range (Angstrom)” checkbox and entering a range (min-max) in the two fields at its right. In this case, when the *P(r) vs. r* computation is completed, the “Display” button becomes available, and pressing it will call RasMol (Sayle & Milner-White, 1995), which is the molecular visualization software installed with US-SOMO. There the structure will be visualized with the residues contributing to the selected range colour-coded from yellow (max contribution) to blue (min contribution). The other residues will be coloured gray. New ranges can be entered and the results shown without having to re-do the *P(r) vs. r* computations. The graphics window will show every new *P(r) vs. r* curve in a different colour, without erasing curves already present. The correspondence between the colours and the files is reported in the progress window (the same happens also for the *I(q) vs. q* operations), and can be also toggled on and off below the graphics window by pressing the “Legend” button. Progress in the operations is reported in the advancement bar on the side of the “Compute P(r) distribution” button, and in the progress window below the buttons. “Stop” will halt the current operation, and the option panel containing the settings controlling the operations, such as the D_2O fraction, can be directly accessed by pressing the “Open Options Panel” button.

1.2. Main Panel – Additional features.

1.2.1. Load CSV-style SAS Results module.

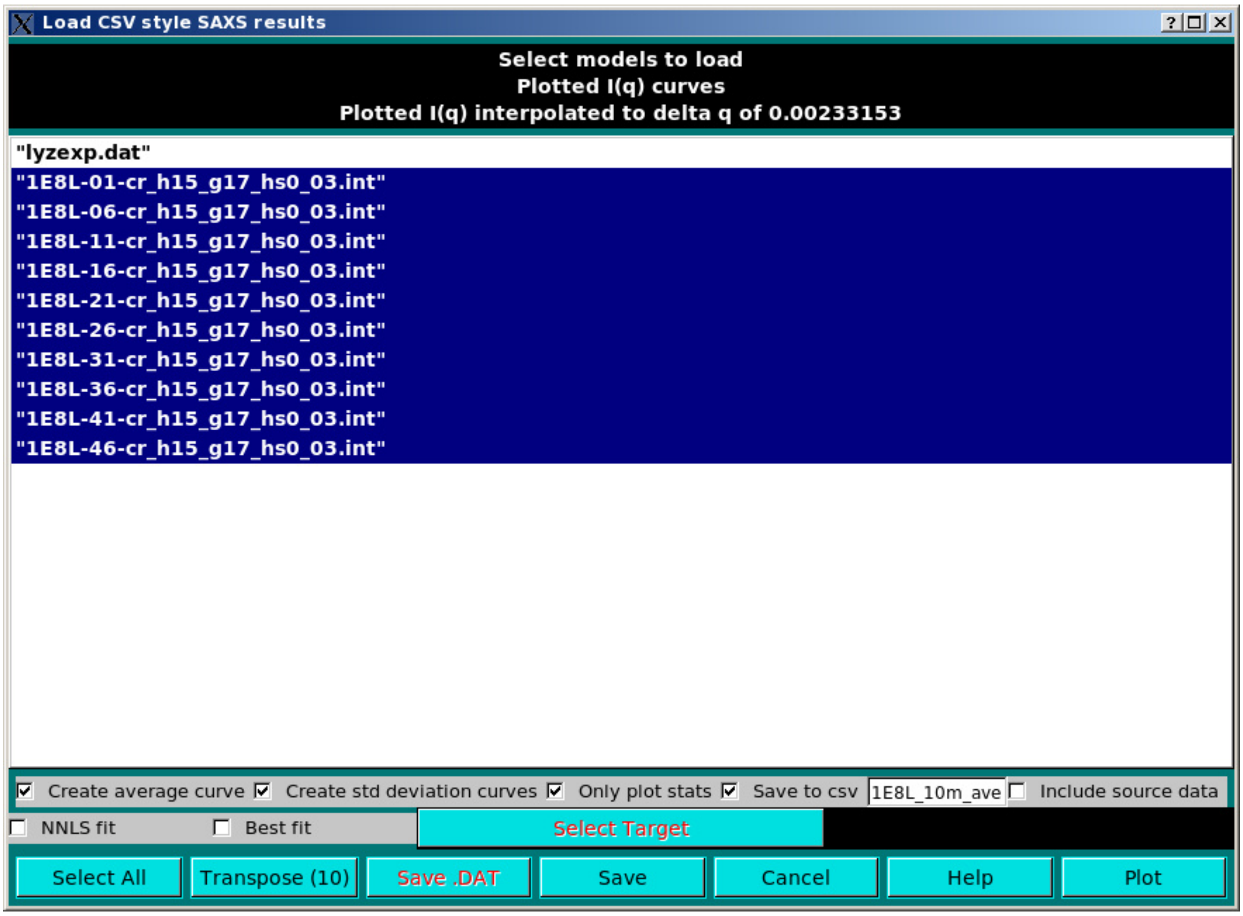

Figure S2 The load csv-style SAXS results window. Shown are 10 models (highlighted) and one experimental curve.

Previously computed csv-style *I(q) vs. q* files, such as those generated using the US-SOMO Batch Mode (Brookes *et al.*, 2010a; Brookes *et al.*, 2010b), can also be uploaded in the SOMO SAXS/SANS graphics window. In this case, a new window will open listing all the individual curves present in the csv file, plus all other files already uploaded into the graphics window. The "Load Plotted" button will also open the csv-style SAXS results window. Individual data can be selected/deselected by clicking on them, or they can all be selected/deselected by pressing the Select All button. Several operations can then be performed before returning to the graphics window. In the example shown in Fig. S2, ten datasets were selected and the "Create average curve", "Create std deviation curves", "Only plot stats", and "Save to csv" checkboxes were ticked. When pressing "Plot", this will create an average curve from the selected dataset, plus average +SD and average -SD curves. In this example, only the average and average ±SD curves will be plotted in the graphics window, because the "Only plot stats" checkbox was selected. Furthermore, the resulting average and average ±SD curves will be saved in another csv file, whose filename is entered in the field next to the "Save to csv" checkbox. The original source data can be also saved in the new csv file by ticking the "Include source data" checkbox.

Other operations are available within this module. The selected data, which are stored in rows, can be transposed into columns by pressing the "Transpose" button (the number in parentheses in the button label will show how many datasets are going to be transposed). This will bring up a save window where a path and a filename can be chosen. The transposed file will be saved in csv format. Alternatively, a Crysol-compatible file can be saved by pressing the "Save .DAT" button, bringing up again a save window where a path and a filename can be chosen. Pressing the "Save" button will instead save the selected dataset(s) into another csv file maintaining the rows storage format. Pressing "Cancel" will close this window.

More operations can be performed on multiple *I(q) vs. q* files within this module. A non-negative least squares (NNLS) procedure can be utilized to find the best combination of model curves matching an experimental dataset. This operation is performed by first selecting the experimental dataset by clicking on it, and then pressing the "Select Target" button (available only when a single dataset is selected). The chosen dataset name will then appear in the field next to the "Select Target" button. If a single (experimental) data file was already loaded into the graphics window, it will be automatically chosen as the target dataset (but it could be changed as described above). Next, the datasets on which the NNLS is to be performed are selected (efficiently by first pressing the "Select All" button and then de-selecting the unwanted datasets by single-clicking on their names). Pressing the "Plot" button will then launch the NNLS operation, at the end of which the program will return to the main US-SOMO SAXS/SANS panel. A pop-up window asking to confirm/change/deselect a target curve upon which the new data to be plotted can be rescaled will also appear. After the appropriate selection, the graphics window (Fig. S3, top) and the progress window in the bottom-left corner of the SAXS/SANS panel (Fig. S1) will be updated. The latter will show the fitting statistics (for instance the χ^2 value) and list the % contribution of each file to the composite curve fitting the target curve. A residuals window will pop-up, as shown is Fig. S3, bottom. In the "Plot difference" mode, the

two horizontal bars indicate ±2 SD from target. If “Plot percent” is selected, the bars will indicate 5% deviation from target. A log scale mode is also available.

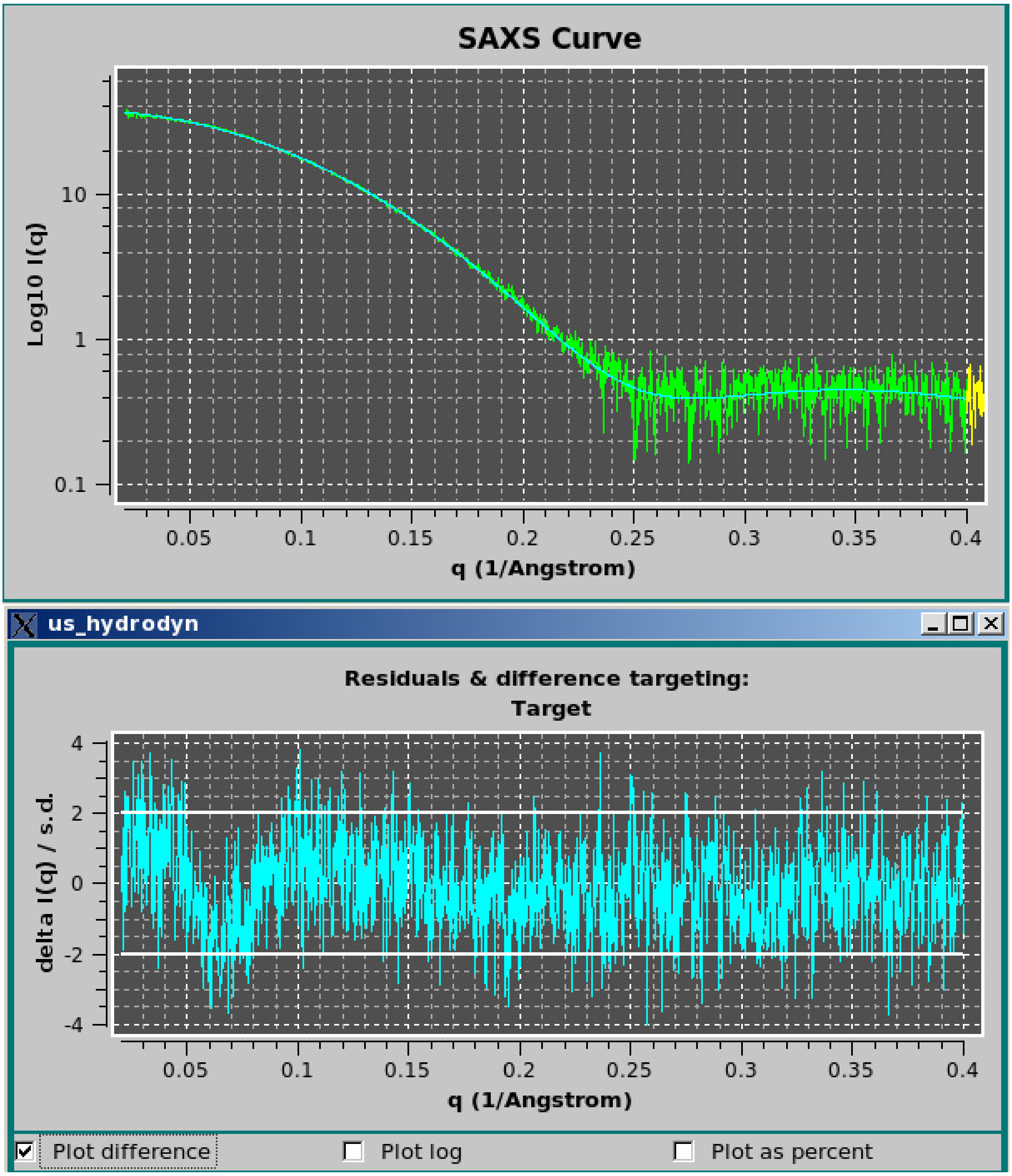

Figure S3 Top: the updated SAXS graphics panel showing an experimental curve (green) and the NNLS-derived best fitting curve obtained by a combination of the curves computed from ten structures (cyan). Bottom: the residuals window.

Likewise, the best matching curve to a target curve from an ensemble of curves can be found by selecting the “Best fit” checkbox, and proceed as described above for the “NNLS fit”.

1.3. SAS Options Panel.

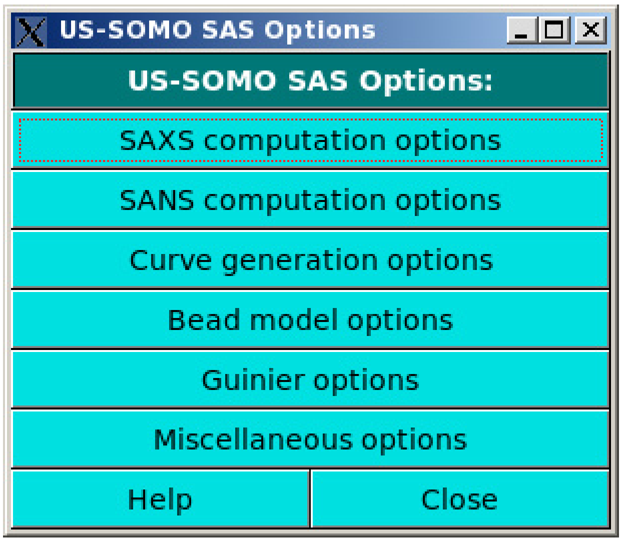

Figure S4 The US-SOMO SAS Option panel listing the available modules.

Pressing the "Open Option Panel" button will bring up the window shown in Fig. S4, from which all the listed submenus can be reached.

1.3.1. SAXS Computation Options module.

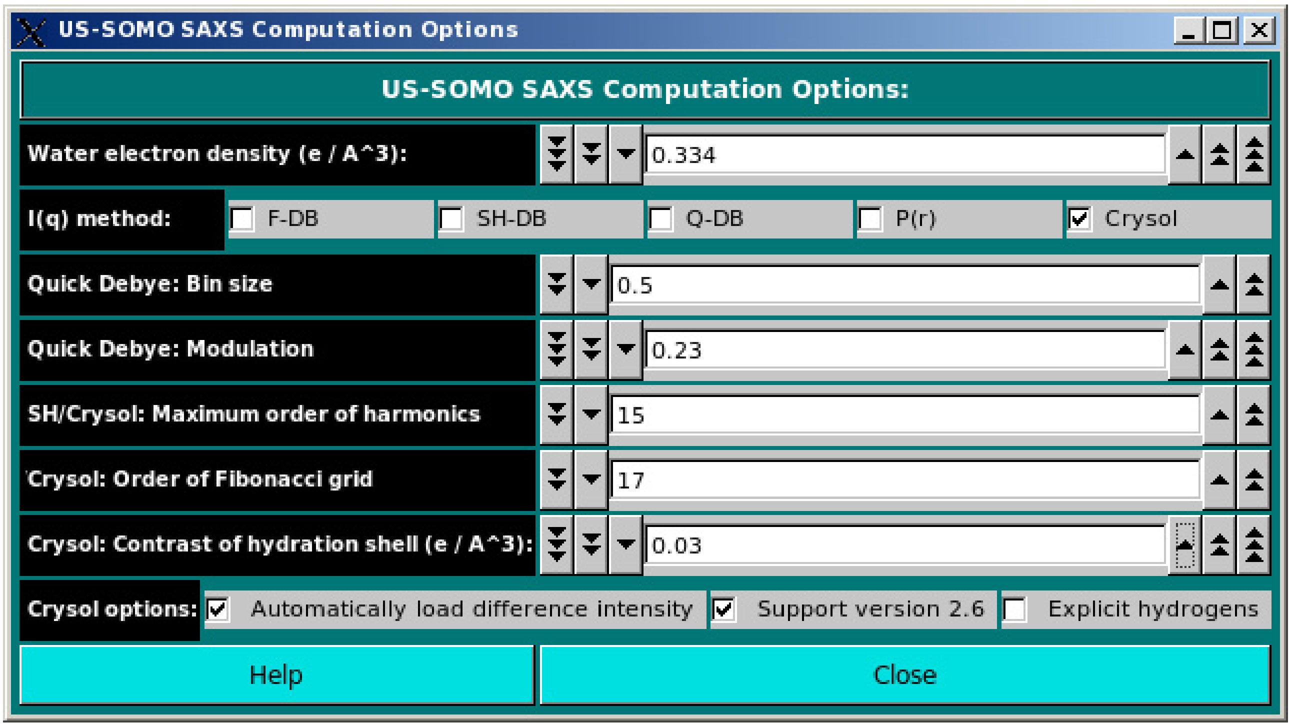

Figure S5 The US-SOMO SAXS computation options module.

In this module are set the options and parameters for the computations of *I(q) vs. q* curves from atomic-level structures. The solvent (here taken as water) electron density is entered in the first field. Next comes the choice of the computational method used, as already described. For the quick-Debye (Q-DB) method a *P(r)* function can also be computed by ticking its checkbox, and it also requires values for the bin size and the modulation as defined for the FoXS method (Schneidman-Duhovny *et al.*, 2010). Next follow the parameters utilized by Crysol, the maximum order of harmonics (up to 50), order of the Fibonacci grid (max 18), and the contrast of the hydration shell (default: 0.03 e/Å^3). The last line contains the checkboxes for additional Crysol options.

1.3.2. SANS Computation Options module.

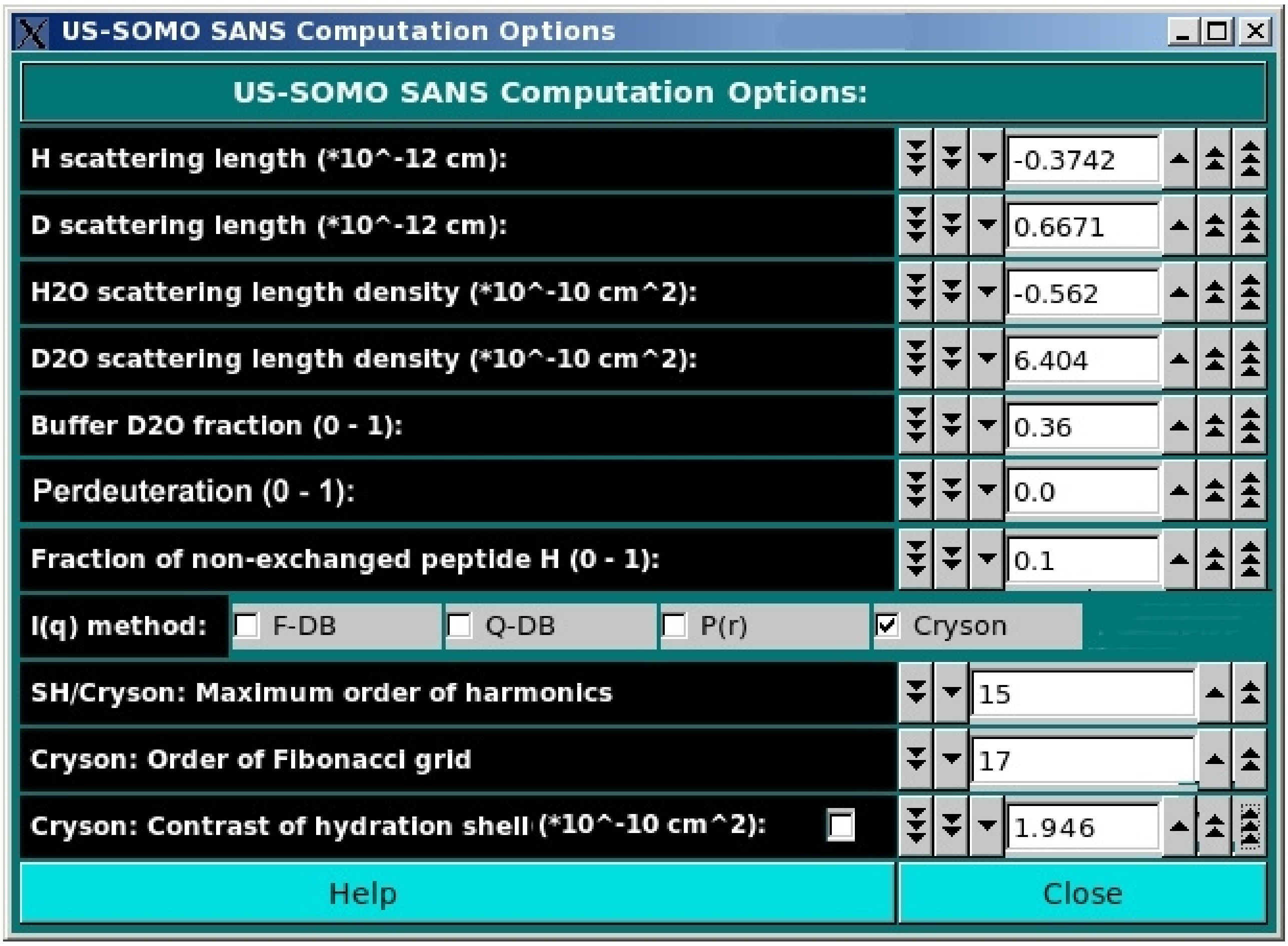

Figure S6 The US-SOMO SANS computation options module.

Several parameters needed for the computation of *I(q) vs. q* and *P(r) vs. r* from atomic structures can be set/modified in this module: H and D scattering lengths, H_2O and D_2O scattering length densities, the D_2O fraction in the experimental solvent, global perdeuteration, and the fraction of non-exchangeable peptide hydrogens. Of the listed computation methods, only Cryson is presently (June 2013) available, with the maximum number of harmonics and the order of the Fibonacci grid that can be set in the corresponding fields. As for the contrast of the hydration shell, it is computed from the D_2O fraction (see Cryson manual), but the value proposed can be overridden by checking the checkbox provided and entering a different value.

1.3.3. Curve Generation Options module.

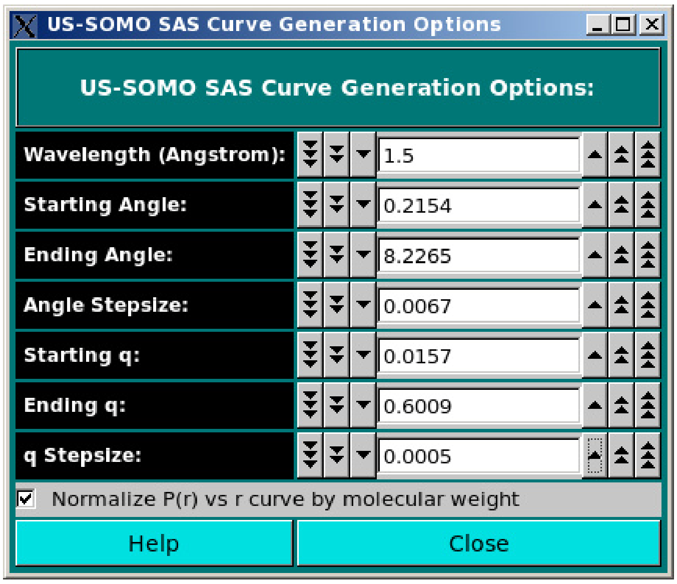

Figure S7 The US-SOMO curve generation options module.

The wavelength at which the *I(q) vs. q* curves will be calculated is set here in the first field (default: 1.5 Å). The starting and ending scattering angles (2θ), and the step size or, alternatively, the starting and ending *q*-values, and the *q* step size, are set here in the next six fields. Note that changing values in the angle fields will automatically recompute the *q*-values in the other fields, and *vice versa*. The normalization option of the *P(r) vs. r* curves by the molecular weight of the macromolecule studied can be also selected/deselected here.

1.3.4. Bead Models Options module.

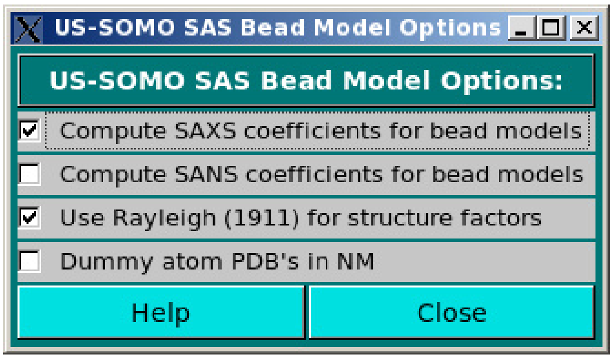

Figure S8 The US-SOMO bead models options module.

This module allows selecting the computation of the SAXS and/or SANS coefficients when generating bead models from atomic scale structures (first two checkboxes), and to optionally use the Rayleigh (Rayleigh, 1911) structure factors for spheres of uniform electron density (third checkbox). This procedure is still under development, and will be fully described elsewhere. The last checkbox is used when uploading bead models whose scale units are not in Å but in nm, like some DAMMIN-generated models.

1.3.5. Guinier Options module.

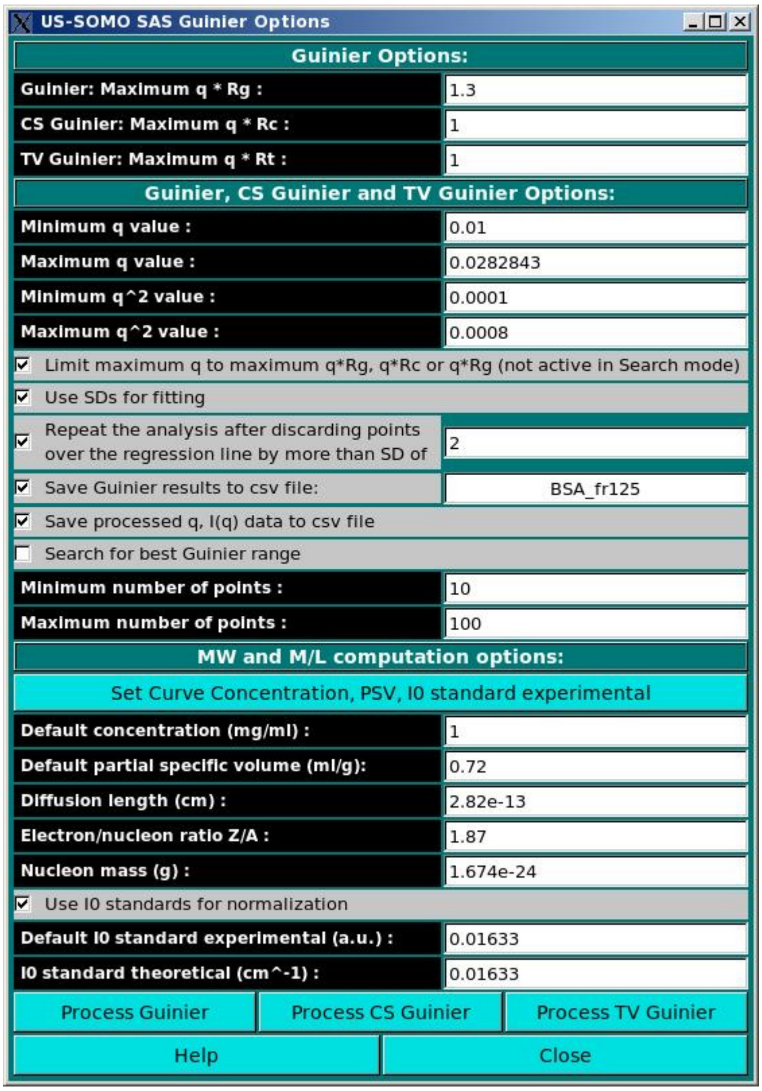

Figure S9 The US-SOMO Guinier options module.

This module opens when the "Guinier" button is pressed in the main SAS panel. After setting/revising the options and the parameters, three different Guinier analyses can be launched by pressing "Process Guinier" (conventional Guinier, $ln[I(q)]$ *vs.* q^2), "Process CS Guinier" (cross-section Guinier for rod-like particles, $ln[q\,I(q)]$ *vs.* q^2), or "Process TV Guinier" (transverse Guinier for disk-like particles, $ln[q^2\,I(q)]$ *vs.* q^2), respectively. The limiting range for all Guinier linear regression are set in the three fields at the top, "Guinier: Maximum q * Rg", "CS Guinier: Maximum q * Rc", and "TV Guinier: Maximum q * Rt", respectively, where "Rg" is the radius of gyration, "Rc" is the cross-section radius of gyration, and "Rt" is the transverse radius of gyration. Next the q^2 range can be set by either changing the "Minimum q value" and "Maximum q value" fields, or the "Minimum q^2 value" and "Maximum q^2 value" fields (entering a value in a q field will automatically update the corresponding q^2 value, and vice versa). In manual processing mode, the limiting factors previously entered can be used to limit the max q value by checking the "Limit maximum q to maximum q*Rg…" checkbox. Standard deviations associated with the data can be used to do a weighted linear regression by checking the "Use SDs for fitting" checkbox. Outliers can be automatically

discarded from the fitting by checking the “Repeat the analysis after discarding points over the regression line by more than SD of” and entering a value in the field (default: 2 SD). Guinier analysis results and processed $I^*(q)$ data (see below) can be saved in a csv-style file by checking the “Save Guinier results to csv file” and “Save processed q, I(q) data to csv file” checkboxes, respectively. A search for an optimal range for fitting can be performed by checking the “Search for best Guinier range” checkbox, which, however, will still be limited by the chosen q^2 range setting. The minimum and maximum number of points to be used in the regression can also be set here in the following two fields. The second half of the module is dedicated to the computation of the $<M>_w$, the $<M/L>_{w/z}$, and the $<M/A>_{w/z}$ values from the corresponding intercepts $<I(0)>_w$ of the Guinier plots. For data manually loaded into the SAXS/SANS module, the concentration, partial specific volume, and experimental $I^{std}_{exp}(0)$ of a standard scatterer can be set by pressing the “Set Curve Concentration, PSV, I0 standard experimental” button. This will open a panel listing all loaded files, and their associated concentration, psv and standard experimental $I^{std}_{exp}(0)$ in three successive, editable fields (Fig. S10; default values: 1 mg/ml, 0.72 ml/g, 0.016633 a.u.).

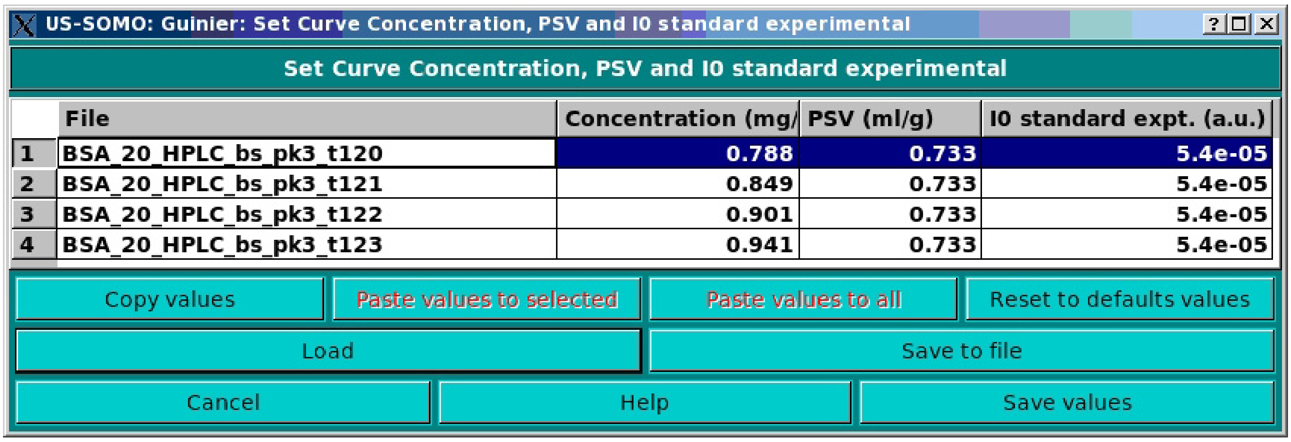

Figure S10 The set concentration, partial specific volume, and I_0 standard experimental pop-up menu.

When multiple datasets are present, values in each line can be copied and pasted to other lines or to all lines by using the appropriate buttons. These fields are automatically updated with appropriate values if datasets are transferred from the HPLC module and contain this information (see below).

The conversion from the corresponding $<I_{exp}(0)>_w$ from the Guinier plots to the $<M>_w$, $<M/L>_{w/z}$, and $<M/A>_{w/z}$ values is done by first putting the data on an absolute scale by normalizing for the $I^{std}_{exp}(0)$ according to:

$$I_{abs}(0) = \frac{I_{exp}(0) * I^{std}_{abs}(0)}{I^{std}_{exp}(0)} \tag{S4}$$

Then the reduced $I^*(0)$ in g mol^{-1} are obtained as:

$$I^*(0) = \frac{I_{abs}(0)\, N_A}{\frac{c}{1000} R_e^2 \left[\frac{Z}{A}\frac{1}{m_n} - \bar{v}_2\, \rho_e\right]} \tag{S5}$$

where N_A is Avogadro's number, c is the sample concentration [mg ml^{-1}], R_e is the diffusion length of the electron [cm], m_n is the nucleon mass [g], $\bar{v}_2$ is the partial specific volume of the particle [cm^3 g^{-1}], ρ_e is the solvent electron density [e/cm^3], and Z and A are the number of electrons and of nucleons, respectively, in the particle, whose ratio is usually taken as a constant specific for each class of biomacromolecules. When the "Save processed q, I(q) data to csv file" checkbox is selected, Eqs. S4 and S5 are applied to all $I(q)$ data before saving, converting them to $ln[I^*(q)]$, $ln[q\ I^*(q)]$, or $ln[q^2\ I^*(q)]$, depending on the type of Guinier processing selected. In this way, if the data are re-plotted using external programs the intercepts in the Guinier plots can be directly related to the $<M>_w$, $<M/L>_{w/z}$, and $<M/A>_{w/z}$ values. Defaults values of 2.82×10^{-13} cm and 1.67×10^{-24} g are present in the "Diffusion length (cm)" and "Nucleon mass (g)" fields, respectively. As for the "Electron/nucleon ratio Z/A", the default value is set to the average value for proteins, 1.87, while ρ_e is set ([e/Å^3], internally converted to [e/cm^3]) in the SOMO-SAS computation options panel. Finally, if the data have not been pre-standardized with the use of a known scattering standard, this can be done here by entering the theoretical values of I_0 for the standard in the "I0 standard theoretical (cm^-1)" field (the default value is that of H_2O at 20 °C, 0.0163 cm^{-1}). The default value for the "I0 standard experimental (a.u.)" can be also changed here (the default is set to the theoretical value of H_2O at 20 °C).

1.3.6. Miscellaneous Options module.

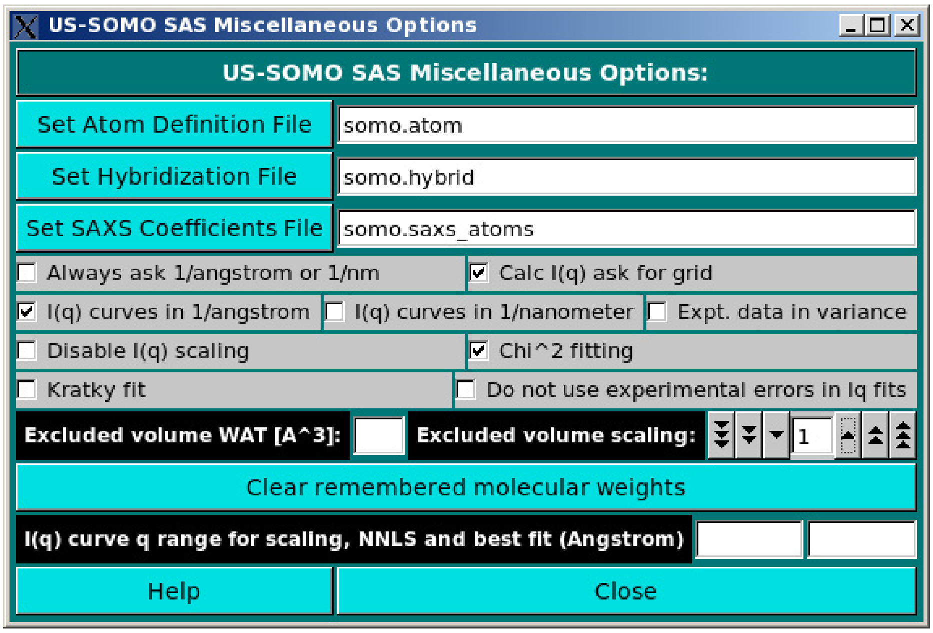

Figure S11 The US-SOMO miscellaneous options module.

In this module, the user can set several options. The top part deals with the user-editable reference files *somo.atom* (containing the atoms' parameters for each residue as listed in the PDB files), *somo.hybrid* (defining the hybridization states used to build the *somo.atom* file), and *somo.saxs_atoms* (defining the SAXS atomic, atomic group, and bead scattering factors). The internal default units of the SAS module can be set to either Å^{-1} or nm^{-1} by selecting one of the "I(q) curves in 1/angstrom" or "I(q) curves in 1/nanometre" checkboxes, but when mixed data are used, the program can be set to ask the units each time a

file is loaded by ticking the “Always ask 1/angstrom or 1/nm” checkbox. If selected, the “Calc I(q) ask for grid” checkbox will ask to match the grid to a plotted grid, otherwise this step will be skipped. The program assumes that the error column in data files reports them as SD, otherwise variance (var = SD^2) can be chosen by ticking the “Expt. data in variance” checkbox. “Disable I(q) scaling” controls if a scaling question is asked each time a new data set is loaded. When performing NNLS, the program can use χ^2 fitting as the minimization criterion if the “Chi^2 fitting” checkbox is selected, otherwise SDs are ignored and NNLS minimizes root-mean square deviation (RMSD). The default method will perform fitting of *I(q) vs. q* data, but Kratky fitting ($q^2*I(q)$ *vs. q*) can be selected by ticking the “Kratky fit” checkbox. Every fitting by default is performed with SD weighting, which can be turned off by ticking the “Do not use experimental errors in I(q) fits” checkbox. If structures have been explicitly hydrated (by using external programs), the volume associated with the hydration waters can be set in the “Excluded volume WAT [A^3]” field. All excluded volumes can be globally scaled (recommended only for studies aimed at finding the “best” values for excluded volumes) by using the “Excluded volume scaling” field. “Clear remembered molecular weights” is used when a different structure is used in generating *P(r) vs. r* functions, which are then normalized by the molecular weight. Finally, the “I(q) curve q range for scaling, NNLS, and best fit (Angstrom)” fields allow limiting the *q*-range of loaded experimental or computed data over which these operations will be performed.

1.4. The US-SOMO HPLC-SAXS module.

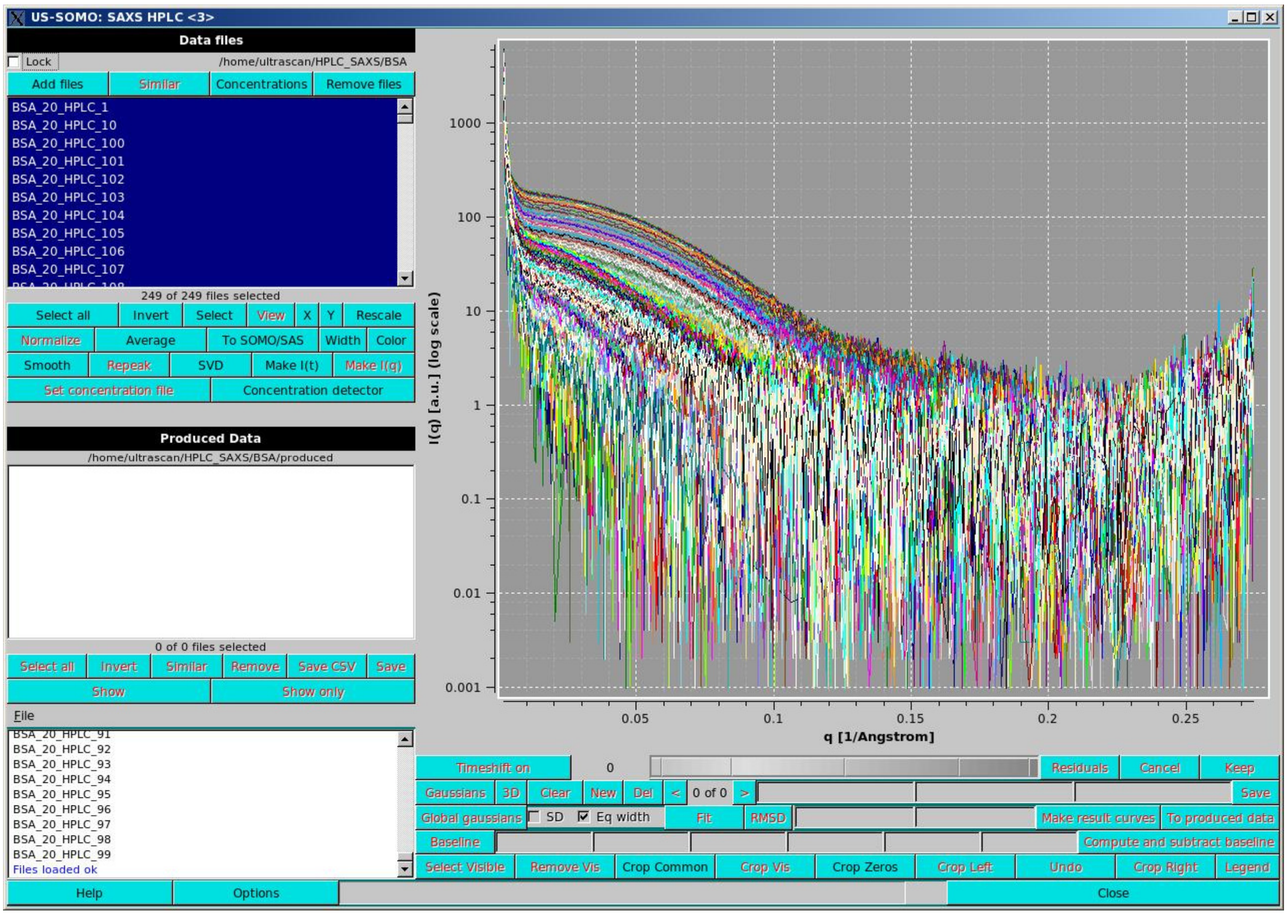

Figure S12 The US-SOMO HPLC-SAXS module. The buttons with the black labels are the ones currently active, the ones with the red labels become active when allowed by the processing/visualization stage. The graphics panel shows a collection of HPLC-SAXS $\log_{10}[I(q)]$ *vs.* q frames data for a BSA separation (see Materials and Methods). The permanent upturn at very small q-values is due to biological material aggregated in this case by the intense X-ray beam on the capillary cell walls.

The first operation is to load experimental data files using the "Add" button on the top left panel.[1] An operating directory can be pre-selected by clicking on the path shown above it, and navigating in the file system (clicking the "Lock" checkbox will fix that directory). "Similar" will select files with similar names and allow manual pattern matching entry if no new similar files are selected, while "Remove" will discard previously selected files (see below); if the files were produced by the module, and were not previously saved, a warning window will pop-up, allowing to proceed or to stop removing the selected items. The file format for SAXS data recognized by the US-SOMO HPLC-SAXS module consist of .dat files with two or

1 All data are dynamically allocated, therefore memory available is a limitation. We have tested loads and processing of hundreds of frames without issue. Further processing of hundreds of frames can produce thousands of resulting curves and this is supported. Of course, as the size of the data increases, the time required to process the data will increase, and this may be the practical limit.

three TAB or space -separated columns containing the *q*, *I*(*q*), and optionally their associated SD values, respectively. Each frame number (or time value) must be present somewhere in the filename with a common prefix and suffix[2]. If a block of files are selected for uploading and they have a recognizable frame number, they will be listed ordered by increasing frame number. The currently recognized format for concentration data is similar to the SAXS data format with the addition of the string “Frame data” in any place on the first line. The two or three columns of data will the frame number, concentration-related data, and optionally an associated SD value. *I(q) vs. q* and concentration data frames are automatically recognized and the labels on the *x*- and *y*-axes are then properly set (*e.g.*, Fig. S12). “Concentration” will show every file listed together with their associated concentration (mg/ml), if appropriate and properly set (see below). Concentrations can also be entered and modified manually. They can be used to normalize the *I(q) vs. q* data (see below). Loaded files can be displayed on the graphics panel by individually clicking on them (shift-click will select a contiguous series, ctrl-click allows multiple irregularly spaced selections). Produced data will also show up in this panel with associated putative filenames. “Select all” will select all files, and “Invert” will allow toggling the selection between selected files and everything else not currently selected. “Select” will open up a panel in which several selection and search options can be performed (see Fig. S23). “View”, active when up to ten datasets are selected, will show them in text format. The “X” and “Y” buttons allow to toggle between linear and $\log_{10}$ scaling of the data on the *x*- and *y*-axes (if zero or negative values are present, they will be temporarily removed as they cannot be shown on the display in the $\log_{10}$ mode). “Rescale” adjusts the X-Y axes on the graphics window to maximize the display of selected datasets (no effect on the data themselves). “Normalize” will divide the *I(q)* data by the stored/entered concentrations. “Average” will produce a weighted average with propagated SDs of selected data. ”To SOMO/SAS” will transfer selected datasets back into the US-SOMO SAS panel. Each time the “Width” button is pressed, it increments the data line size of the plots, until it goes back to the initial value. “Color” shifts the colours used in the graphics window for a better contrast with the background. “Smooth” performs a regularization of selected data using a moving window, whose dimension is defined in a pop-up menu, using a Gaussian smoothing kernel of $2n + 1$ points. “Repeak” is used to effectively scale data on the Y-axis to a pre-set target, selectable in a pop-up window among the data subjected to this operation (this affects the data, a new file is generated with “rp” and the scaling factor added at the end of the filename). “SVD”, operating only on *I(q) vs. q* datasets, opens a pop-up window were a single-value decomposition analysis (*e.g.*, Williamson *et al.*, 2008) can be performed on the selected data. The “Make I(t)” and “Make I(q)” buttons are crucial for the HPLC module operation: they allow to generate a series of chromatograms (*I(t) vs. t*, where *t* can be real elution time or frame number) for each *q*-value present in the original data files, and to re-generate *I(q) vs. q* files for each frame after data treatment in frame- (or time-) space, respectively. "Set concentration file" will select an already uploaded file containing the UV or refractive index profile *vs.* time or frame number as the source of the concentration-

---

2 For example, data1saxs.dat, data2saxs.dat, data3saxs.dat will be recognized as frames 1,2,3, where “data” and “saxs” can be replaced by any common sequence of characters. Consequently, 1.dat, 2.dat, 3.dat would be acceptable, but abc1.dat, qrs2.dat, xyz3.dat would not, because the prefix characters are not common.

dependent signal. The type of detector can be selected and its calibration constant entered in the pop-up window that opens when pressing "Concentration detector".

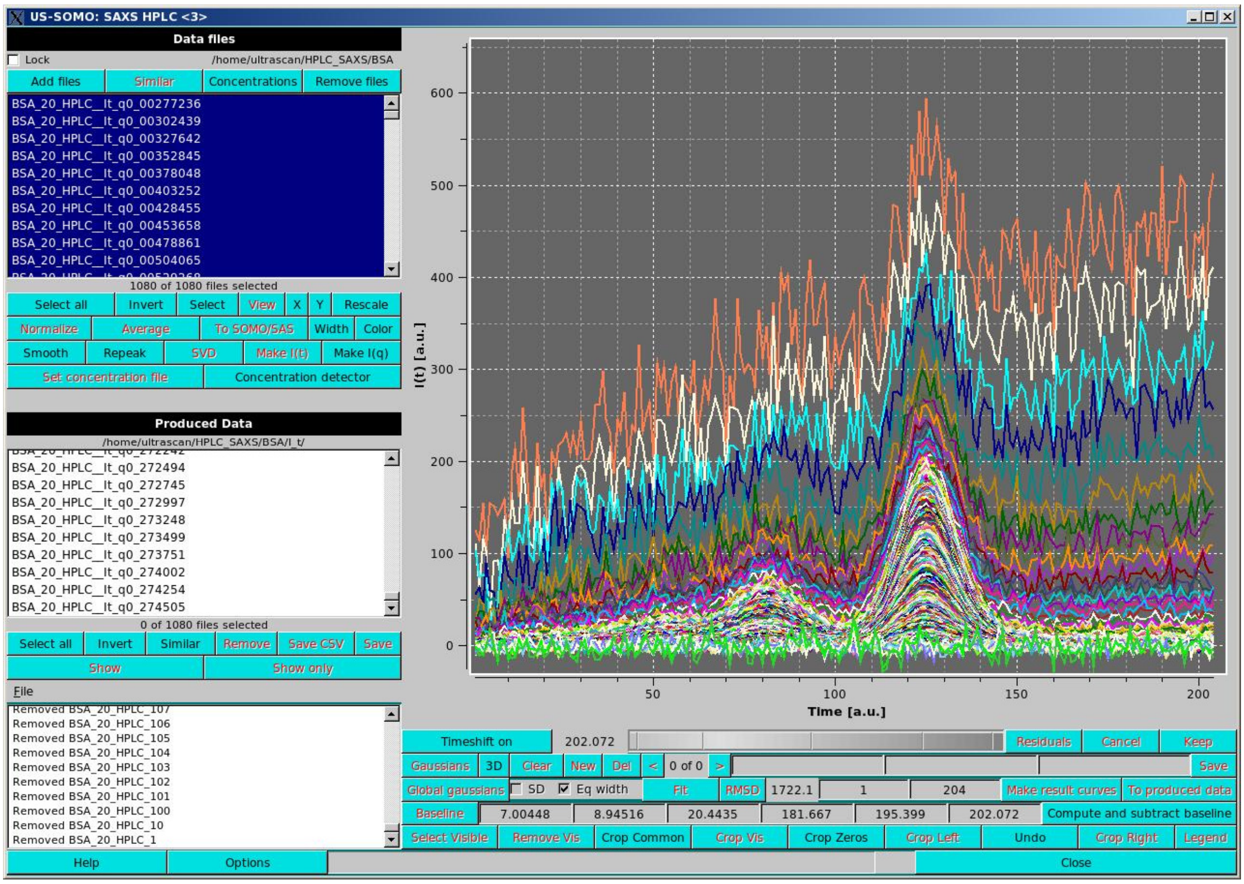

Figure S13 The BSA HPLC-SAXS data shown in Fig. S12 after transformation to a series of *I(t) vs. t* chromatograms for each *q*-value.

In Fig. S13, the original *I(q) vs. q* data of Fig. S12 have been transformed to *I(t) vs. t* data (the *q* values are part of the resulting filenames). Some cropping operations (see below) were also performed to remove very noisy low-*q* datasets, followed by display rescaling. All operations are recorded in the bottom left panel. The file names of produced data are shown in the Produced Data panel to the centre-left, and can be selected and saved to files using the appropriate buttons below it. “Similar” will search for similar file names after selecting a single file in this panel; “Show” will add the selected file to those shown in the graphics window, while “Show only” will remove other files from the graphical display. Two types of files can be produced, csv-style ("Save CSV") or regular 3-columns .dat files ("Save").

Below the graphics display window are all the commands for performing baseline analysis and Gaussian decomposition of the *I(t) vs. t* data. At the bottom of the panel are located a series of buttons for graphics and selection control. “Select visible” will select files shown in the graphics window, which can be zoomed using the mouse (left click). For instance, this is a practical way of selecting only a few files, by zooming on a region where only them are present, or of removing them by pressing the “Remove visible” button. “Crop common” will crop all selected files so that they have identical *x*-axis values by dropping points outside of the union of all selected file's *x*-axis values, while “Crop Visible” will remove what is shown in the graphics

window. “Crop Zeros” will remove data having zero or negative values in the intensity columns (discouraged: a warning panel will pop-up, asking the user whether she/he really wants to proceed). “Crop Left” and “Crop Right” will remove one point on the left or right of the selected data, respectively. “Undo” will undo the last operation. “Legend” will turn on below the graphics window a display of the correspondence between colours and filenames (automatically disabled if the selected files are ≥20).

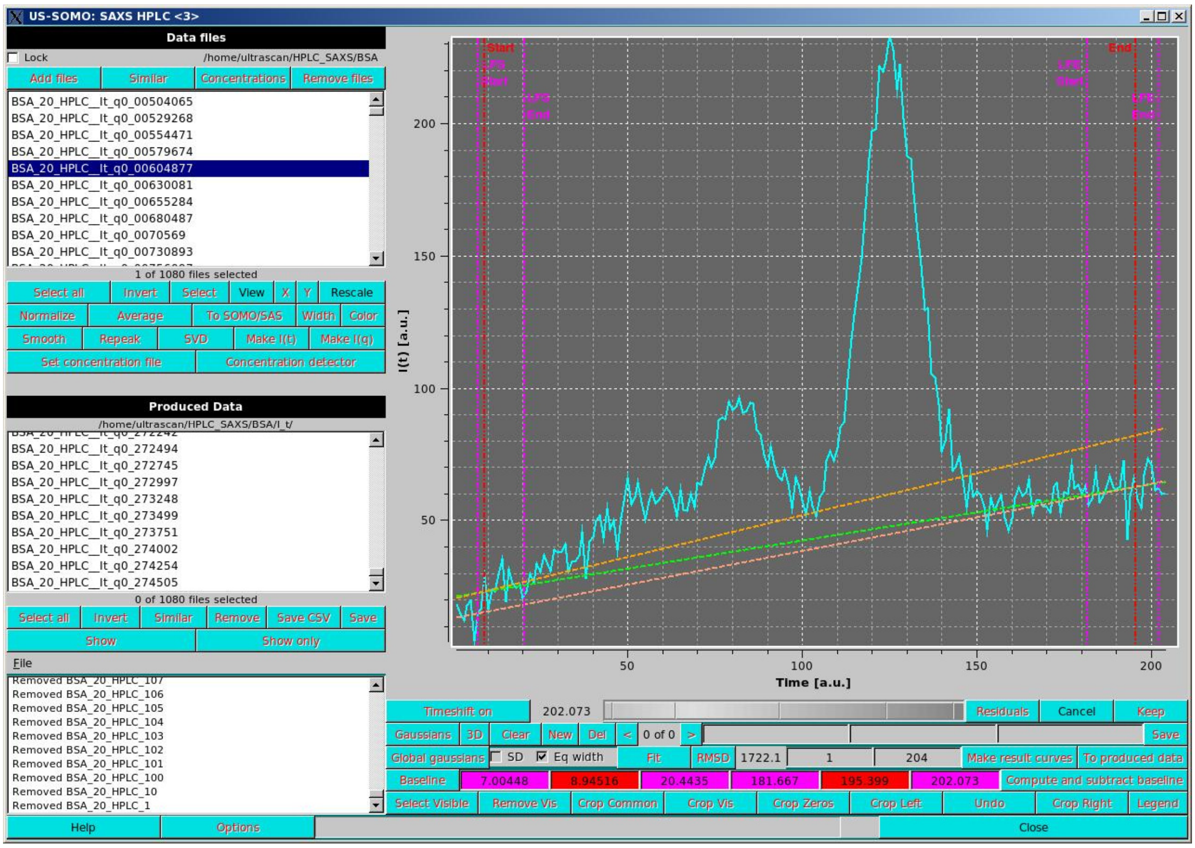

Figure S14 Baseline setting in the HPLC-SAXS module. The non-zero baseline is presumably due to the accumulation of aggregated material on the capillary walls, due to X-ray irradiation.

After visual inspection of the *I(t) vs. t* chromatograms, baselines can be defined, if needed. A single chromatogram is first selected (a compromise between high intensity and low noise works best), and then the “Baseline” button is pressed. This superimposes to the selected chromatogram six vertical lines (Fig. S14), three for each side. The two magenta lines on each side define the beginning and end, respectively, of the chromatogram regions over which the data are averaged to set the beginning and end of a baseline. The red lines define instead the beginning and end points of the data to be subjected to the baseline correction. The positions of the six lines are shown in the six fields to the right of the “Baseline” button, with their background colour-coded accordingly. By clicking on each field, the corresponding line can be moved across the chromatogram using the gray-shades bar-wheel at the top. The actual baseline is shown as a green dashed line, while the two orange dashed lines show the trends of linear regression done on the regions delimited by the two couples of vertical magenta lines. Ideally, the orange lines should come as close as possible to the green line. Once a reasonable baseline has been found, pressing “Keep” will keep its parameters (initial and end points, slope) for further operations. “Cancel” will remove them and revert to no baseline. It is best to

then select one by one a few other chromatograms and press "Baseline" again to see how the chosen settings perform for other datasets. If necessary, the settings can be modified and replace the initial ones.

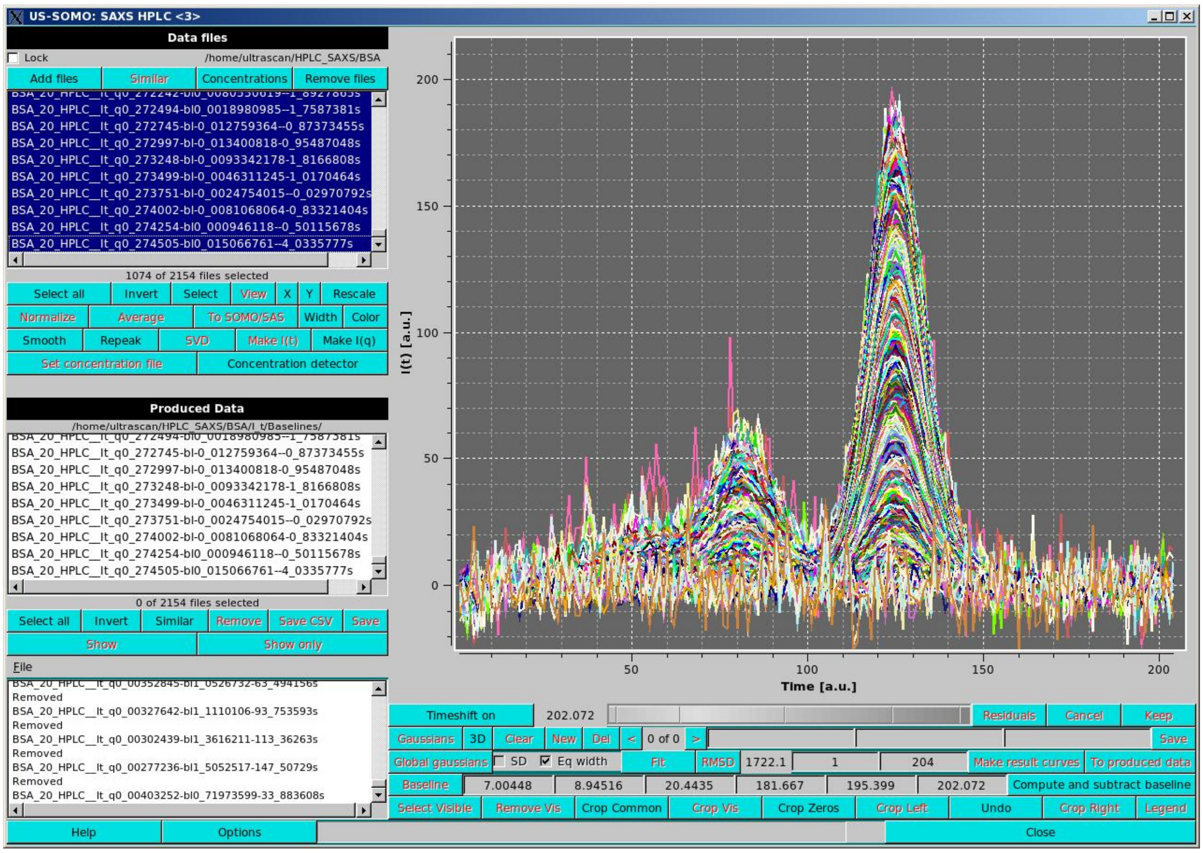

Figure S15 Set of *I(t) vs. t* chromatograms after baseline subtraction.

After selecting multiple *I(t) vs. t* chromatograms, the baseline parameters thus set can be applied to all curves with concurrent subtraction of each baseline by pressing the "Compute and subtract baseline" button (see Fig. S15). The initial and final points used in the baseline subtraction are added to the filename of the produced files. If Gaussian analysis is not required, a series of *I(q) vs. q* frames can be re-created at this stage from the baseline-corrected data by pressing the "Make I(q)" button.

Before proceeding to Gaussian analysis, a single-value decomposition (SVD) analysis could be useful. In SVD analysis, the number of significant singular values in the decomposition should be equal to the number of components in the data, and thus to the minimum number of Gaussians required to accurately reconstruct the data. A single SAS experimental dataset is typically represented as $I(q)$, where q is a grid of $\{q\}_{i=1}^{m}$ points. A sequence $\{t\}_{j=1}^{n}$ of n $I(q)_{t(j)}$ on the same q-grid can be assembled into a $m \times n$ matrix $I = [I_{ij}] = [I(q)_{t(1)}, I(q)_{t(2)}, ..., I(q)_{t(n)},]$. Each column of I contains an $I(q)$ curve for a specific t and each row contains an $I(t)$ curve for a specific q. If standard deviations of the experimental data are available, these can be analogously placed in a matrix S. If a synchronized concentration dataset $C(t)$ is available, it can be added to I as an additional row.

In a SVD analysis, if I is the matrix containing the original data, then:

$$I = USV^T \qquad \text{(S6)}$$

where U is an orthogonal $m \times m$ matrix, S is a diagonal $m \times n$ matrix, and V^T is an orthogonal $n \times n$ matrix. The elements of the diagonal of S are the singular values. Reconstructing an approximation of the matrix I proceeds by setting some diagonal elements of S to zero, forming S' and performing the multiplication

$$I' = US'V^T \tag{S7}$$

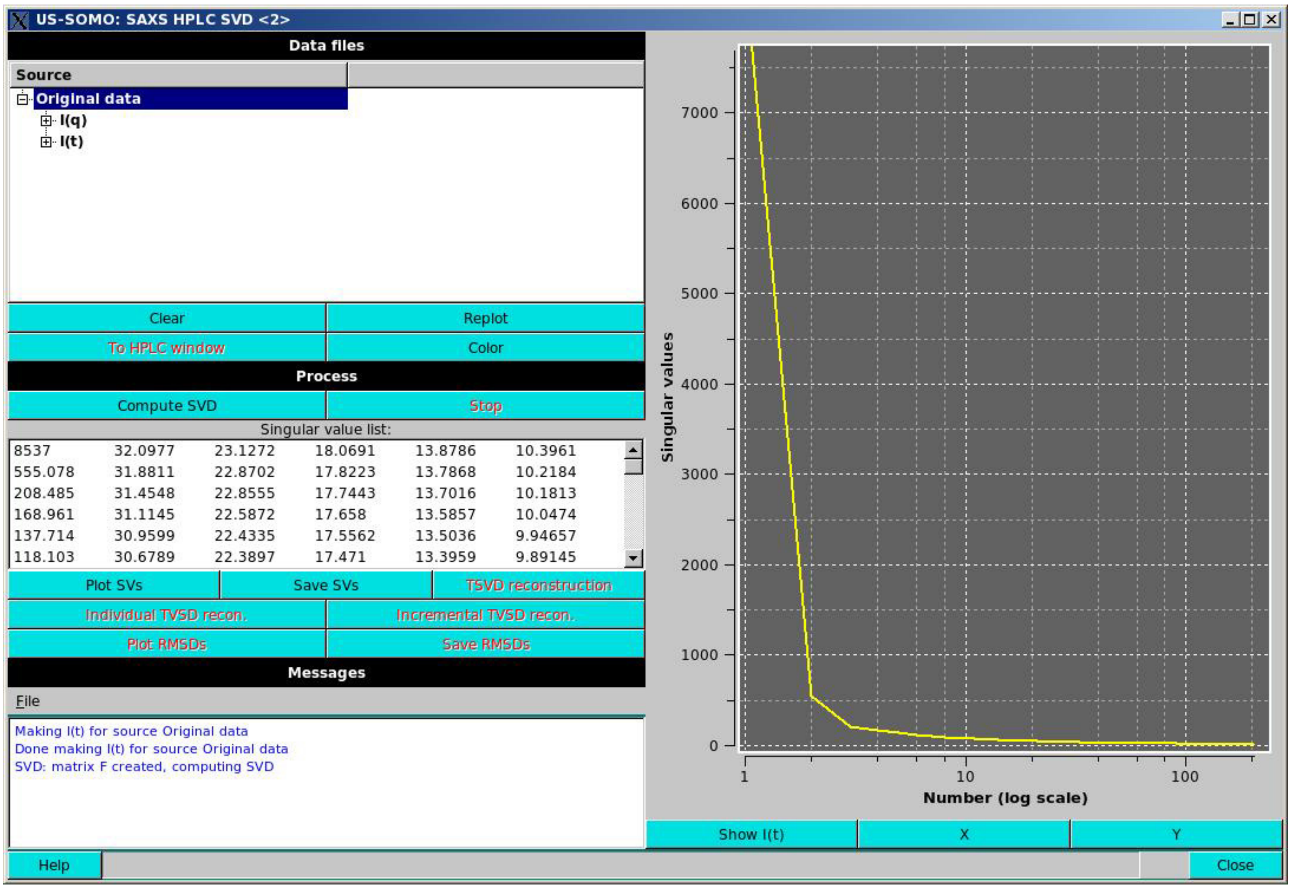

Figure S16 SVD performed on a baseline-corrected BSA dataset, showing the SVs plot.

SVD can be performed either on the original or on the baseline-subtracted *I(q) vs. q* data or subset of data (if significant baseline drift occurs, the SVD will try to fit also that part of the signal). After selecting the data, the “SVD” button will open a new window (Fig. S16). The top left box labelled “Data files” will contain a list of the data set in an expandable format. The first set will be labelled “Original data”. Opening the item will show its contents, “I(q)” and “I(t)”, which can be further expanded to show/select the individual curves in the data set. When a selection is changed from what is currently plotted, the button “Replot” will become active; pressing it will refresh the plot display. For example, selecting “Original data” at the top level and pressing “Replot” will plot the entire dataset, but only in the *I(q)* or *I(t)* mode. The “Show I(t)” or “Show I(q)” toggle button below the plot window will show the *I(t) vs. t* or *I(q) vs. q* view of the data and automatically replot. The “Color” button will rotate the plot colours based upon a pre-defined palette.When a single data set or sub-selection of individual *I(q)* curves from a single dataset are selected and the plot window is in *I(q)* mode, the “SVD” button becomes active. Pressing “SVD” will compute the singular value decomposition (Lawson *et al.* 1995). The singular values (SVs) will be sorted in descending order and placed on the screen in the “Singular value list”. The button “Plot SVs” activates and will plot the SVs in the plot

area by default in a linear *vs.* log scale (Fig. S16). The axes scales can be toggled between logarithmic and linear by pressing the “X” or “Y” buttons below the plot area. The “Save SVs” button will save the SVs to a file. Giving the filename a “.csv” extension will result in a comma separated output, otherwise, the output will be “TAB” separated. Selecting any set of SVs in the “Singular value list” will activate three more buttons: “TSVD reconstruction”, “Individual TSVD recon.” and “Incremental TSVD recon.”. Pressing “TSVD reconstruction” will generate a new dataset in the “Data files” section consisting of the reconstruction of the data based upon the selected SVs. TSVD means a truncated SVD reconstruction (Aster *et al.* 2005), which formally should be computed on the numerically highest SVs, but here we are using the term loosely, to mean reconstruction on any subset of the SVs. The resulting dataset can be selected, expanded and/or plotted identically to the original data. Expanding the TSVD data will show “I(q)”, “I(t)”, “SVs used” expandable subsections and also the root mean squared deviation over the number of points (RMSD) of the expansion and the name of the reference dataset for the reconstruction.

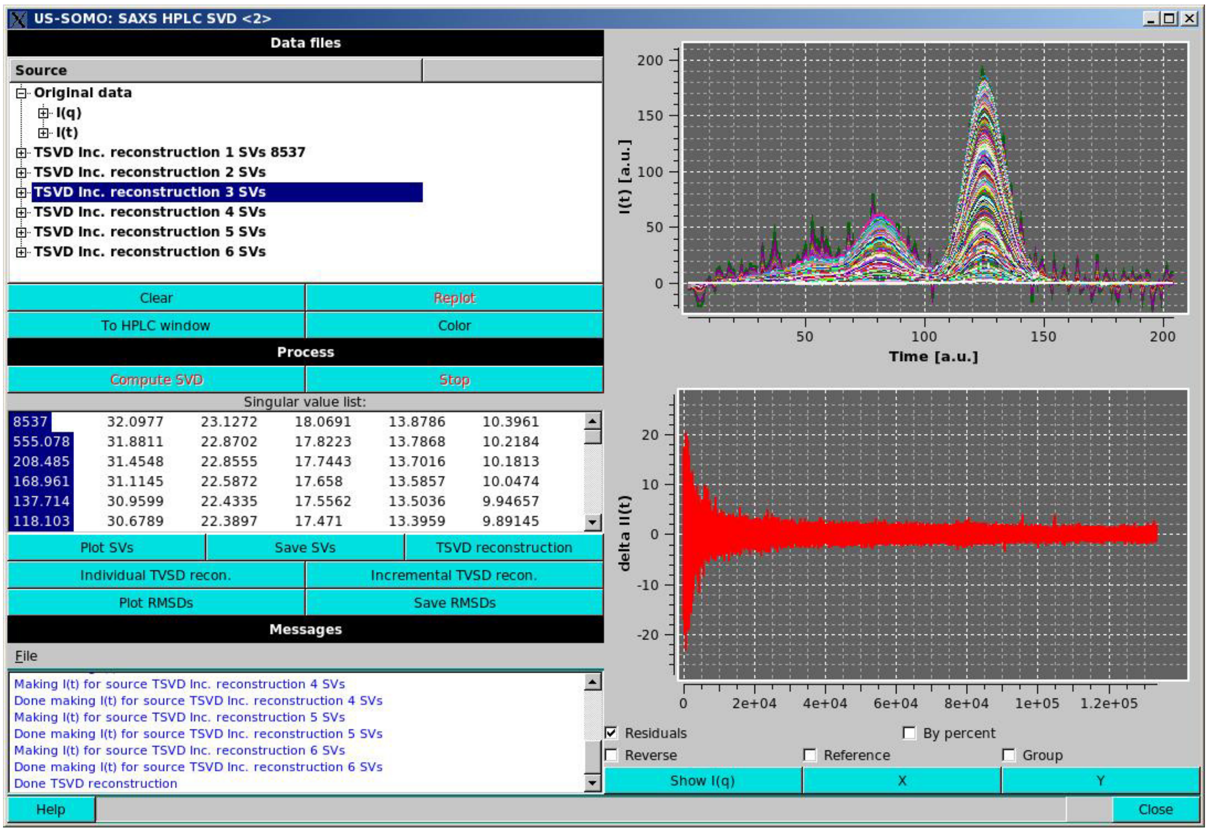

Figure S17 SVD incremental reconstruction of the BSA dataset using the first three SVs. The residuals are plotted in a linearly contiguous manner (“Group” checkbox not selected).

When a single or an incremental TSVD reconstruction is selected, the “residuals” checkbox under the plot appears (see Figs. S17-S18). Pressing this checkbox will plot the residuals of the reconstruction vs. the reference dataset in a new plot area below the main plot area. When residuals are displayed, additional checkboxes will be available under the plot. These include “Use SDs”, which turns off division of the residuals by the SD of the reference dataset and will not be available for data without SDs; “By percent”, which displays the differences by percent; “Reverse” which reverses the Y-axis around zero; “Reference”,

which toggles the display of the reference data in the main plot window; and “Group”, which toggles grouping of the residuals in a superimposed or linearly contiguous manner (see Figs. S17-S18). The button “Individual TSVD recon.” will take the selected SVs and produce a TSVD reconstruction for each value selected individually, resulting in multiple TSVD reconstructions in the “Data files” section. “Incremental TSVD recon.” will take the selected singular values and produce a TSVD reconstruction for the first value selected, then for the first and second values selected, etc., until all the selected values are included in a reconstruction, again resulting in multiple reconstructions in the “Data files” section (Figs. S17-S18).

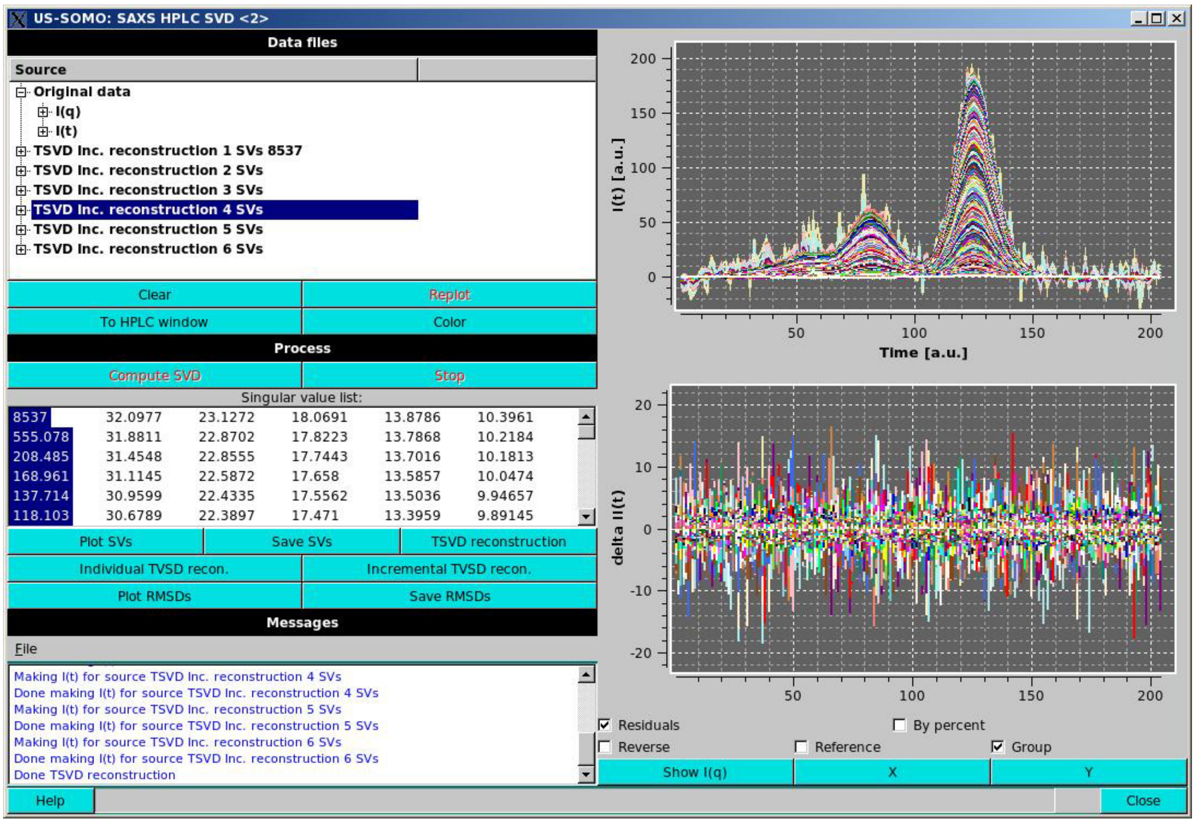

Figure S18 SVD incremental reconstruction of the BSA dataset using the first four SVs. The residuals for each curve are plotted superimposed (“Group” checkbox selected).

Once an individual or incremental reconstruction is computed, the buttons “Plot RMSDs” and “Save RMSDs” activate, allowing plotting and saving analogous to the “Plot SVs” and “Save SVs” mentioned previously. Finally, any reconstructed dataset can be added to the US-SOMO/HPLC-SAXS module by selecting and pressing the “To HPLC window” button. Note only the $I(q)$ or $I(t)$ data will be added depending on the plot mode.

As an example, suppose one wants to determine the number of components present in a set of $I(q)$ curves. After bringing them into the SVD module as described above, the SVD can be then computed. By looking at the SVs plot, one can evaluate that at most N singular values seem reasonable to reconstruct the dataset. One would then select the numerically largest N values in the SV list and run an incremental reconstruction. Subsequently, each reconstructed dataset could be compared by RMSD and visually to determine the effect of adding additional singular values to the reconstruction to assist the determination of the minimum number

of singular values required to accurately reconstruct the original data. Another check would be to run the individual reconstruction on the same set of selected singular values and inspecting the individual datasets visually (preferable via $I(t)$ plots) to see if there seems to be signal present in reconstructions past a minimum number of singular values. In this way, the US-SOMO/HPLC/SVD module can be used to approximate the number of independent components present in a HPLC experimental dataset or even a concentration series.

Given the matrix I containing columns i of $I(q)$ and rows j of $I(t)$, the principles of Gaussian analysis can be schematized as follows.

Single curve fitting:

Pick a row i of I and define a set of p Gaussians $\{G_k^i(t)\}_{k=1}^p$, with amplitudes a_k^i, centres b_k^i, and widths c_k^i. Then:

$$G_k^i(t) = a_k^i e^{-\frac{(t - b_k^i)^2}{2(c_k^i)^2}} \quad \text{(S8)}$$

In US-SOMO/HPLC, we let the user visually place the centres b_k^i, and subsequently provide several methods for fitting (see below) by minimizing over, in general, $3p$ variables, a_k^i, b_k^i, and c_k^i:

$$\sum_j \left[\left(\sum_k G_k^i(t_j)\right) - I_{ij}\right]^2 \quad \text{(S9)}$$

or in the case that $\forall j{:}S_{ij} \neq 0$ (*i.e.*, i^{th} row of S has no zero elements):

$$\sum_j \frac{\left[\left(\sum_k G_k^i(t_j)\right) - I_{ij}\right]^2}{S_{ij}} \quad \text{(S10)}$$

In the program, there are options to fix a combination of individual Gaussian curves k, amplitudes a, centres b, and widths c, which would result in fewer that $3p$ variables during the minimization. Constraints, in percentage from previous value or from the initial value, are also available for a, b, and c.

Global Gaussians:

In the US-SOMO program, entering the "Global Gaussian" mode does a fit of the preset single curve $\{G_k^i(t)\}$ against every curve $i = 1, ..., m$, keeping the centres b and widths c fixed. This provides an initialization of the amplitudes a for all curves as a starting point for global fitting or for refinement/ extension to other datasets a previous global fitting on a subset of data.

Global fitting:

Given a $\{G_k^l(t)\}$ for a specific row $i = l$ from the result of a single curve fitting, one can globally fit over the amplitudes a_k^i by utilizing common centres, $b_k^i = b_k^l$ for $i = \{1, ..., m; i \neq l\}$, and common widths, $c_k^i = c_k^l$ for $i = \{1, ..., m; i \neq l\}$, and then doing a global minimization over the $pm + 2p$ variables a_k^i, b_k^l, c_k^l, again using eqs. S9-S10. Global fitting is currently only available with Levenberg-Marquardt minimization routine. As in the single Gaussian fitting, there are options to fix a combination of individual Gaussian curves k, amplitudes a, centres b, and widths c, which would result in fewer variables during the minimization. Constraints, in percentage from previous value or from the initial value, are also available for a, b, and c.

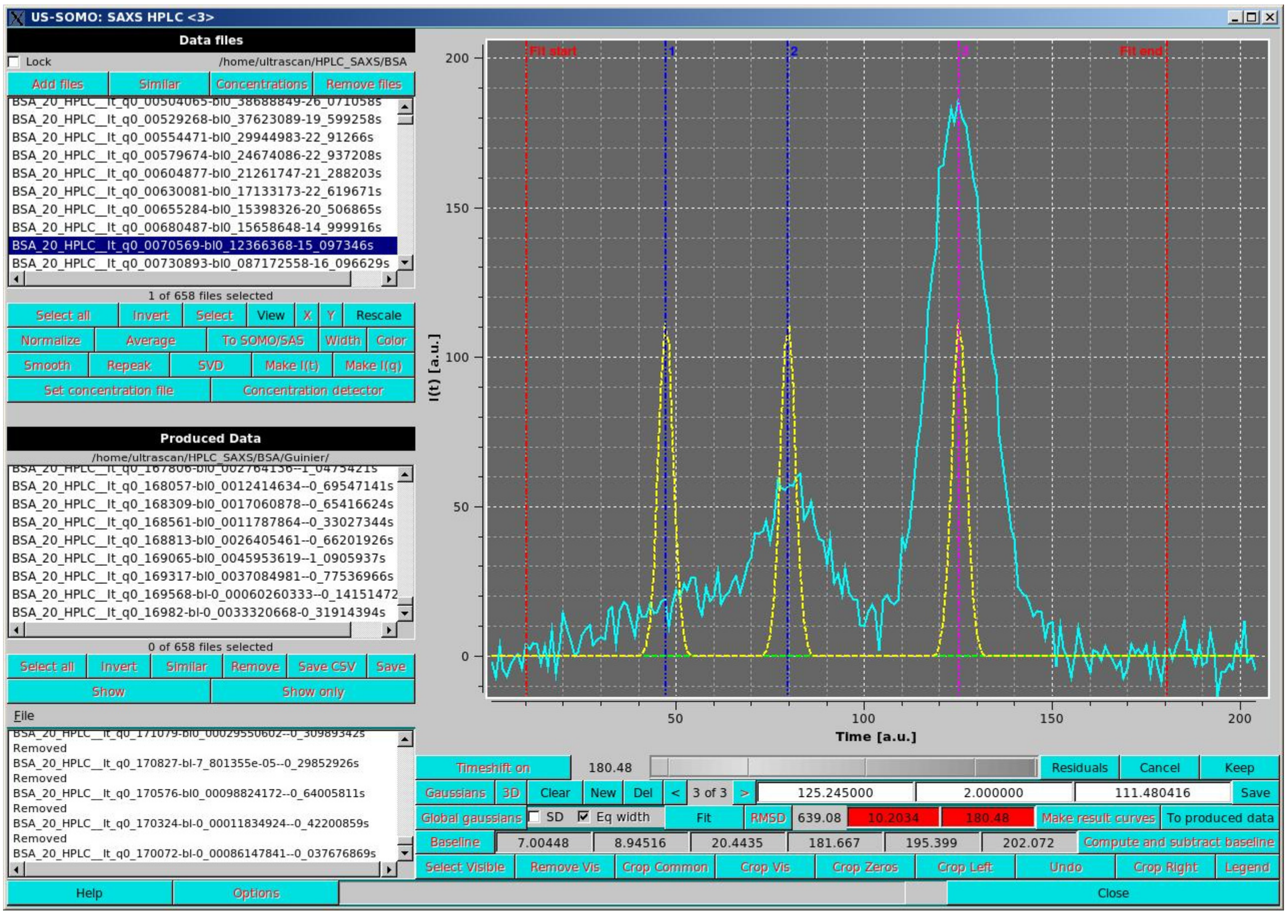

Figure S19 Initial Gaussians positioning.

The baseline-subtracted data can be subjected to Gaussian analysis by first selecting a single chromatogram, and then pressing the “Gaussians” button. If a previously-generated set of Gaussians was present or loaded from file, the Gaussians will show up under the peak(s) together with vertical lines indicating their centres. “Clear” will remove them, and allow to start a new analysis. Each time the “New” button is pressed, a new Gaussian will be added (green colour), with pre-set centre, width and amplitude shown in the three rightmost fields. By clicking on each field, and then using the gray-shades bar-wheel, each Gaussian can be adjusted to initialize the process (usually, only the centres need to be positioned under the peaks). Clicking on the “<” and “>” buttons will toggle among the Gaussian present, whose identifying number is shown in the field between them. The active Gaussian is identified by a magenta vertical line positioned at its centre, while blue lines are used for the others. The limits for the analysis of the chromatogram are shown with two vertical red bars, whose position is shown in the two red-background fields at the left of the “Make result curves” button (Fig. S19). Once the initialization is completed, pressing the “Fit” button will bring up a window controlling the fit procedure, shown in Fig. S20.

The first three lines in the fit panel control the centres, widths, and amplitudes of the Gaussians. For each of these parameters, it is possible to fix them to the initial values (checkboxes on the left side), or to allow a % variation (default: 5%) from either the initial values (if the rightmost checkboxes are selected) or based on each cycle of iterations-generated values. It is possible to also individually fix each Gaussian by selecting the

corresponding checkbox on the "Fix Gaussians" line; the program will automatically present as many checkboxes as are the input Gaussians. The "Epsilon" field controls the step used in computing the discrete derivative, gradient or Jacobian. The number of iterations/cycle is set in the "Iterations" field (default: 100), while the "Maximum calls" controls the attempts to improve at each stage of the minimization.

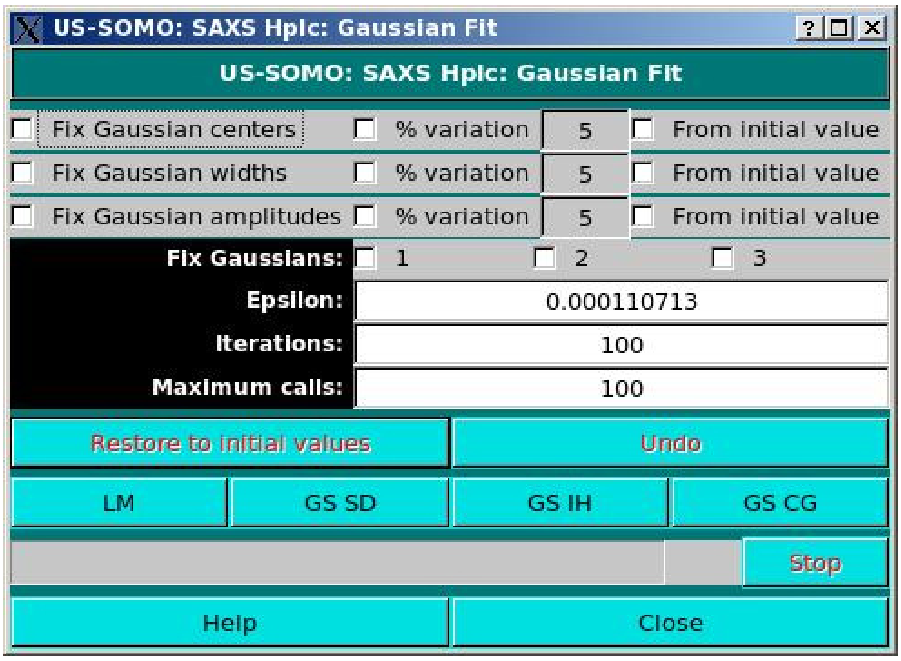

Figure S20 The Fit panel controlling single and global fit operations.

Several fitting algorithms are available through dedicated buttons: Levenberg-Marquardt (LM), Gradient Search Steepest Descent (GS SD), Gradient Search Inverse Hessian (GS IH), and Gradient Search Conjugate Gradient (GS CG). When initially fitting a single chromatogram, normally a first iteration cycle is performed with the centres fixed, and then a second is sufficient with no constraint or with a restraint on the peak centres from initial values (default 5%) to find a good set of Gaussians. The resulting Gaussians are updated in the graphics window, and their sum is shown as a dashed yellow line, so that the goodness of the fit can also be graphically assessed. The RMSD of the fit is also updated continuously in its main panel field. The "Restore to initial values" and "Undo" buttons are available to restart the fit procedure from the beginning, or to undo the last operation, respectively. "Close" will close the Fit window when a satisfactory fit is obtained. A typical result is shown in Fig. S21.

Back to the main window, the "SD" checkbox will allow the use of the errors present in the original data to weight each point in the fit (this applies to every fit operation, if selected). If some datasets have zero or NaN values for one or more SD values, a pop-up menu will appear listing all the files presenting this problem, and with how many occurrences. The user can then select between three options: drop the datasets containing these non-positive SDs; drop just the frame (or time) point missing the positive SD(s); or not use SD weighting. The "Eq. width" checkbox, which cannot be deselected in non-expert mode operation, will instead either keep fixed the width of the Gaussians when doing global Gaussians, or optimize also the common widths of each Gaussian family for all *q*-values when doing a global fit.

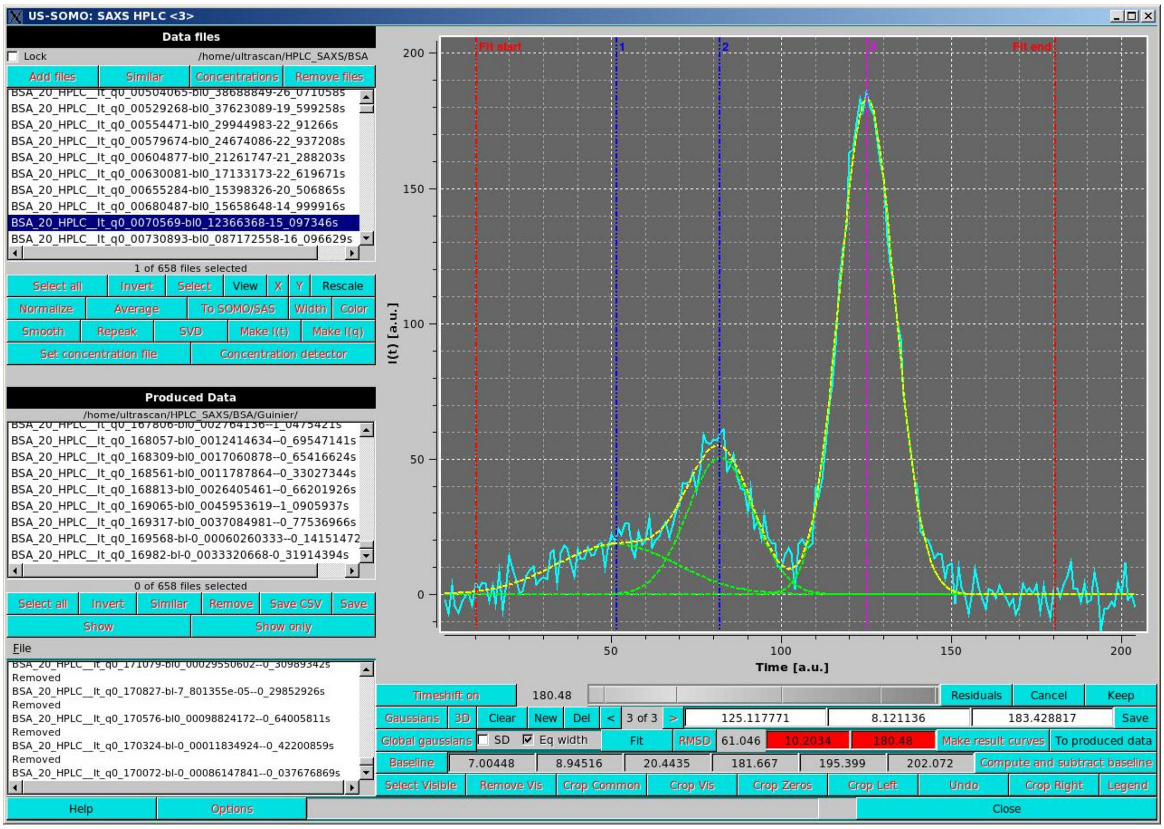

Figure S21 Final single-chromatogram Gaussians after fitting.

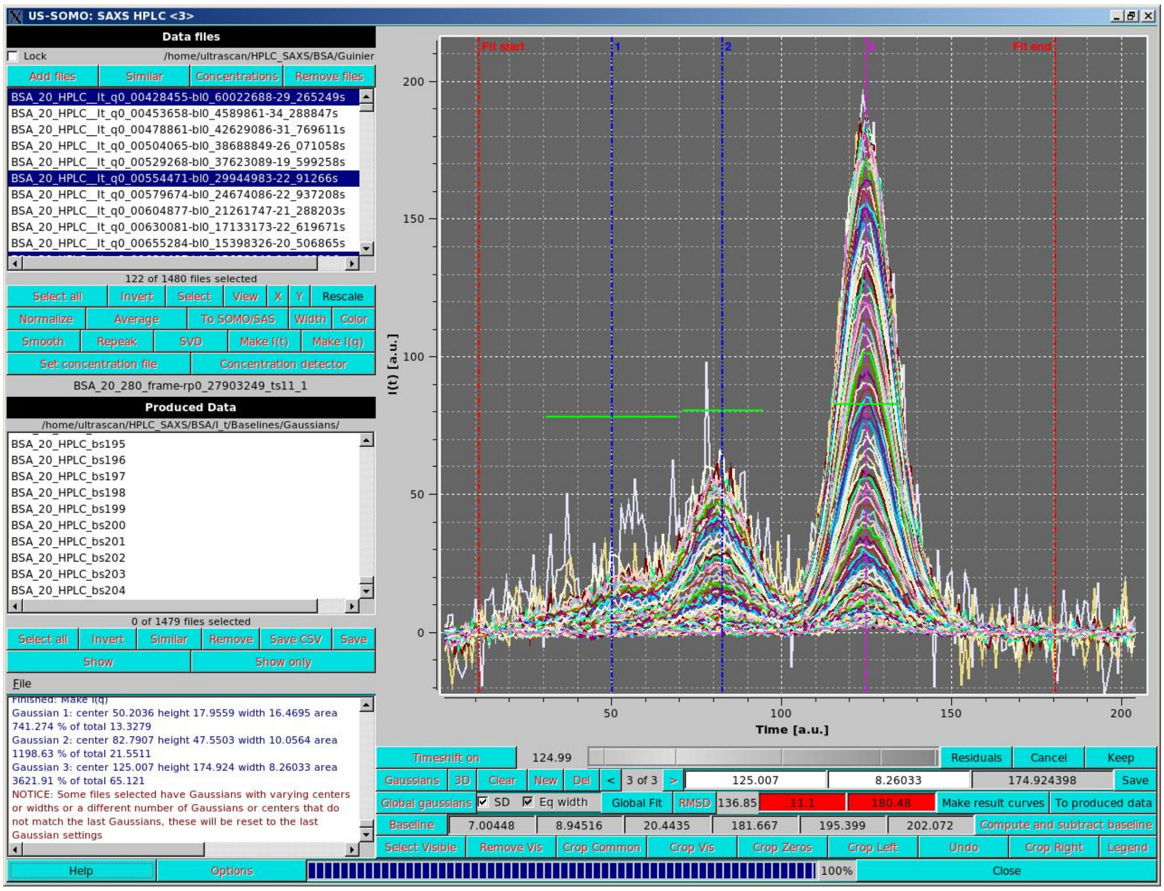

Figure S22 Global Gaussians results applying the parameters found on a single Gaussian to multiple selected files; SD weighting was selected.

These two operations can be done on multiple selected files once the initial single chromatogram Gaussians are accepted by pressing the “Keep” button (“Cancel” will reset them to the initial values). “Global Gaussians” will simply find the amplitudes best fitting all the selected chromatograms based on the centres and widths found on the initial chromatogram. It is best to first select only an equally spaced subset of data by using the “Select every Nth” option available in the pop-up window appearing on pressing “Select”.

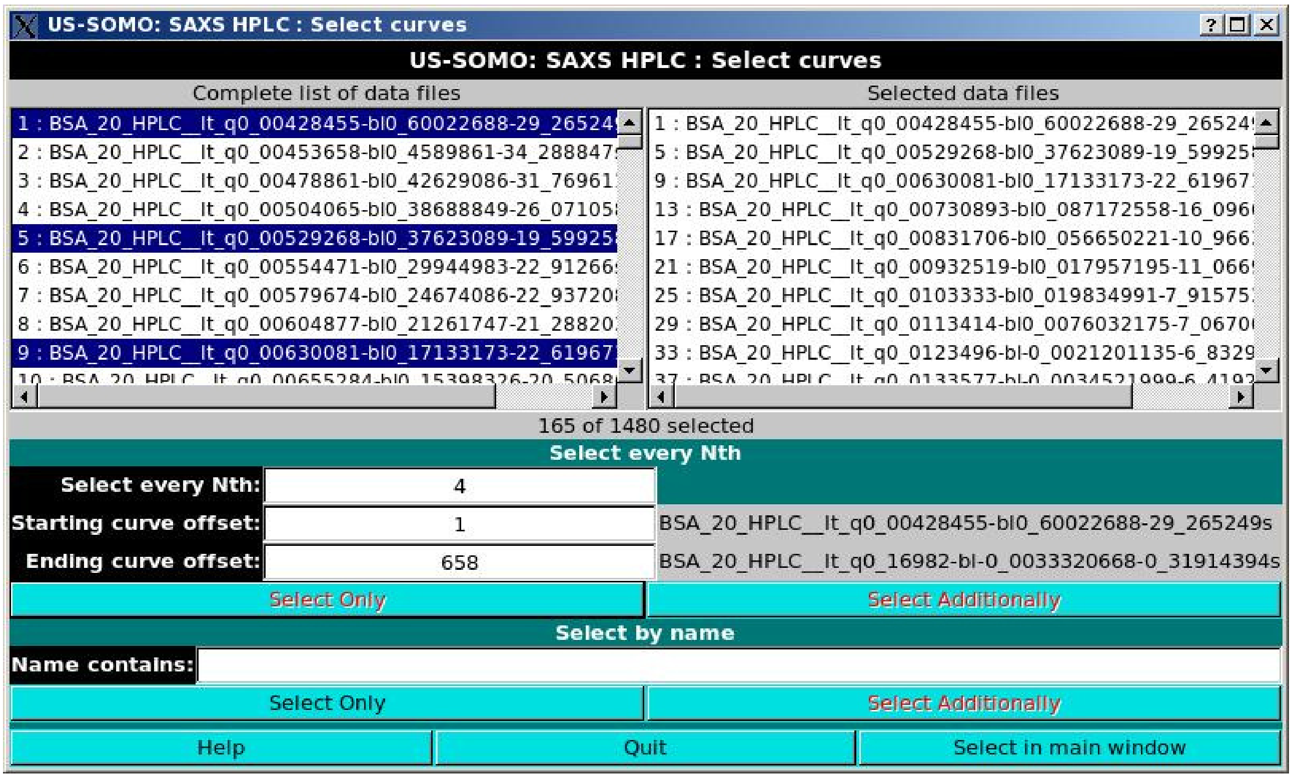

Figure S23 The select module with the “Select every Nth” option utilized. Pressing “Select in the main window” will then perform the selection operation in the main window.

At the end of the process, the graph will display the common centres and widths of the resulting Gaussians as vertical and horizontal bars, respectively (see Fig. S22). The Gaussians can be saved to the current selected directory with the “Save” button (with extension -gauss.dat for Gaussians of single files and -mgauss.dat for Gaussians of multiple files), and accepted with the “Keep” button.

“Global fit”, which becomes available instead of the “Fit” button once a series of chromatogram is selected and after at least an initial set of Gaussians is accepted/loaded, will instead optimize all the centres and widths of each Gaussian along all the chromatograms to common values for each family of Gaussians. The operation is controlled by the same pop-up "Fit" panel as for the single chromatogram case (Fig. S20), but only the LM method is currently available. As this procedure can be quite computationally intensive, it is best to conduct it on a restricted number of chromatograms (for instance using the same “Nth” files of the previous step), save and keep the results, and apply them to all the chromatograms by pressing "Select all" and then “Global Gaussians”. The results of a global fit and their associated residuals can be seen in Fig. S24, while Fig. S25 shows the global Gaussians results after applying the global fit parameters found on a subset of data to all chromatograms. Fig. S26 shows a single decomposed chromatogram. Pressing the “3D” button will bring up a 3D plot of the data, allowing easier detection of potential fitting issues (Fig. S27).

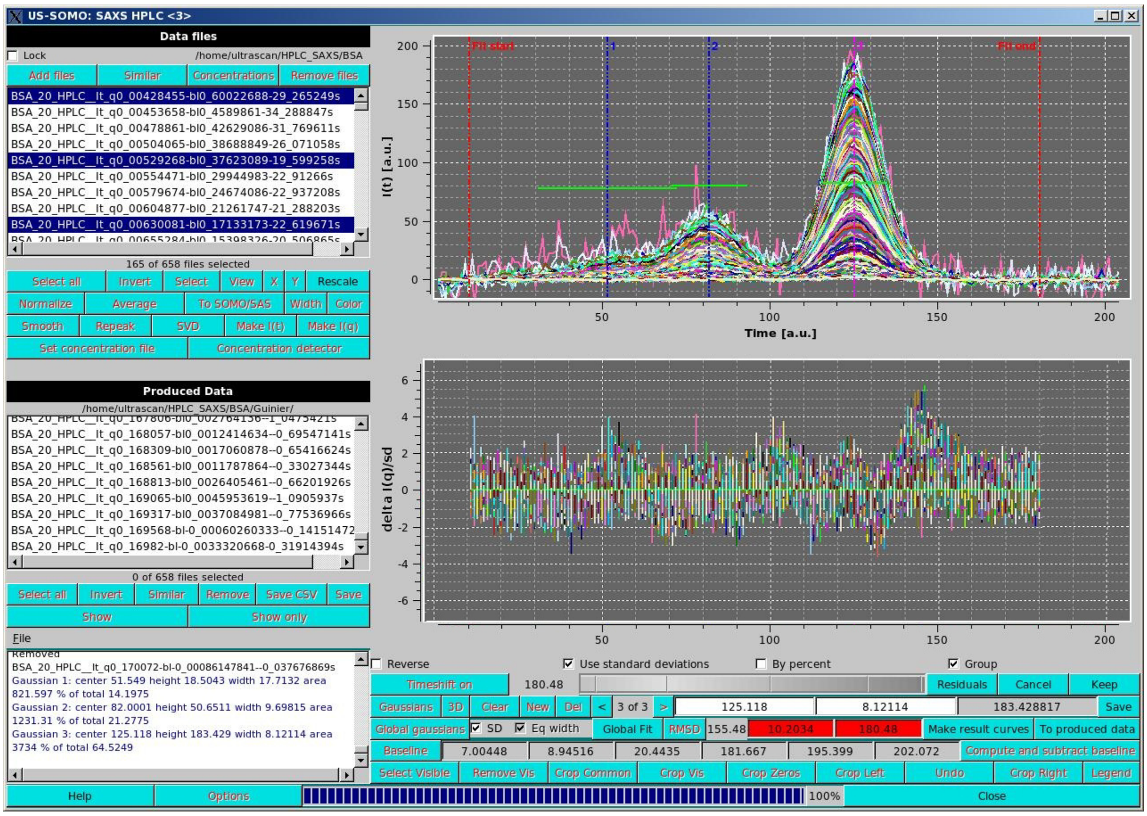

Figure S24 Top panel: global fit results optimizing the parameters found with global Gaussians to multiple selected files; SD weighting and equal widths were also selected. Bottom panel: the reduced residuals for each chromatogram are plotted superimposed to each other ("Use SDs" and "Group" checkboxes selected).

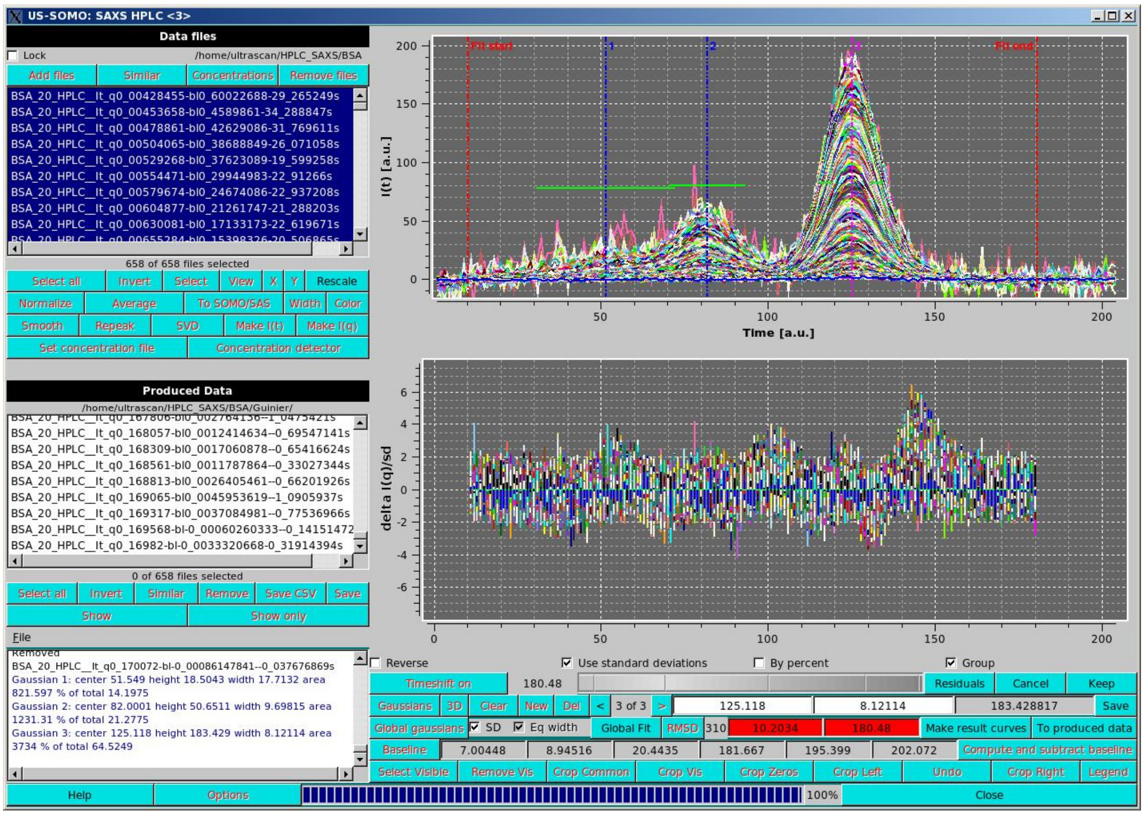

Figure S25 Top panel: global Gaussians results applying the parameters found on global fit to all selected files; SD weighting and equals widths were also selected. Bottom panel: the reduced residuals for each chromatogram are plotted superimposed to each other ("Use SDs" and "Group" checkboxes selected).

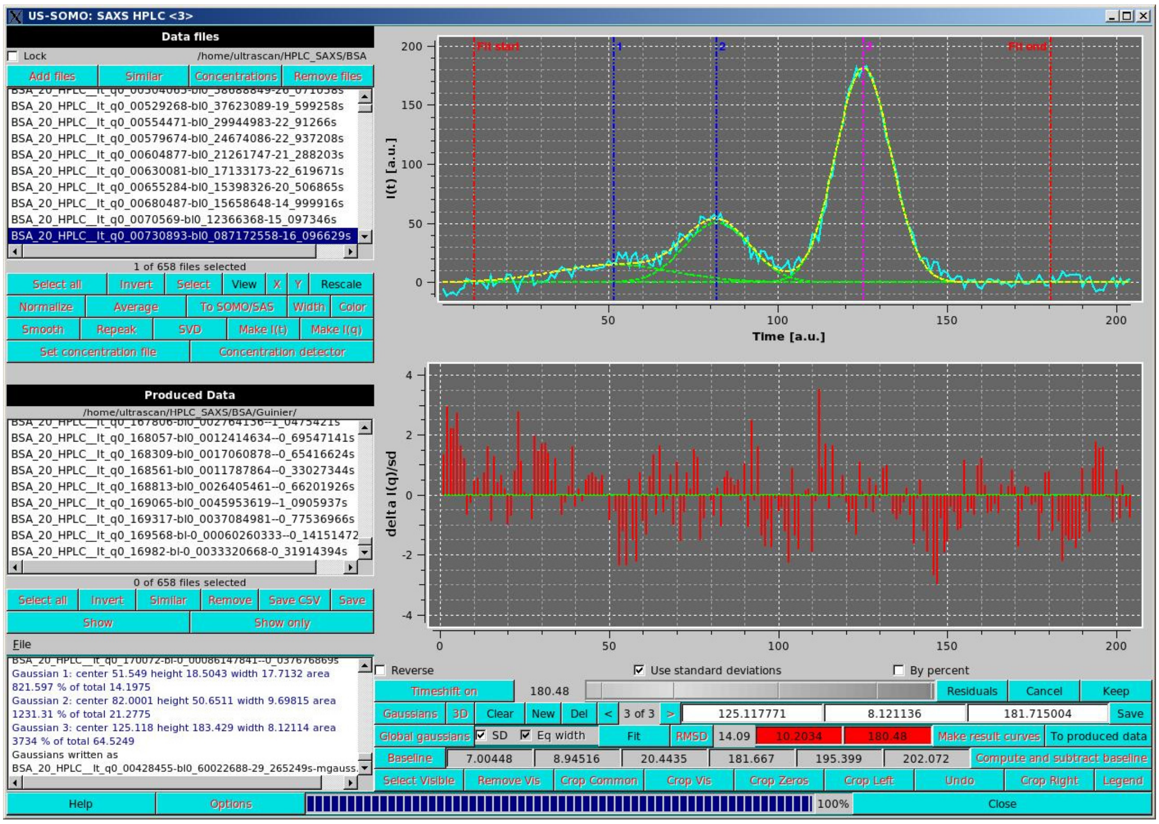

Figure S26 Top: single *I(t) vs. t* chromatogram with superimposed its final Gaussians. Bottom: the associated residuals.

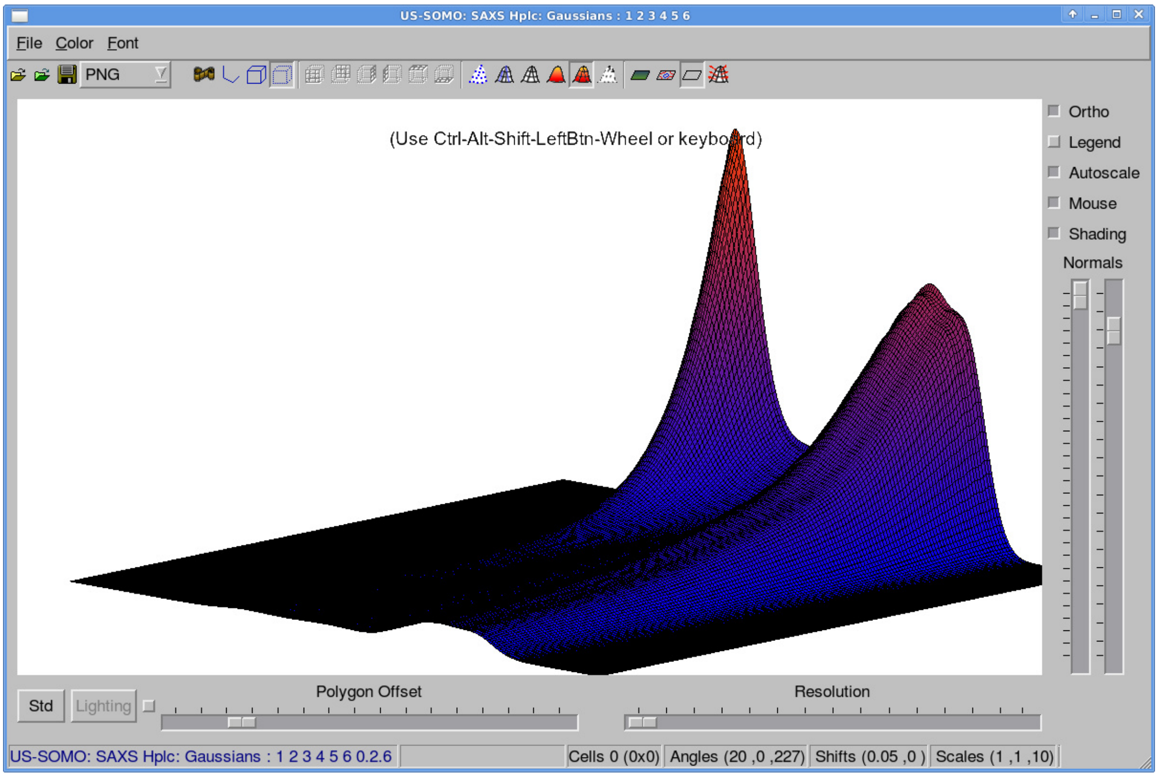

Figure S27 A 3D plot of Global Gaussians. This interactive plot can show any selected set of the Global Gaussians over any collection of curves. The interface is fully interactive for rotations, scaling and zooming along with multiple display and save controls. Its utilization is helpful for visualizing the quality of the global fit.

Each individual Gaussian is defined by three numbers: the amplitude, width and centre. As such, they are not "curves" in the sense of the loaded files, which are collections of data points. Therefore, the Gaussians can not be visualized with the facilities of the program outside of Gaussian or Global Gaussian modes. To allow the visualization of the Gaussians, the "Produce Gaussians as curves" button is provided which produces curves of individual Gaussians and their sum. This is available in either Gaussian or Global Gaussian modes. The resulting curves are collections of data points that can be visualized outside of the Gaussian modes. The Global Fit method requires a simultaneous fit of all the selected curves. This is internally represented by joining all the selected curves along the time/frame dimension to produce one long curve. Of course, each curve is generally on the same time/frame axis range, so to maintain increasing time/frame numbers, curves subsequent to the first one are placed into the joined curve with an offset in time/frame. To visualize the joined curve and the Global Gaussian fit to the joined curve the "Make result curves" button is provided. This will create the joined curve along with the joined Global Gaussian fit as a pair of curves that can be visualized outside of the Global Gaussian mode.

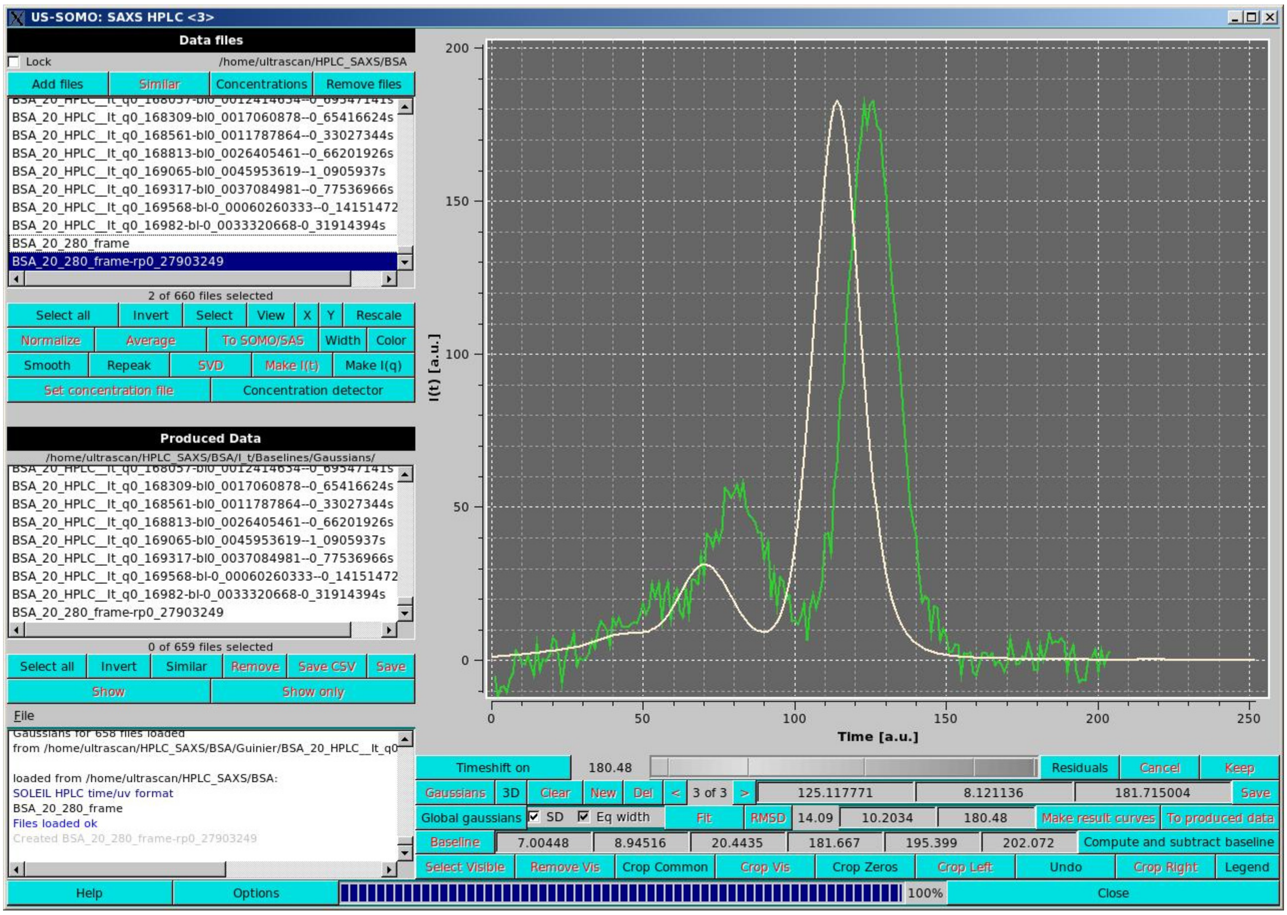

Figure S28 Re-peaked concentration chromatogram (white) shown together with the target *I(t) vs. t* chromatogram (green).

If available, a concentration chromatogram deriving from UV or refractive index monitors can now be processed. After uploading a suitable file with the “Add” button, the first operation is to rescale it to one of the high intensity but relatively low-noise *I(t) vs. t* chromatograms. This is done by selecting the two files,

and pressing “Repeak”, which will bring up a small window asking to identify the target chromatogram (in case multiple were selected). The result of a re-peak operation is shown in Fig. S28, and the scaling factor is added to the concentration dataset filename. After re-peaking, the concentration chromatogram usually must be time-shifted to align its peaks to the *I(t) vs. t* chromatograms using the “Timeshift on” button. Again, two files must be selected, one is the concentration data, the other belonging to the *I(t) vs. t* set (the file used for re-peaking is normally used for this operation). On pressing the “Timeshift on” button, a pop-up window will ask to select which chromatogram is the reference one. The alignment is then performed manually by left-clicking and moving the mouse over the grey-shades wheel bar below the graphics window until the two chromatograms are best aligned. The value of the timeshift is reported in the field next to the “Timeshift on” button. “Cancel” or “Keep” will stop the operation or keep the time-shifted data, respectively (Fig. S29). The produced data will have the timeshift value added to its filename on saving. Pressing “Set concentration file” will then associate the time-shifted data to the *I(t) vs. t* data under analysis.

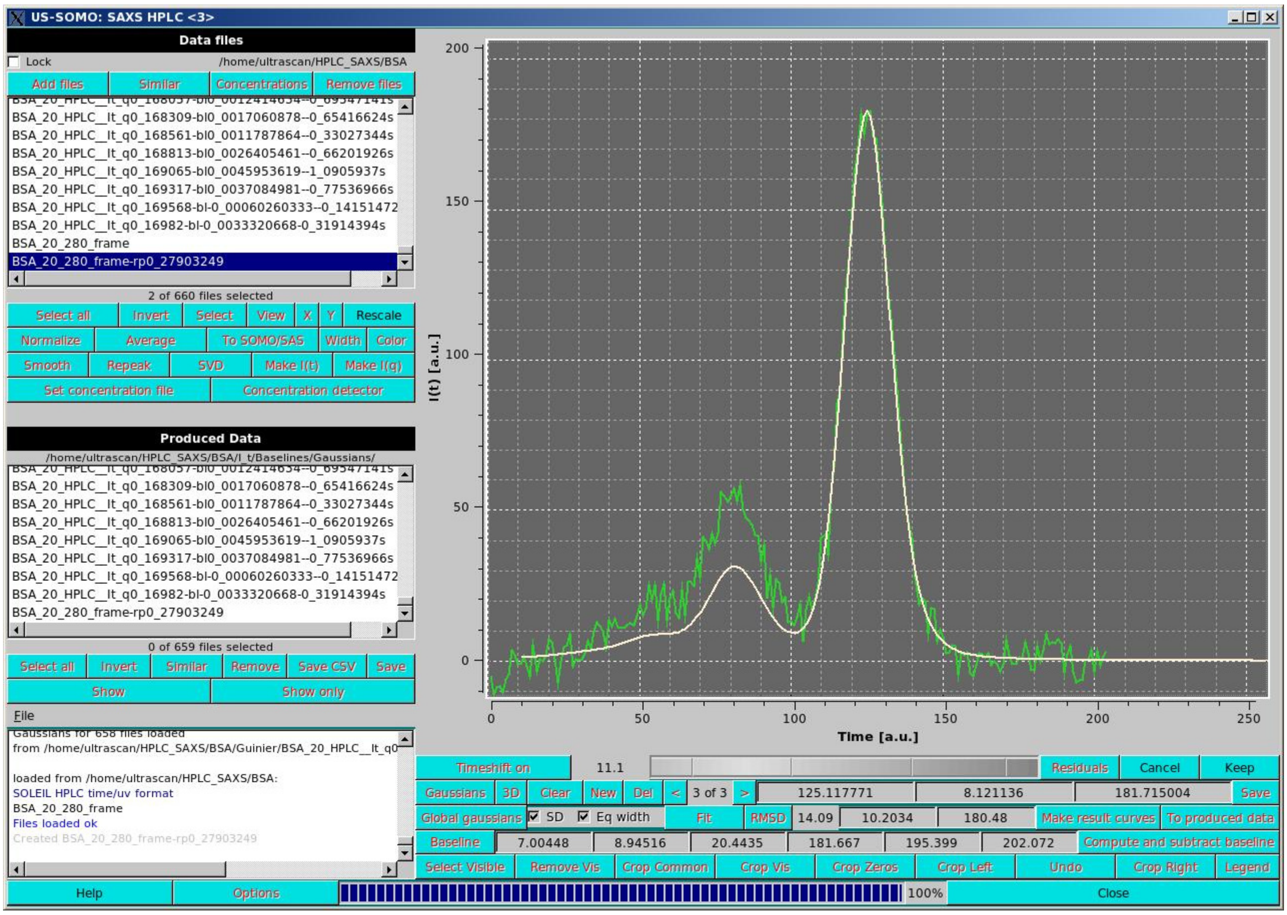

Figure S29 Re-peaked concentration chromatogram (white) shown after time-shifting superimposed to the target *I(t) vs. t* chromatogram (green). Note that the higher intensity of the first peak on the *I(t)* chromatogram (around frame 80) as compared to that on the re-peaked UV data is simply associated with the higher mass of the dimer eluting under this peak.

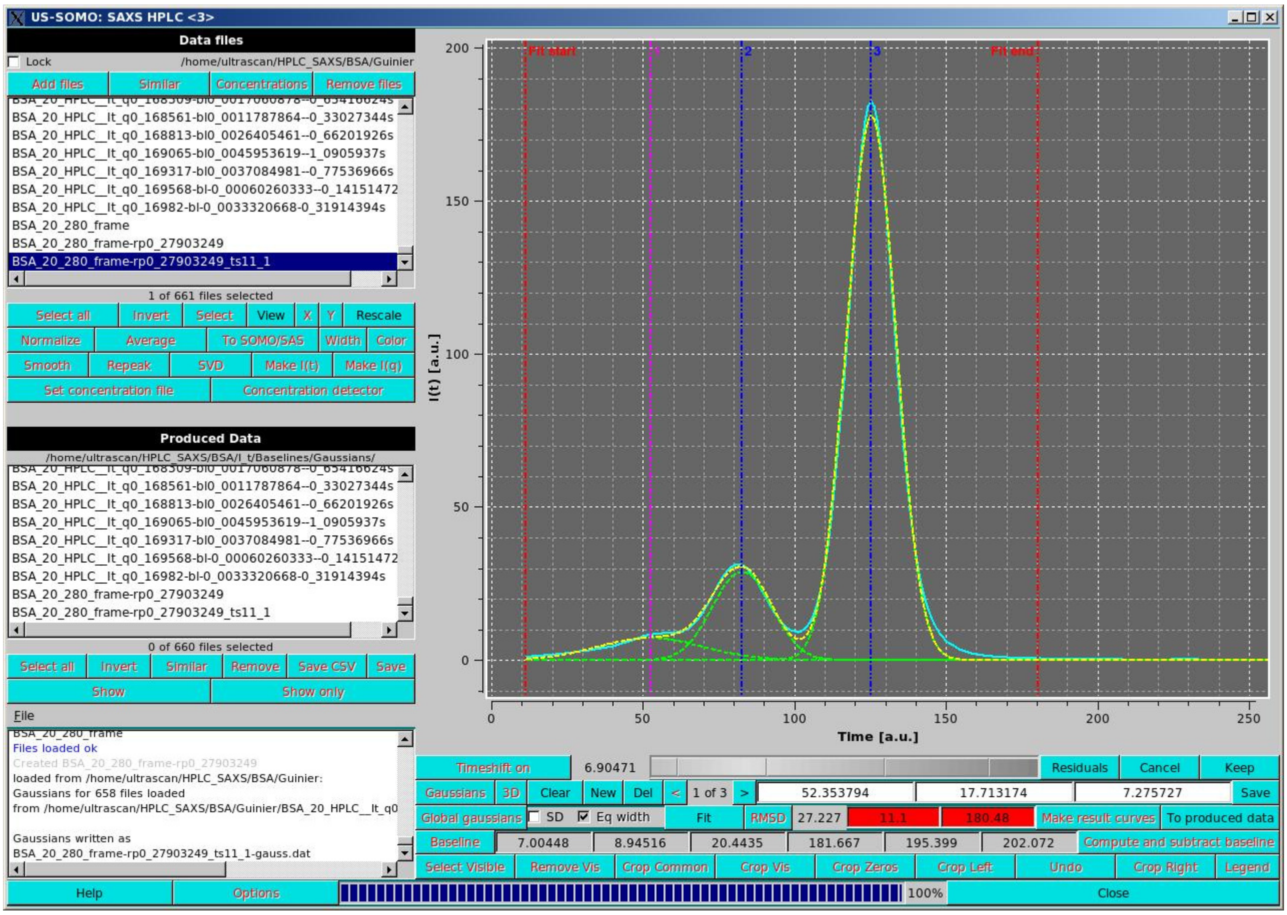

Figure S30 Concentration chromatogram after Gaussian fit.

The re-peaked, time-shifted concentration chromatogram can be now fitted with Gaussians (Fig. S30), using for initialization the set derived from the *I(t) vs. t* chromatograms (note: it is mandatory that the same number of Gaussians be used for both the concentration and *I(t) vs. t* chromatograms). This is done by first selecting only the concentration chromatogram and then pressing "Gaussian", which will show the last Gaussians used. Pressing "Fit" will then bring up the Fit window (Fig. S20) and an initial round is done by keeping fixed both the position and widths. If necessary, a refinement can be done by keeping fixed the widths determined from the SAXS data and allowing only a limited shift to a % of the initial values (suggested: 2-3%). However, if significant band-broadening occurs between the concentration detector and the SAXS capillary, the widths must also be re-optimized. A band broadening correction routine will be implemented in a future release. However, the agreement with the two major peaks in Fig. S30 shows that, at least in the case of this dataset, this is not a major issue. The "Save" and "Keep" buttons must be then pressed to store and associate the resulting Gaussians to the concentration chromatogram. On re-generating the *I(q) vs. q* frames (see below), each concentration Gaussian peak will be mapped onto the corresponding *I(t) vs. t* peaks.

Every time that more than one chromatogram has been fitted with Gaussians, the "Make I(q)" button becomes available. Pressing it will produce a series of *I(q) vs. q* curves for each Gaussian peak for each frame of the chromatogram on which the global operations have been carried out. An option panel in a pop-up window will allow several choices (see Fig. S31).

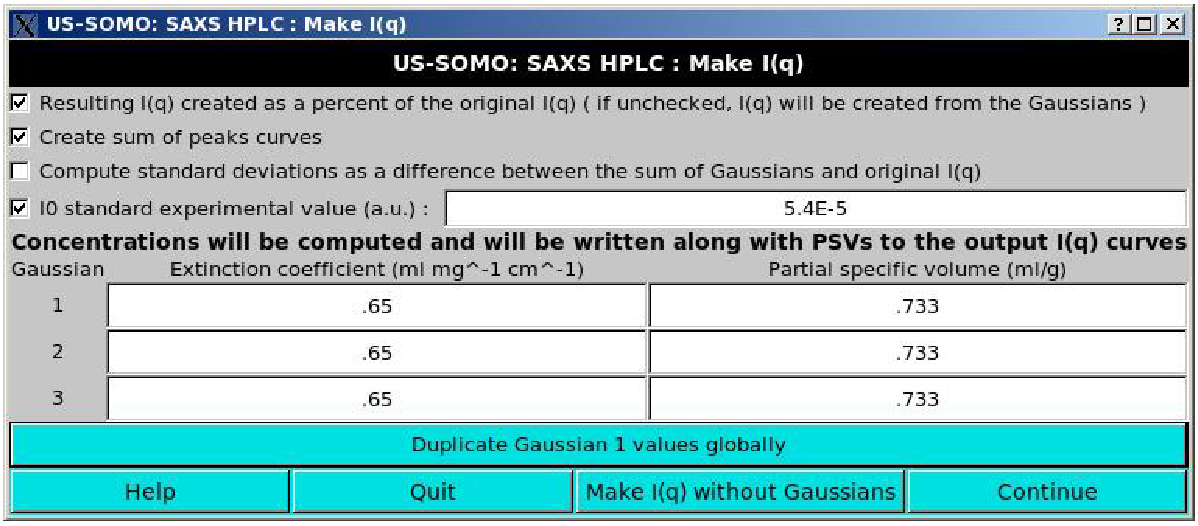

Figure S31 Options available on saving the reconstructed *I(q) vs. q* data.

Since a non-zero baseline is usually due to spurious signals, such as the accumulation of material on the capillary walls during a chromatographic separation, baseline back-addition after Gaussian decomposition is not allowed in non-expert mode operation. The baselines are only added to the sums of Gaussians to verify that the original *I(q) vs. q* frames have been correctly reconstructed (see below). If a baseline was established and subtracted, each produced dataset will be marked with "bs" to indicate that. Three options are present in this panel. The first checkbox controls the produced *I(q) vs. q* data for each Gaussian point in each frame: they can be saved as the actual Gaussian value, or as a % of the original data point based on each Gaussian contribution to the fit (default option). The first option (checkbox unchecked) produces smoothed *I(q) vs. q* curves, while the second (checked) preserves the irregularities present in the original data. The second checkbox allows to check that the individual Gaussian will add back up reconstructing the original *I(q) vs. q* curves. If checked, their point-wise sum can also be saved, as either sum(I) (reconstructed values) and sum(G) (pure Gaussians) curves. If baselines were established and subtracted, two sums will be produced, without and with baseline back-addition. In all cases, the original errors associated with each *I(q) vs. q* point in each frame will assigned %-wise to each point in the resulting decomposed *I(q) vs. q* curves for each frame. Alternatively (third checkbox), and only if the % of the original data are chosen for saving, a new set of errors can be computed, and assigned %-wise to each point in the decomposed *I(q) vs. q* curves, by point-wise calculating the difference between the sum of the Gaussians (with baseline added) and the original curve. A normalization factor from a standard sample to be associated with the data can be also entered here.

Finally, if a concentration chromatogram has also been processed a calculated concentration can be assigned to each of the resulting *I(q) vs. q* curves. This is done by entering an extinction coefficient (or a *dn/dc*) for each Gaussian. In addition, a partial specific volume (psv) value, needed for the computation of the $<M>_w$, $<M/L>_{w/z}$, and $<M/A>_{w/z}$ by Guinier analysis in the main US-SOMO SAS module, can be also entered here. The concentration value will be used when the "Normalize" button is pressed after dataset selection.

For clarity, a flow chart of the operations required for the Gaussian decomposition or HPLC-SAXS data is presented below. To make the whole process less cumbersome, some of the steps which have been described here will likely be streamlined in a future release.

- **I** - First, select a single I(t) vs. t pattern with good intensities and not too noisy.

- on this single curve, select the desired number of Gaussians, roughly position them and refine their centers, widths and amplitudes using the ***Fit*** button. Once a satisfactory fit is obtained, press ***Keep*** to store the parameters (centers, widths and amplitudes).

- **II** - Select a subset of I(t) vs. t curves (for instance every Nth curve) and initialize the Gaussian parameters using the previously determined centers and widths (step I) and calculating the amplitudes for each I(t) using the ***Global Gaussians*** button.

- **III** - Starting from the results of step II, determine the common center and width refined values that provide the best global fit to the subset selected in – II - together with the corresponding set of amplitudes for each I(t) using ***Global Fit*.** Press ***Keep*** to memorize the parameters (centers, widths and amplitudes).

- **IV** - Using the previously determined center and width values, calculate all amplitudes for each I(t) using again ***Global Gaussians***.

- **V** – Display all fits and residuals for evaluation. Accept the results by pressing ***Save*** to write these parameters in a file for future retrieval, and then ***Keep*** to actualize the parameters (centers, widths and amplitudes) for making back the I(q) vs q datasets. Otherwise, start a new refinement cycle from step I or II.

Finally, a tree-view of the US-SOMO SAS module windows is presented in Figure S32, below.

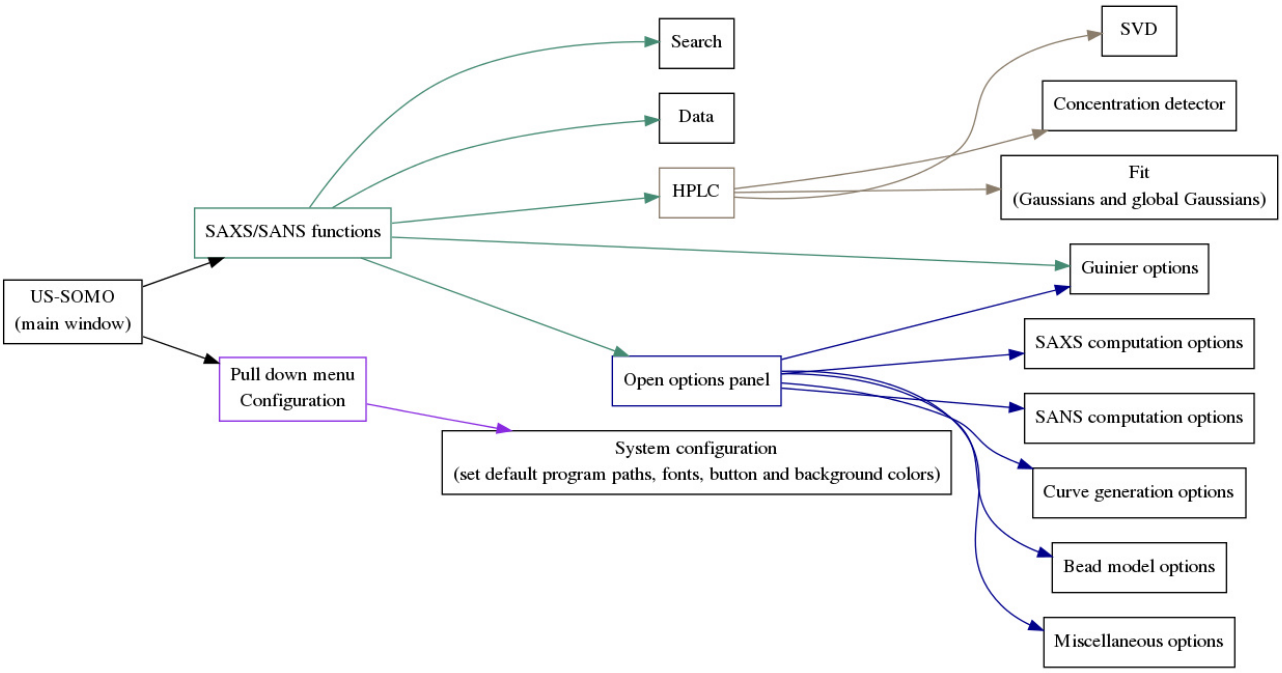

Figure S32 A general tree showing the arrangement of the US-SOMO SAS windows.

Supplementary References

Aster, R. C., Brochers, B., Thurber, C. H. (2005). Parameter Estimation and Inverse Problems. Elsevier Academic Press.

Glatter, O. (1977). *J. Appl. Cryst.* **10**, 415-421.

Glatter, O. & Kratky, O. (1982). Editors. Small-Angle X-ray Scattering. New York: Academic Press.

Hansen, S. (2000). *J. Appl. Cryst.* **33**, 1415–1421.

Ilavsky, J. & Jemian, P. R. (2009). *J. Appl. Cryst.* **42**, 347-353.

Lawson, C. L. & Hanson, R. J. (1995). Solving least squares problems. SIAM.

Petoukhov, M. V., Franke, D., Shkumatov, A.V., Tria, G., Kikhney, A. G., Gajda, M., Gorba, C., Mertens, H. D. T., Konarev, P. V. & Svergun, D. I. (2012). *J. App. Cryst.* **45**, 342-350.

Rayleigh, L. (1911). *Proc. Royal Soc. London* **A84**, 25-46.

Stuhrmann, H. B. (1970). *Acta Cryst.* **A26**, 297-306.

Stuhrmann, H. B., Koch, M. H., Parfait, R., Haas, J., Ibel, K. & Crichton, R.R. (1977). *Proc. Natl Acad. Sci. USA* **74**, 2316-2320.

Svergun, D. I. & Koch, M. H. J. (2003). *Rep. Prog. Phys.* **66**, 1735-1782.

Waasmaier, D. & Kirfel, A. (1995). *Acta Cryst.* **A51**, 416-431.

Williamson, T. E., Craig, B. A., Kondrashkina, E., Bailey-Kellogg, C. & Friedman. A. M. (2008). *Biophys J.* **94**, 4906–4923.
